# Physical Activity Determinants Among Adolescents With Mild Intellectual Disability in Dutch Practical Education: Exploratory Sequential Mixed Methods Study

**DOI:** 10.2196/89518

**Published:** 2026-09-30

**Authors:** Maria Inês Ribeiro, Monique Simons, Daniëlla van Uden, Laura Genga, Pieter Van Gorp

**Affiliations:** 1 Information Systems, Industrial Engineering and Innovation Sciences, Eindhoven University of Technology, Atlas, Eindhoven, North Brabant, The Netherlands, +31402472062; 2 Consumption and Healthy Lifestyles, Department of Social Sciences, Wageningen University & Research, Wageningen, The Netherlands; 3 Health and Society, Department of Social Sciences, Wageningen University & Research, Wageningen, The Netherlands

**Keywords:** physical activity, adolescents, intellectual disability, behavioral determinants, participatory research, Theoretical Domains Framework, COM-B, digital health interventions, Capability, Opportunity, Motivation-Behavior

## Abstract

**Background:**

A sedentary lifestyle is a global health crisis that contributes to chronic disease and premature mortality. Adolescents with mild intellectual disability attending Dutch practical education (PrO) schools face elevated health risks due to low levels of physical activity (PA) and limited conceptual, social, and practical skills. Digital health interventions offer scalable solutions, but require contextual adaptation informed by formative research. However, understanding of PA behavior in this population and context remains limited because existing research relies on partial lenses and lacks integration of theory, participatory methods, and multistakeholder insights.

**Objective:**

We aimed to identify and prioritize PA determinants among adolescents with mild intellectual disability in Dutch PrO schools, using a theory-driven, participatory, and multistakeholder approach to inform the contextual adaptation of digital health interventions.

**Methods:**

We used an exploratory sequential mixed methods study design integrating context mapping and concept mapping. Inclusive participatory methods were co-designed with PrO schools for adolescents. In the first qualitative phase, PA determinants were identified through data collected from experts (n=13), teachers (n=8), and adolescents (n=83‐90). Qualitative data from all stakeholder groups were integrated and analyzed using a framework approach grounded in the Theoretical Domains Framework and the Capability, Opportunity, Motivation-Behavior model. In the subsequent quantitative phase, the PA determinants identified across all data sources were translated into inclusive pictorial rating instruments and prioritized from the adolescents’ perspectives using a 7-point Likert scale ranging from barriers to facilitators. Quantitative analyses included descriptive statistics and hierarchical clustering to examine perceived importance and consensus across determinants.

**Results:**

Qualitatively, 29 PA determinants were identified and mapped to Capability, Opportunity, Motivation-Behavior components: opportunity (n=15), motivation (n=8), and capability (n=6). Experts and teachers focused on capability and opportunity barriers (eg, impaired skills and cognitive fatigue), while adolescents emphasized motivation and social opportunity facilitators (eg, fun activities, enjoyment, and peer or family support). Quantitatively, adolescents’ ratings showed a positive bias toward facilitators (mean 5.09/7, SD 1.72), and facilitators showed greater consensus than barriers (*ρ*=0.74). Adolescents rated several determinants as facilitators that experts and teachers emphasized as barriers (eg, those related to impaired skills and self-efficacy).

**Conclusions:**

Three meta-inferences informing future intervention adaptation were derived: (1) substantial individual variation exists in adolescents’ perceptions of PA determinants, (2) school-related barriers are highly individualized, and (3) social and emotional facilitators tend to be relatively more shared across adolescents. Integrating findings suggests a dual approach to promoting PA in this population and context: personalized strategies for individual barriers and group-level strategies for shared facilitators. Digital health interventions seem promising to provide accessible and inclusive means of delivering this dual approach at scale.

## Introduction

A sedentary lifestyle is recognized as a global health crisis, as insufficient physical activity (PA) increases the risk of many health conditions [1]. Reports indicate that 81% of adolescents worldwide fail to meet the World Health Organization guideline of 60 minutes per day of moderate-to-vigorous PA [2]. Adolescents with mild intellectual disability face additional challenges in adopting and maintaining an active lifestyle due to limited conceptual, social, and practical skills [3]. As a result, they have a higher risk and prevalence of health conditions, such as overweight and obesity, than their peers [4]. In the Netherlands, about 30,000 adolescents attend practical education schools (Praktijkonderwijs, PrO), an education track for students aged 12 to 18 years with mild intellectual disability (IQ 55‐80) [5,6]. Although sedentary behavior is clearly linked to poor health, PA interventions for this population remain scarce, and evidence of their effectiveness is inconsistent [7].

Digital health interventions show promise for addressing the needs and lived experiences of adolescents with mild intellectual disability [8-10]. Evidence indicates that they can effectively promote PA among youth, while offering equitable and inclusive health support [11]. Their potential lies in three features: accessibility, scalability, and the ability to personalize content to individual needs and context [11]. Recent research on digital interventions for people with intellectual disability highlights both their feasibility and the need for personalization to individuals and their support environment [12-14]. Therefore, interventions for adolescents in PrO schools should be informed by a comprehensive needs assessment to identify behavioral determinants in this population and context [15-18].

Previous research on PA determinants among adolescents with mild intellectual disability appears insufficient to inform the adaptation of digital health interventions for PrO students [19,20]. Prior studies do not consistently identify the same determinants*,* and this inconsistency is most evident for interpersonal and environmental determinants, which are rarely replicable between studies and differ from the PrO context [19,20]. We argue that this knowledge gap persists because prior research has addressed it only through partial lenses: studies have drawn on theoretical underpinnings, inclusive methods that elicit adolescents’ own perspectives, or triangulation across relevant stakeholders and contexts, but have failed to integrate these 3 complementary components within a single design.

One lens of prior research has drawn on behavioral theory to examine PA determinants among adolescents with mild intellectual disability. For example, studies such as those grounded in the Theoretical Domains Framework (TDF) and the Capability, Opportunity, Motivation-Behavior (COM-B) model offer ways to systematically identify barriers and facilitators to PA, compare findings across studies, and inform the adaptation of evidence-based digital health interventions [17,21-23]. Still, a theoretically informed strand of literature in this target population and context remains limited.

A second lens of prior research has studied PA behavior from the adolescents’ own perspective using quantitative and qualitative methods. Quantitative methods, such as movement sensors and questionnaires, objectively assess PA levels in large populations but only in relation to a predefined limited set of behavioral determinants [24-29]. Qualitative methods, including interviews and focus groups, allow a more open exploration of determinants that matter to adolescents themselves and are often aligned with participatory health research principles [21,22,30-34]. While involving the adolescents in research has been shown to improve intervention effectiveness [35,36], prior studies reported that adolescents with intellectual disability often struggled to express their opinions or participate in rich discussions [21,37]. This points to a need for more inclusive methods that use concrete and creative ways of eliciting these adolescents’ perspectives [37-39].

A third lens of prior research has highlighted that PA determinants arise not only from personal characteristics, but also from the dynamic interplay between adolescents and broader systems, such as schools and policy [40,41]. From this systems science perspective, triangulation across multiple data sources supports a comprehensive understanding of PA determinants [42]. Existing studies involving parents and teachers identified different PA determinants than those reported by adolescents, suggesting that no single stakeholder perspective captures the full range of factors shaping PA behavior [21,30,32,33]. Therefore, studies that include only indirect stakeholders or only adolescents provide only partial evidence. A multistakeholder approach is thus critical, as it can capture the whole system rather than focusing only on individual perceptions of behavior determinants [40,41].

Hence, this research study integrates these 3 complementary lenses to identify and prioritize PA determinants among adolescents with mild intellectual disability who attend PrO schools in the Netherlands to inform the future adaptation of digital health interventions.

## Methods

### Context

This research was conducted as part of the “Healthy Lifestyle for Low Literate Teenagers” (LIFTS) project (2023‐2028), which promotes healthy lifestyles among adolescents with mild intellectual disability in Dutch PrO schools [43]. LIFTS operated as a participatory action research consortium, including PrO school adolescents, teachers, and other expert stakeholders [44]. This research study focuses on PA as a healthy lifestyle behavior.

### Participants

Sampling targeted multiple stakeholders, namely: PrO school adolescents, teachers, and experts (ie, professionals in the domain of PrO context and health promotion such as school managers and municipality health professionals). The sample size was determined a priori based on feasibility within the LIFTS project and school context, aiming for diverse, information-rich perspectives relevant to the PrO context. Sampling was not extended iteratively to reach coded saturation or statistical representativeness, but meaning saturation was assessed during analysis and was supported by the absence of new determinants or perspectives in the later stages of coding and interpretation [45]. In line with Arnstein’s Citizen Participation Ladder, involvement of these stakeholders ranged from consultation to partnership [46].

#### Experts and School Managers

From March to September 2024, experts and school managers were recruited via email and through convenience sampling via the LIFTS network [47]. They had a consulting role by providing their preconceptions of PA determinants among adolescents with mild intellectual disability in the PrO context. Additionally, school managers partnered with researchers to cocreate inclusive research methods for the adolescents.

#### Teachers

In September 2024, teachers were recruited face-to-face via purposive sampling through their school managers. Teachers had both a role as consultants and partners, providing preconceptions on PA determinants and cofacilitating inclusive research activities with the adolescents (eg, helping with classroom management, student support, and instruction clarification). Researchers conducted a series of in-person and online meetings with the teachers to frame this study’s aim and explain the procedure. This study’s protocols cocreated with the school managers were provided to the teachers, including a study guide explaining activities, materials, and information letters.

#### Adolescents

From October to November 2024, adolescents enrolled in PrO schools in the LIFTS network, aged 12 to 17 years, were recruited via class-based purposive sampling with the support of school managers. They were involved as consultants on their own perceived PA determinants. Intact class groups of up to 16 students from years 1 to 4 of PrO schools were selected. While research guidelines recommend 10 to 12 participants per group, the existing class structure and staffing limitations made it impractical to divide classes into smaller groups [48]. The sample size was defined to offer sufficient diversity within the target population.

### Study Design

This study used an exploratory sequential mixed methods design [49], guiding the integration of the methodological lenses of theoretical grounding, participatory research, and multistakeholder triangulation. It was reported according to COREQ (Consolidated Criteria for Reporting Qualitative Research) and GRAMMS (Good Reporting of a Mixed Methods Study) guidelines for qualitative and mixed methods research (Checklists 1 and 2) [50,51]. Figure 1 shows this study’s design. The qualitative and quantitative phases were equally important and complementary: the former identified PA determinants from the perspectives of adolescents, teachers, and experts, and the latter examined and prioritized how the adolescents perceived these determinants as barriers or facilitators at the individual and group level. Integration occurred first between phases, when the identified determinants were transformed into inclusive rating instruments, and again during interpretation, when adolescents’ ratings were compared with the qualitative multistakeholder findings. Together, these phases are needed for informing intervention adaptation.

This study was theoretically grounded in the TDF and COM-B model to systematically identify behavioral determinants through 3 COM-B components and 14 TDF domains [23]. Integrating the Behavior Change Wheel, this study can support future linkage to intervention functions and behavior change techniques [17,52].

Data were collected from March to November 2024. The main researcher (MIR) kept a reflection diary on the participatory methods used with the adolescents, which served to adapt them for subsequent phases iteratively. The third researcher (DvU) analyzed the diary in a separate publication on participatory research for health promotion with PrO students [53].

**Figure 1.**
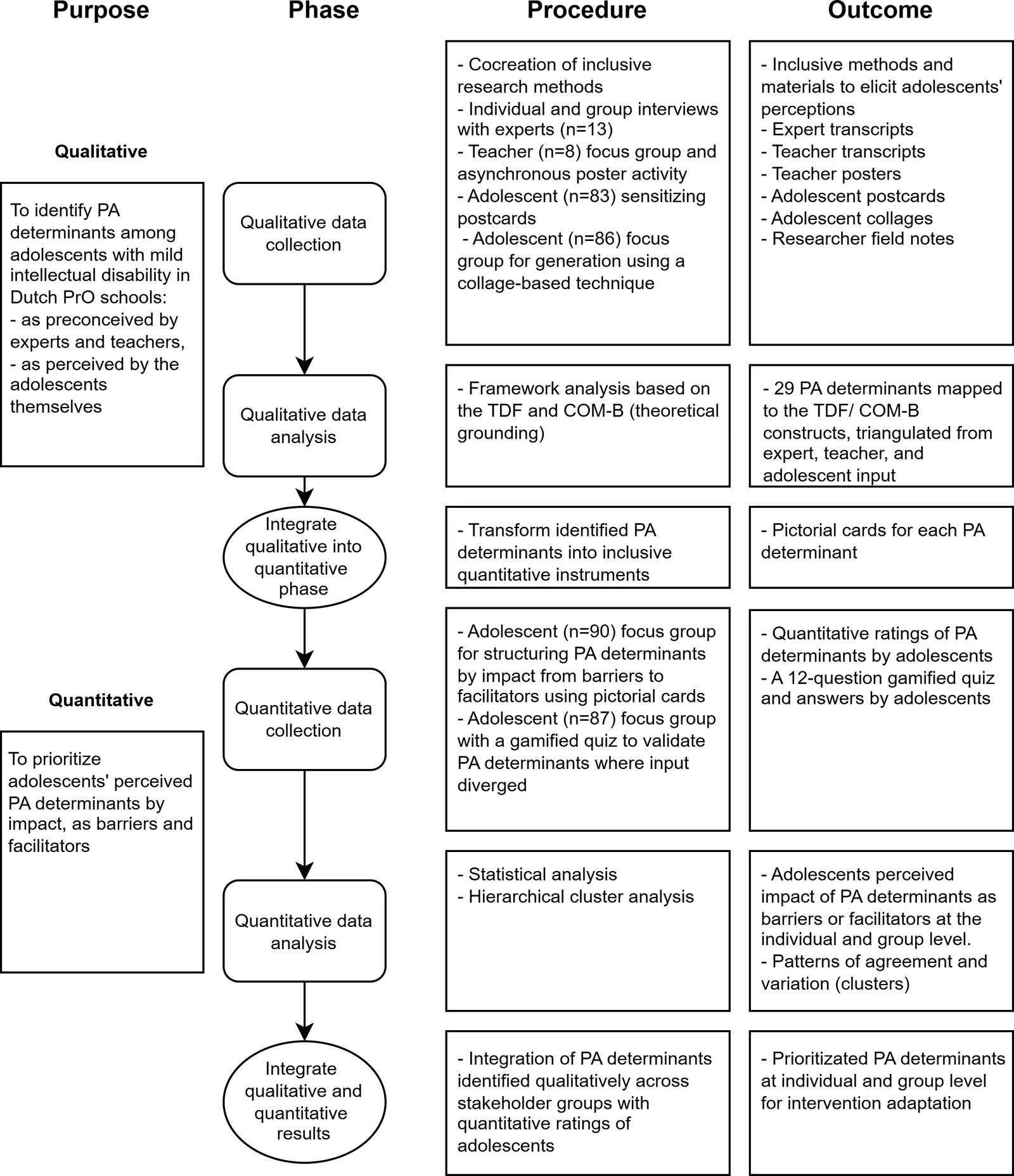
Procedural diagram for the exploratory sequential mixed methods study design aiming to identify and prioritize PA determinants of adolescents with mild intellectual disability in Dutch practical education (Praktijkonderwijs, PrO). COM-B: Capability, Opportunity, Motivation-Behavior model; PA: physical activity; PrO: Praktijkonderwijs (Dutch practical education); TDF: Theoretical Domains Framework.

### Qualitative Phase

#### Overview

In line with the participatory lens, qualitative data collection was informed by Context Mapping, a user-centered design framework that uses creative and visual techniques to elicit tacit knowledge of user needs [54]. We adapted the sensitizing and generation phases in this framework in collaboration with PrO schools to make participation inclusive for the adolescents by priming their awareness of PA behavior and providing design artifacts to express this tacit knowledge.

#### Qualitative Data Collection

The qualitative data collection process comprised 3 stages: preparation, sensitizing, and generation.

##### Preparation

This phase aimed to (1) cocreate inclusive research activities for collecting the adolescents’ perceptions of their PA determinants, and (2) identify preconceptions from experts and teachers. School managers were involved in online and in-person meetings to inform the design of research activities for adolescents. Adaptations of methods and materials included (1) opting for concrete and creative methods (eg, collage and card sorting) rather than abstract methods (eg, open brainstorming and card clustering on undefined categories); (2) arranging familiar environments and trusted facilitators (ie, the teacher); (3) splitting activities into shorter manageable segments; (4) simplifying language in instructions; and (5) accompanying written text with visual representations [55].

Expert preconceptions were collected by MIR in 1-hour interviews, online and in-person at their workplaces. Earlier individual interviews, from March to August 2024, with 6 of the 13 experts were intentionally open and exploratory to coshape the research design and deepen understanding of the PrO context, reflecting this study’s participatory approach. Later in September 2024, two group interviews with the other 7 experts followed a semistructured guide with open questions based on the TDF domains. Table 1 provides the mapping of TDF-based interview questions to higher-level COM-B components. All interviews were audio-recorded and transcribed verbatim using Microsoft Word’s transcription function.

**Table 1.** Semistructured expert interview guide based on the TDF^a^ domains and COM-B^b^ components.

COM-B component	Question based on TDF domains
Opportunity (environment)	Where do students play sports or exercise? What objects do students use when moving? What objects are they missing? How much time do students spend on sports and exercise? What other activities do students have to do or do students want to do that hinder them from playing sports and exercising?
Capability	What do students know or do not know about the importance of sports and exercise? What do students know or do not know about sports and exercise? What skills do students have for sports and exercise? What skills are they still missing? How do students fit sports and exercise into their daily lives? How do students monitor their progress in sports and exercise?
Motivation	What does or does not encourage students to play sports and exercise? How do students feel while playing sports and exercising? How do feelings and emotions influence the sports and exercise of students? How do sports and exercise fit in with the self-image of students? What do students think happens when they play sports and exercise? How much do students like sports and exercise? Do students plan sports and exercise moments? Are there activities they prefer to do that prevent them from exercising? Are there activities they have to do that prevent them from exercising?
Opportunity (social influences)	How do friends make it easier or harder for students to play sports and exercise? How does family make it easier or more difficult for students to play sports and exercise? Who else makes it easier or more difficult for students to play sports and exercise? Who is a role model for students to play sports and exercise? Who is a bad example?

a TDF: Theoretical Domains Framework.

b COM-B: Capability, Opportunity, Motivation-Behavior.

Teacher preconceptions were collected by MIR and DvU in a 1-hour focus group in one PrO school, using posters displaying the TDF-based questions shown in Table 1 (Multimedia Appendix 1). The focus group was audio-recorded and transcribed verbatim using Amazon Transcribe [56]. The same posters were placed in the teachers’ room of another PrO school during a week to collect asynchronous input, as proposed by the school representative.

Following preparation, data collection focused on adolescents’ perceptions. MIR led data collection activities with cofacilitation by PrO teachers, spread over 5 weeks in each PrO school. Activities were integrated into the regular class curricula and schedule, as focus groups. MIR kept brief notes directly on adolescents’ materials or in a notebook and later added key observations to the TDF map during analysis.

##### Sensitizing

This phase aimed to prime adolescents’ awareness of their needs, experiences, and wishes related to PA [54,57]. Teachers distributed daily postcards over 5 days with reflection questions and visual prompts, such as “How do you feel during and after sports and exercise?” An example postcard is provided in Figure 2A, with the remaining available in Multimedia Appendix 2. Students completed the postcards anonymously in under 10 minutes, which were collected and photographed.

**Figure 2.**
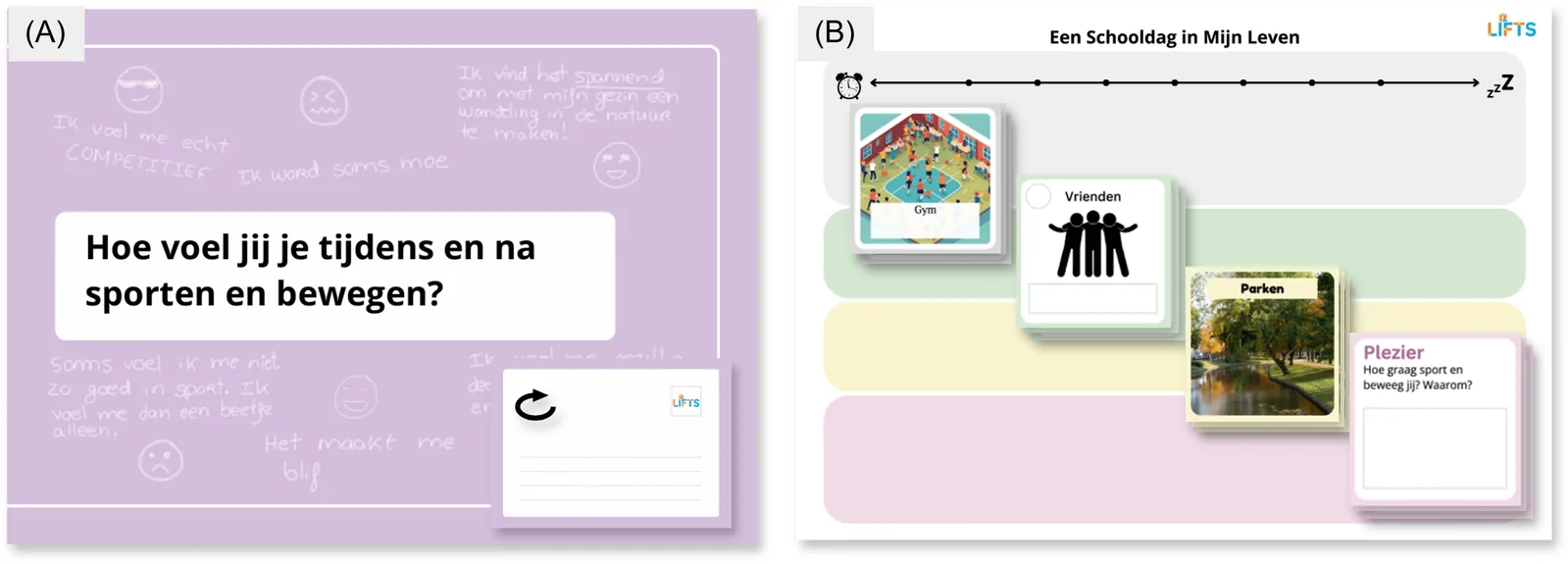
Materials for qualitative data collection with adolescents. (A) Example of sensitizing postcard with question “How do you feel during and after sports and exercise?” and visual prompts for support. (B) Timeline board and prompting cards.

##### Generation

This phase aimed to identify the PA determinants perceived by the adolescents in a 75- to 90-minute focus group guided by the central question: “What makes it easy or hard to participate in sports and exercise for you?” We used inclusive**,** visual methods, including a collage-based timeline board and prompting cards structured on the TDF framework. These materials are illustrated in Figure 2B and fully available in Multimedia Appendix 3. Adolescents mapped their personal routines in a PrO school day, social influences, and environments, then reflected on TDF-based prompt cards related to PA. Adolescents provided demographic details (age and gender) on their collages, which were photographed. When feasible, an additional exercise invited students to share additional PA determinants, either anonymously in writing, in one-on-one conversations, or in plenary discussions.

### Qualitative Data Analysis

This study adopted a subtle realism perspective, assuming that PA determinants exist independently of the research process while recognizing that our understanding of them is shaped by participants’ perspectives and researcher interpretation [58]. Qualitative data were analyzed using the framework approach to systematically analyze and compare input from experts, teachers, and adolescents [59,60].

Qualitative data included expert interview transcripts, teacher focus group transcripts and posters, adolescent postcards and collages, and researcher field notes. We used NVivo (version 15; Lumivero) to support data management and coding [61]. Analysis followed 5 steps: familiarization, identifying a thematic framework, indexing, charting, and mapping and interpretation. MIR first familiarized herself with all qualitative materials. A thematic framework was developed deductively from the COM-B model, with capability, opportunity, and motivation as main themes representing what influences behavior. Within each theme, the TDF domains were used as subthemes to identify the specific factors operating within those COM-B components. Data were then indexed, while the codes for specific PA determinants were generated inductively from participants’ input across stakeholder groups. Thus, the analysis combined a deductive theoretical structure with inductively derived determinants. Finally, coded data were charted into a framework matrix by source and theme to interpret and compare preconceptions from experts and teachers with adolescents’ own perceptions.

Frequencies of mention were not reported because the aim was to identify and compare PA determinants across stakeholder perspectives rather than assess their prevalence, and frequency does not necessarily reflect relevance [60,62]. It should be noted that adolescents’ contributions were primarily captured through visual and written materials. As a result, their input was primarily represented through summaries and examples from these materials rather than verbatim quotations.

The second author (MS) acted as a “critical friend,” reviewing the analysis and promoting reflexive practice, thereby ensuring the robustness and reliability of the findings [63]. Sampling for the qualitative phase was not iteratively extended to reach saturation; instead, meaning saturation was assessed during analysis within the feasible school-based sample [45]. No new determinants or different perspectives across stakeholder group input were discovered in the later stages of analysis, and all data fit within the existing framework.

### Quantitative Phase

#### Overview

The quantitative phase built directly on the qualitative findings: PA determinants identified qualitatively by experts, teachers, and adolescents were translated into inclusive, pictorial quantitative instruments that adolescents could rate and discuss. In line with the participatory lens of this study, this phase used concept mapping, a framework widely used in public health and health promotion [64-66]. It comprised three stages: structuring, representation, and interpretation of identified PA determinants. Structuring and interpretation were implemented during data collection with adolescents, whereas representation was conducted during quantitative analysis by the research team. To support inclusivity, adolescents rated PA determinants on a pictorial Likert scale rather than engaging in abstract sorting tasks, which are difficult for people with intellectual disability [67,68]. Then, a gamified quiz confirmed researchers’ interpretations of adolescents’ perceptions rather than asking students to verify a complex concept representation, which researchers used for analysis.

#### Quantitative Data Collection

##### Overview

The quantitative data collection process comprised the structuring and interpretation stage.

##### Structuring

In a 75- to 90-minute focus group, adolescents rated pictorial cards representing the PA determinants. The central question was “What makes it easy or hard to participate in sports and exercise for you?” Adolescents then rated each card on a board featuring a 7-point Likert scale ranging from “very hard” (1=strong barrier) to “very easy” (7=strong facilitator). Figure 3A illustrates the materials used in this step, fully available in Multimedia Appendix 4. Brief one-on-one conversations were conducted with some participants immediately after the activity, exploring their reasoning behind card ratings and offering insight into their perceptions and decision-making. Ratings were collected by photographing the structuring board, where adolescents wrote their age and gender.

**Figure 3.**
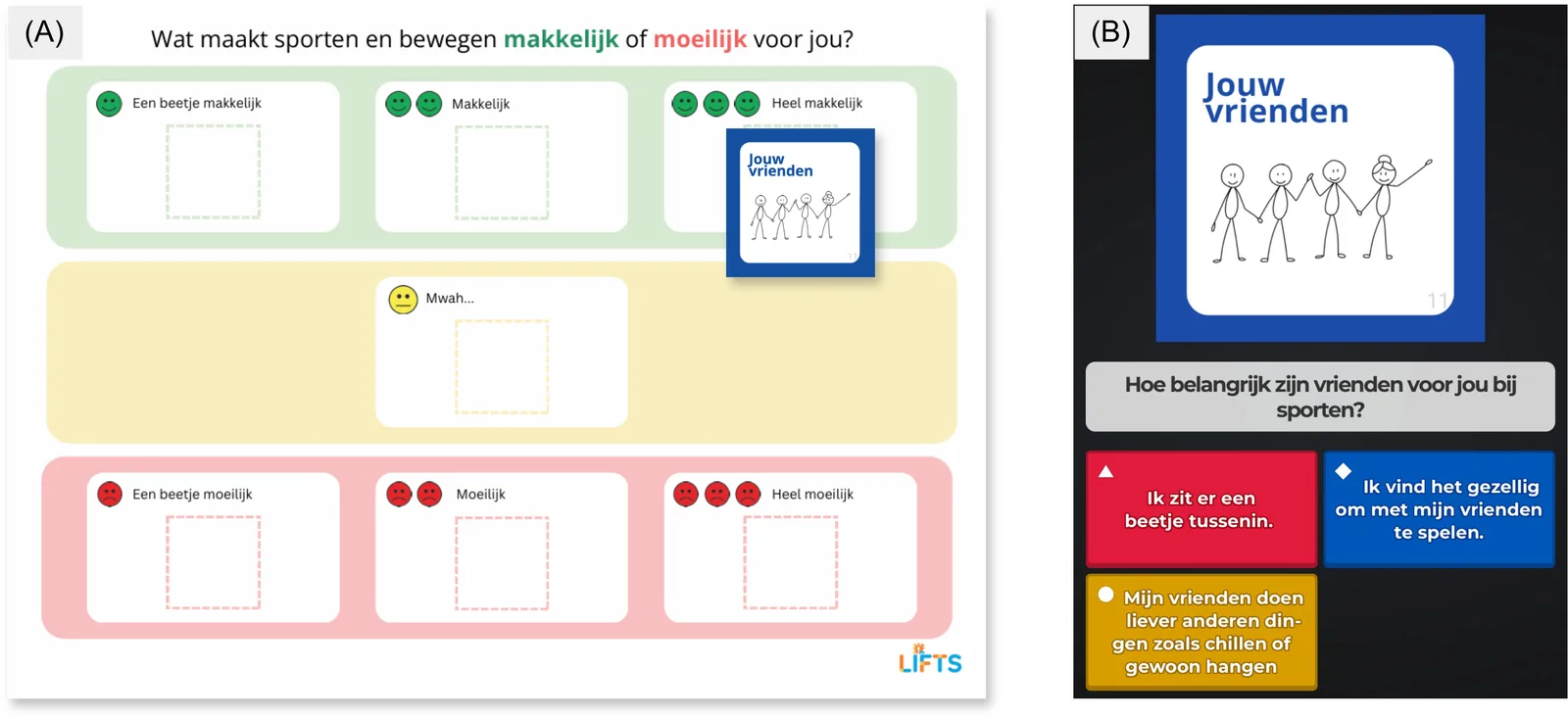
Materials for quantitative data collection with adolescents. (A) Structuring board and a pictorial card representing a PA determinant, that is, support and joint participation with friends and peers; card meaning “your friends.” (B) Example of gamified quiz item.

##### Interpretation

In a 75- to 90-minute focus group, adolescents completed a 12-question gamified quiz, with 3 categorical response options. Quiz items were derived by the research team from patterns observed in adolescents’ quantitative and qualitative input, focusing on determinants where our interpretations of adolescents’ input diverged from preconceptions by experts and teachers. Each question offered categorical options grounded in earlier data and was used to validate the researchers’ interpretation of adolescents’ perceived PA determinants. Figure 3B illustrates the gamified quiz, with the full list of questions and answers available in Multimedia Appendix 5.

### Quantitative Data Analysis

The representation stage of concept mapping was reflected in the quantitative analysis of adolescents’ ratings of PA determinants, which was conducted by the research team. Initially, descriptive statistical analysis was conducted in Python (Python Software Foundation) to examine how adolescents perceived the qualitatively identified PA determinants as barriers or facilitators. The code is available in a public repository [69]. Mean ratings and SDs were calculated overall and by determinant. Within-group agreement (r_wg_) was calculated for overall ratings and by determinants to measure how much adolescents agree on their perceptions of PA determinants from barriers to facilitators [70]. Missing data were not imputed, and analyses used all available observations via pairwise deletion.

Next, hierarchical cluster analysis aimed to prioritize PA determinants at the individual and group level to inform intervention adaptation. The Euclidean distance and average linkage method were used to identify determinant clusters based on mean ratings (from barriers to facilitators) and within-group agreement coefficients (from low to high consensus), revealing patterns in the importance of determinants as perceived by the adolescents individually and as a group that informs the adaptation of interventions. For validation purposes, analysis of data collected in the interpretation step focused on the adolescents’ gamified quiz answers using frequency distributions and percentage agreement.

Importantly, the quantitative phase involved adolescents from the same schools and sample as the qualitative phase and was intended to prioritize PA determinants within the participating context rather than to obtain statistically representative estimates.

### Reflexivity

MIR, supported by DvU, led data collection; both were female PhD candidates at the time. MIR completed secondary and higher education in Portugal, and DvU in the Netherlands. MIR was learning Dutch during data collection, and DvU is a native speaker. Both had limited prior knowledge of PrO and deepened it in the preparation phase of data collection by visiting schools and building relationships. School representatives introduced them to adolescents as university researchers studying their lived experiences related to sport and exercise to help develop health programs suited to the PrO context. No prior relationship existed between the research team and participants.

### Ethical Considerations

#### Overview

This study received approval from the Ethical Review Board of the Technical University of Eindhoven (ERB2024IEIS69). The ethical review and approval covered this study’s protocol, including the consent procedures, privacy and confidentiality measures, and participatory ethics considerations described below.

#### Consent Procedure

Teachers and experts provided written informed consent; adolescents gave oral informed consent, witnessed by their teachers. All information was provided orally and in writing in plain B1-level Dutch with visual supports, in line with the Web Content Accessibility Guidelines [71]. Research activities with adolescents were not audio-recorded because school representatives feared this might discourage participation and limit their freedom to speak. Passive consent was used for caregivers in accordance with Dutch ethical guidelines for low-risk research [72]. No compensation was offered to participants.

#### Privacy and Confidentiality

Only limited personal data were collected (eg, for adolescents, age and gender; for other stakeholders, contact information, main expertise, and audio recordings). Adolescents’ postcards were anonymous, and all other materials were deidentified; physical materials were digitized and destroyed, adults’ audio recordings were deleted after transcription, and only deidentified data were stored in secure restricted-access systems.

#### Participatory Ethics

Participatory research ethics guided the study: building trust, minimizing harm, offering inclusive methods, ensuring confidentiality and informed consent, and creating a safe environment for equal participation [73]. While PrO students may be labeled as a vulnerable population, the adolescents were not treated as inherently vulnerable. Instead, this study sought to enable their participation through inclusive methods that reduced barriers.

## Results

### Participants

Table 2 presents the demographic characteristics of all recruited participants across groups and data collection stages, including experts (n=13), school managers (n=3), teachers (n=8), and adolescents (n=83‐90). Two PrO schools were included (A and B). All participants consented to participate; one adolescent was later withdrawn when their caregiver opted out. Among adolescents, participant numbers varied by stage because some were absent from school during data collection. Gender was approximately balanced (from 38/87, 44%, to 45/86, 52%, male), with a mean age of 14.3 (SD 1.3) years. The proportion of missing responses was low across data collection modalities (76/2610, 3%, of the expected ratings in the structuring activity and 26/1044, 2%, of the expected responses in the validation quiz).

**Table 2.** Participant demographics by stakeholder group and data collection stage, including sample size and, for adolescents, gender distribution and mean age with SD.

Stakeholder group and stages	Sample size	Gender	Mean age (SD)
Experts			
Preparation	13	—^a^	—
School managers			
Preparation	3	—	—
Teachers			
All	8 (n_school A_=4)	—	—
Adolescents			
Sensitizing	83 (n_school A_=48)	—	—
Generation	86 (n_school A_=44)	45 male; 41 female	14.3 (1.3)
Structuring	90 (n_school A_=45)	45 male; 45 female	14.3 (1.3)
Interpretation	87 (n_school A_=40)	38 male; 49 female	14.3 (1.3)

a Not available.

### Qualitative Findings

#### Overview

We identified 29 PA determinants (codes) indexed to COM-B components (themes) and TDF domains (subthemes), as illustrated in Figure 4. Each determinant was assigned to a primary TDF domain, with some also linked to secondary domains (Table S1 in Multimedia Appendix 6). To facilitate comparison across stakeholder groups, data from experts, teachers, and adolescents were synthesized in a framework matrix, summarizing data extracts, participant quotations, and researcher observations for each determinant (Table S2 in Multimedia Appendix 6).

**Figure 4.**
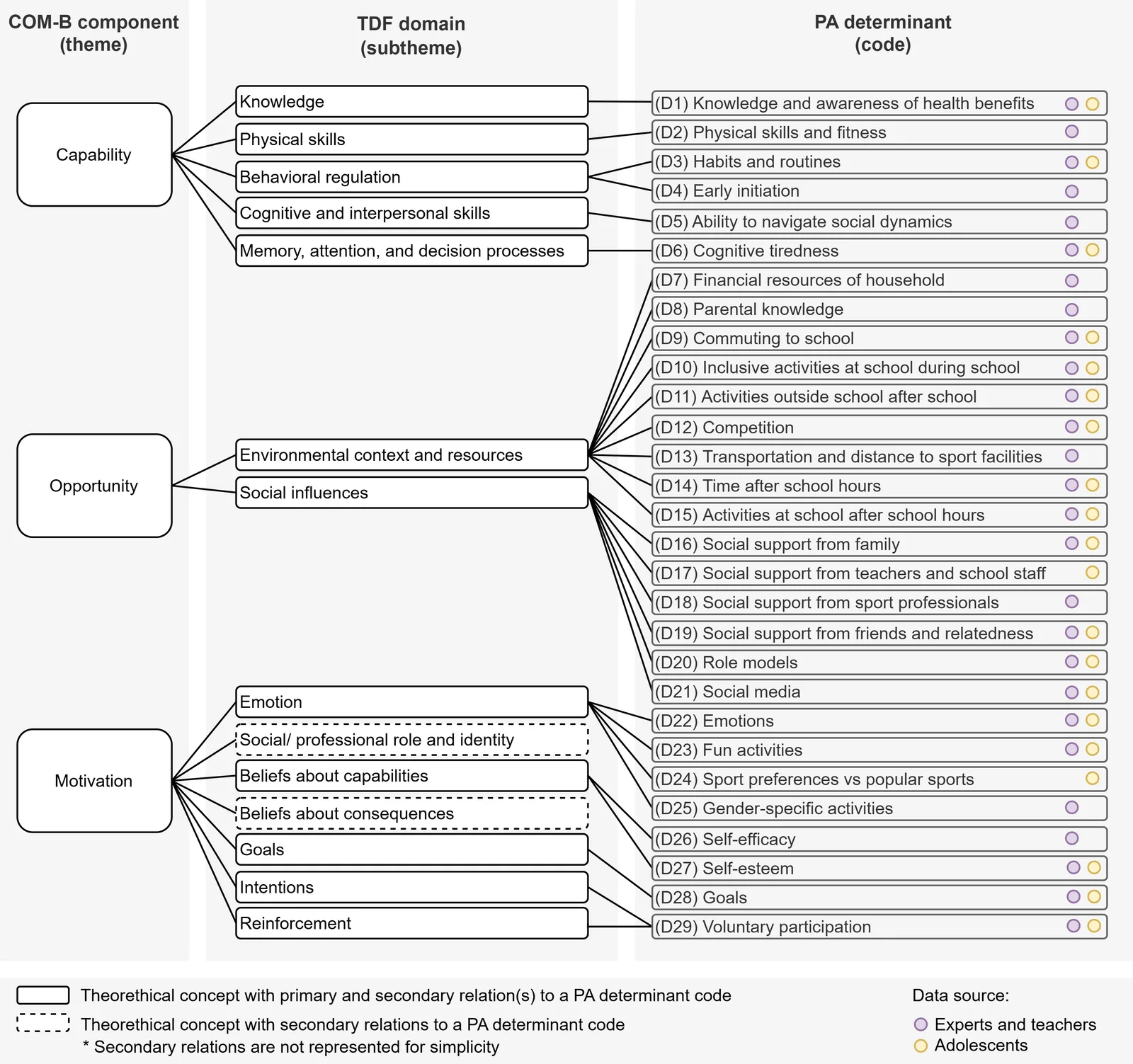
Thematic framework with COM-B components (themes), Theoretical Domains Framework (TDF) domains (subthemes) and physical activity (PA) determinants (codes). COM-B: Capability, Opportunity, Motivation-Behavior model; PA: physical activity; TDF: Theoretical Domains Framework.

Overall, PA determinants identified in all data sources were distributed across COM-B components as follows: opportunity (n=15), motivation (n=8), and capability (n=6). Comparing preconceptions from experts and teachers and perceptions from adolescents revealed both convergence and divergence across PA determinants. Experts and teachers emphasized capability and motivation barriers, prominently featuring the TDF domains of skills, reinforcement, and goals. These domains closely aligned with the impairments characteristic of mild intellectual disability. Conversely, adolescents consistently highlighted motivation and opportunity facilitators, particularly within the TDF domains of emotions and social influences.

Below, the PA determinants are described and organized based on the COM-B components and TDF domains, comparing experts’ and teachers’ preconceptions with adolescents’ perceptions.

#### Capability

##### Knowledge

Experts and teachers consistently emphasized adolescents’ lack of knowledge and awareness of the health benefits of PA (determinant 1, D1). Particularly, stakeholders noted that adolescents often find it difficult to comprehend the long-term effects of unhealthy behaviors and link PA with health outcomes beyond immediate effects. For example:

***Expert 1:** When they drink a liter of Coca-cola, they [the adolescents] don’t understand what it means for their body.*

In agreement, adolescents themselves often viewed PA in narrow and sport-related terms and were surprised that daily activities, such as cleaning and walking, also qualify as PA. Adolescents expressed orally, in postcards, and in timelines that “exercising is healthy,” but could not explain why beyond mentioning tangible effects, such as stronger muscles.

Experts also observed that adolescents may not know how to access structured PA opportunities, such as local sports clubs or school programs. They emphasized that social support is key to connecting these adolescents with existing programs or facilities that are unknown to them:

***Expert 10:** (...) take them by the hand and show them how nice it [PA opportunity] is.*

##### Skills and Behavior Regulation

Experts and teachers linked the adolescents’ impaired physical skills and fitness (D2) to their lack of active habits (D3) and to a late start in sport participation (D4). For example:

***Expert 5:** They are not used to engaging in sports already from a young age. Then it continues to be harder for them because they are not used to throwing and catching the ball.*

According to experts and teachers, these adolescents find it challenging to break sedentary habits due to low skills and difficulties with action planning. They indicated that greater repetition and structured activities could facilitate skill development and habit formation. In alignment, many adolescents mentioned participating in sports on fixed days of the week, indicating that PA is embedded in regular and structured routines.

In addition, experts and teachers expressed adolescents’ impairments in cognitive, interpersonal, and adaptive behavior skills as barriers to participating in the social dynamics in sport activities (D5). These stakeholders mentioned that adolescents often need support to understand complex game rules, tactics, and instructions, communicate, and adapt to social cues and group dynamics. Such struggles were linked to the social and cognitive demands of PA environments (eg, sport clubs). Experts indicated that inclusive activities and safe social environments supported by staff with special needs training may support skill development.

***Expert 8:** If you want to integrate social learning within physical education, then you need a safe sports climate.*

Adolescents themselves did neither mention that they experience impairments in skills nor that such impairments would hinder their participation in sport and exercise.

##### Memory, Attention, and Decision Process

Cognitive tiredness (D6) with a limited attention span were identified by experts and teachers as significant barriers to PA, especially later in the day. Experts and teachers noted that adolescents find focusing on complex tasks for prolonged periods difficult.

***Teacher 7:** They get tired. (...) rather tired or dropped out.*

Many adolescents used the “chillen” (chilling in Dutch) card at home in the evening on their timeline boards representing activities throughout a typical school day (Figure 5). The recurrent placement of “chillen” after school indicates a preference for sedentary evening activities. While adolescents did not explicitly link this behavior to tiredness, this pattern aligns with experts’ and teachers’ perceptions that cognitive fatigue may act as a barrier to PA during the evening after school.

**Figure 5.**
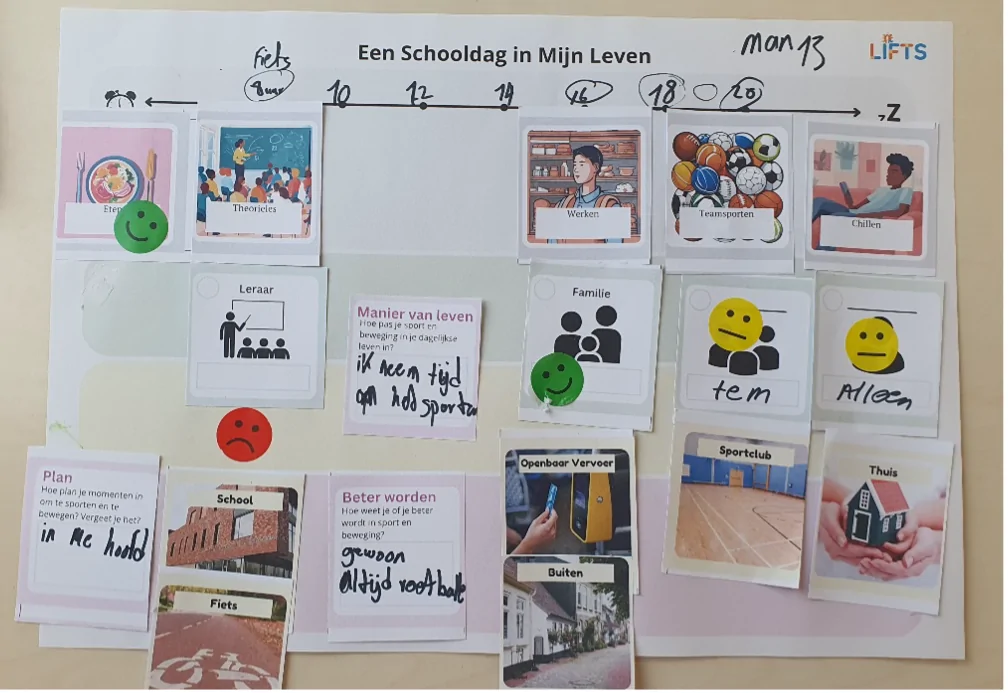
Timeline board created by an adolescent, showing activities throughout a typical school day. The “chillen” (chilling in Dutch) card positioned at home in the evening (top-right corner) indicates habitual sedentary time. Smiley face stickers indicate emotional valence of social influences.

### Opportunity

#### Environmental Context and Resources

Three main contexts emerged from participants’ input: home, school, and external environments, as described below.

Regarding the home, experts and teachers mentioned that financial difficulties (D7) and lack of parental knowledge (D8) often restrict adolescents from participating in organized PA opportunities. For example:

***Expert 5:** Their [ adolescents’ parents] primary focus is on getting up in the morning, getting food on the table, work, (...) so getting an income and then sports and physical activity is, yeah, not a priority for most.*

The adolescents rarely mentioned financial barriers, and only did it in anonymous formats (eg, postcards). Experts and teachers identified local and governmental programs, such as subsidies and information dissemination, as facilitators for accessing PA opportunities. Next, they noted that the home environment lacks the social support adolescents need for PA, making the home environment unsuitable for individual and home-based formats such as online workouts. Consistently, adolescents themselves did not mention the home environment in their timeline boards as a setting where they currently engage in PA.

Regarding the school, experts and teachers consistently appointed the school as a key environment for inclusive and tailored PA opportunities. Facilitators included active commutes to school (D9), inclusive physical education (PE) curricula, and additional organized (eg, class tournaments between classes and regular walk programs) or nudged (eg, available equipment and facilities during the breaks) activities at school (D10). Teachers added that PrO classes encourage natural movement throughout the day, which may lead to tiredness affecting students’ capability and motivation to engage in PA after school. Despite these opportunities, schools often lacked the staff and resources needed to sustain PA initiatives. Stronger school health policies and government support were identified as facilitators to institutionalize PA efforts. Consistently, many adolescents identified PA related to school on their timeline boards via active commutes, PE classes, and breaks. However, many did not mark the school period as active in the timeline (Figure 5) and thus viewed the school environment as sedentary. Older adolescents reported that PE classes were removed from their curriculum, reducing their chances for structured PA at school. Organized or nudged activities were rarely mentioned, and the PrO classes were not recognized as active.

Regarding the external environment, experts and teachers indicated that the external environment, outside the home and school contexts, is less inclusive than the PrO school environment, which caters to adolescents’ needs. These adolescents often struggle to integrate into local sport clubs and neighborhood activities (D11), where the setting is often performance-oriented and competition*-*driven (D12). Their skill impairments seem to lead to a mismatch and early dropout, as many fail to perform at the same level and feel isolated. It seems that, outside of school, adolescents face additional logistical barriers, such as transportation and distance to sport facilities (D13).

***Teacher 1:** They experience quite a big barrier to start with sports because of the distance, because they have to arrange getting to a Sports Club.*

While experts and teachers emphasized the need for dedicated, inclusive, and local sport programs, they noted that such programs often lack sufficient funding, governmental commitment, and a systems-thinking approach to social and environmental factors. These external challenges were reflected in adolescents’ input. While many mentioned participation in sport clubs, others reported engaging in individual and flexible sports such as kickboxing, swimming, fitness, and spontaneous PA outside after school hours among peers. These opportunities seem to emphasize participation over competition (D12), making them more inclusive.

Across environmental contexts, participants described time-related determinants. Experts and teachers viewed structured PA during school hours (D10) as a facilitator and suggested embedding PA into the school day (eg, active breaks or movement-based educational tools). In contrast, PA after school hours (D14) was often constrained because experts and teachers mentioned competing priorities, such as rest and responsibilities such as part-time jobs or household chores. Similarly, adolescents’ timeline boards showed that many preferred resting, and some had part-time jobs after school, reflecting these competing demands later in the day. To address this, experts proposed low-threshold PA opportunities immediately after the last school hour, leveraging the school setting after school hours (D15) while motivation and structure remain. Additionally, commercial gyms and other flexible options were recommended to fit adolescents’ diverse schedules and responsibilities.

#### Social Influences

Experts and teachers consistently emphasized that social influences from family, peers, and professionals are key PA determinants among these adolescents. Preconceived barriers related to family social support (D16) included financial stresses, unstable households, lack of parental knowledge, inability to provide practical aspects (eg, transportation), social segregation, and overprotection of adolescents.

***Expert 10:** It’s financial, it’s social, it’s psychological... there are often a lot of more important, more urgent issues, rather than getting your kid to the Sports Club.*

Regarding social support from school teachers (D17) and PA professionals (eg, coaches and neighborhood coaches) (D18), the focus on performance, competition, and lack of training on special needs were indicated as barriers. In contrast, child-centered coaching, inclusive activities in a safe environment, and specialized professionals were highlighted as facilitators. Even though it was indicated that these adolescents struggle to form social connections and networks, support and joint participation with friends and peers (D19) was mentioned as a significant facilitator for enjoying PA by promoting a sense of belonging through connection and teamwork.

***Expert 3:** They do it because it’s fun and because the friends are participating. That’s a drive.*

These social circles also influence PA participation through role modeling and group norms, where “seeing movement is moving.” Experts and teachers mentioned that the lack of active role models (D20) is a significant barrier to participation. Moreover, in many social circles (eg, groups of friends and neighborhood), a sedentary lifestyle is the norm, and PA may even be discouraged. Conversely, when being active is the norm, it can strongly promote PA:

***Expert 5:** If the norm in the friend group is we like sports, then everyone is like sports and there cannot be exceptions.*

Visible role models and social media influencers (D21) were mentioned as potential facilitators by experts and teachers.

Despite the stakeholders’ emphasis on social barriers, adolescents generally seemed positive about social support and occasionally expressed a desire for competition (D12). Adolescents often mentioned family in their postcards and timeline boards as motivating and supportive for PA. Figure 6 gives 2 examples of postcards illustrating common themes of social connection and enjoyment. The desire to participate in PA with friends was a recurring theme across data collection activities. When asked why they think PA is fun, they often replied, “Het is gezellig met vrienden” (a common Dutch expression about social connectedness).

**Figure 6.**
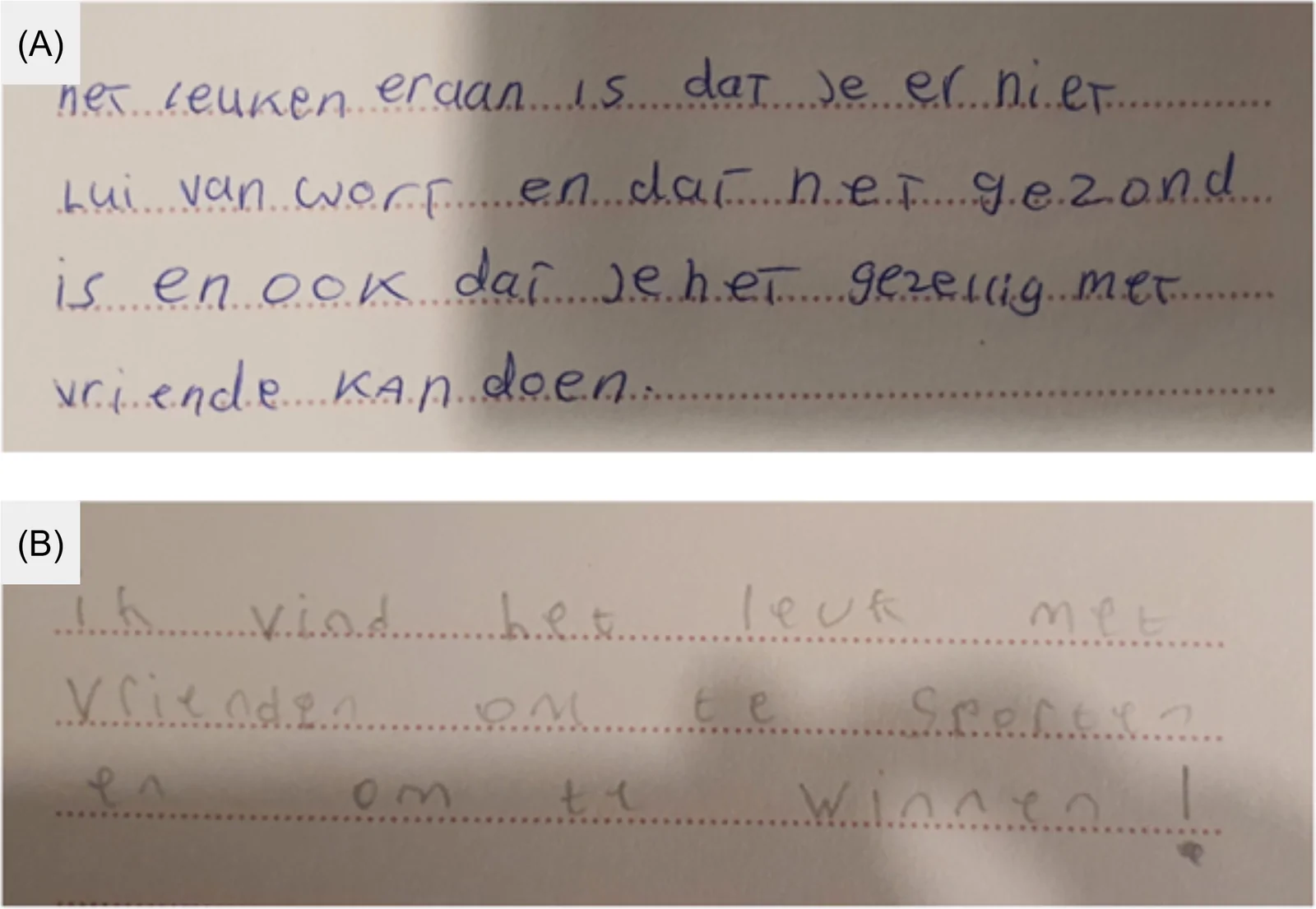
Two representative postcards from the sensitizing phase responding to the question “What do you think is fun about sports and exercise?” Translation: (A) "The nice thing about it is that you don’t get lazy and it’s healthy and you can do it in a cozy way with friends.” (B) “I like to play sports with friends and win!”

However, this positive attitude was not universal. Many adolescents perceived their teachers as unsupportive and sedentary role models. In postcards, only a few adolescents spontaneously mentioned their coaches or PE teachers as people who make them enthusiastic about PA. Importantly, some adolescents reported limited social support or that nobody makes them enthusiastic about PA. The timeline boards revealed that many students spend a lot of time alone at home after school, indicating a lack of social support, connectedness, and network.

### Motivation

#### Emotions and Social Identity

Experts and teachers highlighted positive emotions (D22) as a central motivator for PA, both in terms of experienced and anticipated feelings. Enjoyment and having fun (D23) were consistently noted as key facilitators of PA. This was linked to participation in preferred PA options, which can be related to gender preferences and segregation (D25). In agreement, these PA determinants were reported by the adolescents. Across data collection activities, adolescents consistently described sports and exercise as “fun.” An overall positive and energetic feeling was also expressed by many adolescents, with some highlighting that PA has mental health benefits (eg, feeling calm, occupied, and distracted from other issues). Often linked to the enjoyment of PA, it seems that when sports and movement are part of adolescents’ identity, they are more likely to participate in PA. Conversely, when adolescents identify with sedentary activities and not as “sportive” individuals, PA participation seems difficult. Similarly, personal sport preferences among adolescents seem to be a barrier if they diverge from the popular options (D24).

In addition, experts and teachers also identified negative emotions related to PA, including general tiredness, anxiety, and disappointment in competitive environments (D12), fear and shame of participating due to a perceived lack of skills, and disgust related to sweating. It seems that these adolescents with impaired adaptive behavior skills find it harder to regulate emotions than their typically developing peers. Negative emotions were less frequently reported by the adolescents themselves.

#### Beliefs About Capabilities and Consequences and Reinforcement

Experts and teachers consistently identified low self-efficacy (D26) and self-esteem (D27), particularly regarding body image, and overall capabilities, as barriers to PA participation. These preconceptions are linked to the adolescents’ skill impairments relative to their peers, and past negative experiences in performance-focused settings, including at other nontailored school settings. Experts and teachers emphasized that enabling small successes through scaffolding, safe environments, and positive reinforcement could facilitate skill development and increased self-efficacy. Experts and teachers added that enabling adolescents to participate in PA voluntarily is relevant, as opposed to a top-down enforcement that may have a negative impact. Conversely, adolescents did not explicitly report low self-efficacy, but some expressed concerns about body image, linking PA only to weight loss.

#### Goals and Intentions

Experts and teachers consistently noted that these adolescents often struggle to set long-term or abstract goals for PA (D28) due to their cognitive impairments. Instead, they mentioned that adolescents benefit from short-term, concrete goals that offer immediate outcomes, such as feeling stronger or more energetic. They emphasized that these goals should be voluntary (D29), aligning with the adolescent’s interests and emotional drivers.

***Expert 11:** Finding goals or targets that people have short-term benefit from, I feel stronger. I feel more energetic.*

Consistently, the adolescents did not explicitly express long-term goals related to PA in their postcards or timeline boards.

### Quantitative Findings

#### Descriptive Analysis

Table S1 in Multimedia Appendix 5 describes the adolescents’ ratings (from strong barriers to strong facilitators; 1 to 7) of the PA determinants identified in the qualitative phase from experts, teachers, and adolescents’ input. Ratings exhibit a positive trend (mean 5.09; SD 1.72), suggesting that the determinants are predominantly perceived by the adolescents as facilitators rather than barriers. Additionally, results show weak within-group agreement (r_wg_<0.7) across all determinants, indicating that there is a substantial variation in perceptions between adolescents.

#### Clustering Analysis

Figure 7 displays the hierarchical clustering of PA determinants, derived from the mean ratings and the within-group agreement indices (r_wg_). The variation in agreement appears to be partially explained by the mean rating of the determinants. A Spearman correlation analysis confirmed a significant positive relationship between mean rating and agreement (*ρ*=0.74, *P*<.001). This indicates that determinants that are more frequently perceived as facilitators tend to elicit greater consensus among adolescents’ perceptions.

**Figure 7.**
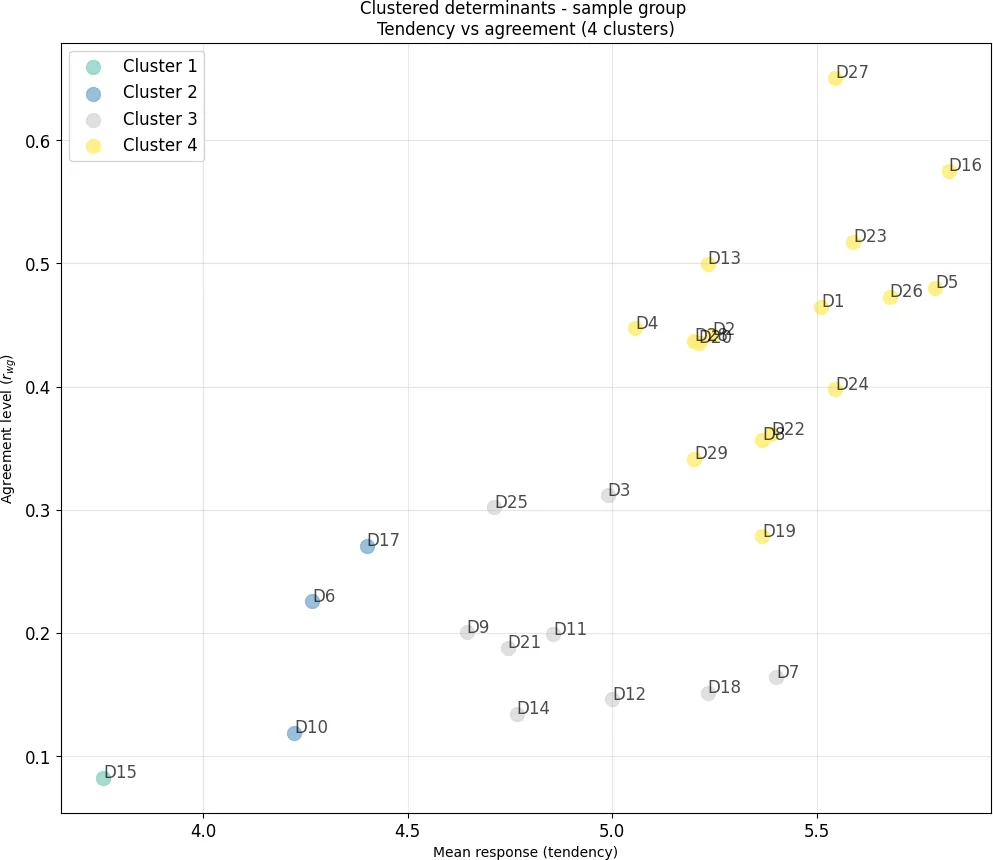
Clustering of physical activity determinants (D) based on adolescents’ overall mean rating and within-group agreement (r_wg_).

Table 3 shows 4 clusters of PA determinants from hierarchical clustering. One cluster is a singleton with the lowest agreement, while the remaining clusters indicate a good structure. We describe and interpret these clusters below.

**Table 3.** Clusters of PA^a^ determinants based on adolescents’ mean ratings, r_wg_^b^, and average silhouette test score, except for singleton cluster 1.

Cluster	Intuitive description	PA determinants	Mean (SD)	r_wg_	Average score (SD)
1	Nonconsensual and stronger barriers	D15: activities at school after school hours	3.76 (0.08)	0.08	N/A^c^
2	Less often perceived and mixed barriers	D6: cognitive tiredness (after school) D10: inclusive activities at school during school D17: social support from teachers and school staff	4.30 (0.08)	0.21	0.366 (0.212)
3	Less often perceived and weaker facilitators	D3: habits and routines D7: financial resources of household D9: commuting to school D11: activities outside school after school D12: competition D14: time after school hours D18: social support from sport professionals D21: social media D25: gender-specific activities	4.93 (0.24)	0.20	0.318 (0.175) (good)
4	Most often perceived and stronger facilitators	D1: knowledge and awareness of health benefits D2: physical skills and fitness D4: early initiation D5: ability to navigate social dynamics D8: parental knowledge D13: transportation and distance to sport facilities D16: social support from family D19: social support from friends and relatedness D20: role models D22: emotions D23: fun activities D24: sport preferences vs popular sports D26: self-efficacy D27: self-esteem D28: goals D29: voluntary participation	5.42 (0.22)	0.45	0.495 (0.189)

a PA: physical activity.

b r_wg_: within-group agreement.

c N/A: not applicable.

Clusters 1 and 2 represent determinants perceived as barriers to PA by adolescents with centroids below the overall mean rating and lower within-group agreement. Cluster 1 is a singleton of a determinant with the lowest agreement level. Both clusters encompass school-related barriers, including after-school activities at the school premises (D15), inclusive activities during regular school hours (D10), cognitive tiredness after school (D6), and perceived lack of support from school staff (D17).

Cluster 3 represents determinants that are less often perceived as facilitators to PA, including habits and routines (D3), financial resources of household (D7), commuting to school (D9), activities outside school after school (D11), competition (D12), transportation and distance to sport facilities (D13), social support from sport professionals (D18), social media (D21), and gender-specific activities (D25).

Cluster 4 contained the most often perceived facilitators to PA. This cluster includes expected determinants related to emotions (D22), fun activities (D23), popular vs personal sport preferences (D24), social support from family and friends (D19), positive role models (D20), and parental knowledge (D8). Additionally, this cluster also includes knowledge (D1), physical skills and fitness (D2), early initiation (D4), ability to navigate social dynamics in sports (D5), self-efficacy (D26), self-esteem (D27), goals (D28), and voluntary participation (D29).

### Interpretation of Adolescents’ Perceptions

Table S2 in Multimedia Appendix 5 presents the gamified quiz items and responses used to validate the researchers’ interpretation of adolescents’ perceived PA determinants. The quiz focused on determinants where adolescents’ ratings diverged from experts’ and teachers’ qualitative input. For example, adolescents often rated social support from family as a facilitator, whereas experts and teachers more often viewed it as a barrier. Adolescents’ quiz responses aligned with the clustering results, providing additional support for the identified patterns. One exception concerned after-school activities at school (D15), the determinant with the lowest within-group agreement (cluster 1). While adolescents’ ratings suggested weak within-group agreement, most adolescents reported it as a barrier (62/82, 76%, preferring rest or other sedentary activities; 66/85, 78%, wanting to go home). This indicates that this determinant may be more commonly experienced as a barrier.

For several determinants in cluster 4, quiz findings confirmed the convergence between part of adolescents’ perceptions and expert and teacher preconceptions. Social support from family (D16; 59/87, 68%, reporting positive encouragement) and friends (D19; 49/87, 56%, reporting support) was predominantly reported as supportive for PA participation. Similarly, determinants identified as capability barriers by experts and teachers were again not often perceived as barriers by adolescents, including self-efficacy (D26; 57/85, 67%, reporting positive beliefs about capabilities) and self-esteem (D27; 68/84, 81%, reporting a positive body image during sports).

## Discussion

### Identification of Determinants

The qualitative phase identified 29 PA determinants among adolescents with mild intellectual disability in PrO schools from multistakeholder perspectives. A key finding was that experts and teachers emphasized determinants rooted in capability impairments and opportunity constraints, such as limited skills, low self-efficacy, and insufficient inclusive sports opportunities. These findings reflect impairments of mild intellectual disability that adolescents seemed unaware of or reluctant to disclose, and that were more evident to teachers and experts because adult support may shield them in adolescents’ daily lives [3]. In contrast, adolescents themselves emphasized opportunity and motivation determinants and viewed PA mainly through a social and emotional lens. They focused, for example, on enjoyment, fun, being with friends, receiving family support, and doing preferred activities.

Overall, the determinants identified are consistent with previous studies among adolescents with mild intellectual disability [21,22,24-34] but also highlight differences across stakeholder perspectives. While prior studies focused primarily on adolescents’ views, our findings suggest that triangulating adolescent, teacher, and expert perspectives provides a more comprehensive understanding of the factors shaping PA participation.

### Prioritization of PA Determinants and Implications for Interventions

#### Overview

The quantitative phase examined how adolescents perceived the PA determinants identified across adolescents, teachers, and experts, prioritizing these from barriers to facilitators. Integrating the adolescents’ ratings with the qualitative findings generated 3 key meta-inferences for intervention adaptation.

#### Individual Variation in Adolescent Perceptions

The systematic weak within-group agreement of adolescents’ ratings indicates that they perceive PA determinants individually and differently, despite sharing the mild intellectual disability characteristics and a similar school environment. This finding aligns with behavior sciences frameworks emphasizing that behavior is shaped by its dynamic context and shows individual variability [17,74]. Variation was also evident in the determinants emphasized by each expert and teacher, while literature similarly shows inconsistencies in the determinants identified in comparable groups [19]. This evidence suggests that individual variation in perceived PA determinants among the adolescents is genuine rather than a research artifact.

A related finding was that determinants perceived as facilitators achieved relatively higher consensus, whereas barriers showed particularly weak within-group agreement. The integrated meta-inference is, therefore, that PA interventions should offer a dual strategy: personalized components to address highly individualized barriers and group-level components to enhance more shared facilitators. This duality seems overlooked in prior research, which has focused on the qualitative prevalence of themes in target groups [21,22,30-34] or on statistical comparisons between demographic groups rather than individual variation [24-29]. Modern digital health tools offer unprecedented opportunities to operationalize this dual approach, while being inclusive and scalable [11,75]. For example, cognitive tiredness after school, identified in this study as a barrier, manifests as habitual sedentary periods for some adolescents. Digital tools could identify these opportune moments to promote enjoyable or socially supported forms of PA.

#### Individual Barriers

Determinants that tended to be perceived as barriers by adolescents (clusters 1 and 2) showed weak agreement and primarily related to the school environment, including activities during or after school hours, cognitive tiredness after school, and limited support from school staff. The validation quiz reinforced this finding by showing that many adolescents preferred going home or resting rather than participating in PA at school after school hours. However, experts and teachers viewed the school environment as promising for inclusive PA opportunities. This is consistent with evidence of school-related determinants found in literature for adolescents with mild intellectual disability [21,24-26,28,31] and prior studies evaluating school-based interventions [76,77].

Integrating these findings, the key meta-inference is that, although schools are a promising setting for inclusive PA interventions, school-based PA opportunities may not align with every adolescent’s individual needs, lived experiences, and wishes. Therefore, interventions should combine the strengths of the school setting with tailored support for individual barriers, particularly focusing on unstructured time (eg, during breaks and after school hours) [78]. For example, one approach could be to use school-led digital interventions that extend beyond the school premises, enabling teachers and staff to support PA participation in settings such as the home or local parks when these better fit adolescents’ needs and daily lives.

#### Shared Facilitators

Adolescents rated some determinants as facilitators, aligning with qualitative input from experts and teachers, including emotions, fun activities, popular vs personal sport preferences, social support from family and friends, positive role models, and parental knowledge. These determinants were clustered among the highest-rated facilitators and demonstrated relatively greater consensus across adolescents (clusters 3 and 4). However, adolescents rated several determinants as facilitators that experts and teachers emphasized as barriers, particularly those related to impaired skills and self-efficacy. In addition to the qualitative phase explanations above, three other factors may account for this discrepancy in the quantitative phase. First, adolescents rated PA determinants more often as facilitators than barriers (mean 5.1/7, SD 1.72), suggesting that they answered based on what they believe matters for PA participation rather than their own experiences. For example, self-efficacy was often rated as a facilitator, indicating adolescents know it helps participation even if they do not feel confident themselves. Second, social desirability bias may have led adolescents to underreport personal struggles. Third, adolescents may genuinely view PA barriers and facilitators differently, emphasizing emotional and social aspects meaningful to them.

The integrated meta-inference is that PA interventions should prioritize shared facilitators as core group-level targets. In other words, interventions should target adolescents’ positive meaning of PA participation, such as enjoyment and social influences. At the same time, teacher and expert perspectives suggest that impairment-related determinants may still influence adolescents’ ability to benefit from the shared facilitators. Therefore, while interventions should avoid being driven solely by an impairment-based lens, such considerations remain important for ensuring that shared facilitators are accessible and meaningful for adolescents with mild intellectual disability.

### Comparison With Other Groups in Literature

#### Comparison With Typically Developing Peers

Research on typically developing adolescents has consistently shown that social influences and enjoyment are primary facilitators of PA participation [79]. This is consistent with our findings in adolescents with mild intellectual disability, suggesting that these facilitators are shared across adolescents’ populations regardless of cognitive ability. Nevertheless, experts and teachers in this study mentioned barriers specific to adolescents with mild intellectual disability, such as impaired capabilities, cognitive tiredness, struggles in competitive activities, and the need for structured support. These barriers underscore the need for tailored interventions in the PrO context, although adaptations may be less extensive than those needed for adolescents with more severe intellectual disability who experience greater barriers to PA participation [19,80,81].

#### Comparison With Low Socioeconomic Status

A larger proportion of adolescents attending PrO schools in the Netherlands comes from families with low socioeconomic status (SES) compared to other education tracks [82]. Previous research has identified barriers to PA among low-SES adolescents that are consistent with those identified by experts and teachers in this study, including limited household resources, low family support due to competing priorities, and difficulties accessing PA opportunities [83]. Similarly, social support from friends and enjoyment have been identified as facilitators across all SES groups, which is consistent with our findings [83]. This suggests that barriers may be more dependent on individual circumstances, including SES, while facilitators such as enjoyment and social support appear to be shared across adolescents.

### Contribution, Limitations, and Future Work

This formative research addressed a key knowledge gap regarding PA determinants among adolescents with mild intellectual disability in Dutch PrO schools by integrating 3 complementary methodological lenses: theoretical grounding, inclusive participatory methods, and multistakeholder triangulation. It was designed to generate context-specific knowledge for intervention adaptation in Dutch PrO schools rather than generalizable estimates across all PrO schools and other contexts. Consistent with this aim, we prioritized information-rich perspectives over representativeness and achieved meaning saturation within the participating schools. Accordingly, our main contribution lies in this study’s contextual relevance for intervention design. At the same time, the determinants identified were largely consistent with findings previously reported among adolescents with mild intellectual disability and among adolescents from low-SES backgrounds, suggesting that our findings are unlikely to be unique to the 2 participating PrO schools.

Several limitations can be considered. First, despite efforts to design inclusive participatory methods, adolescents often struggled to participate. In the qualitative phase, they faced challenges articulating their experiences when asked open-ended questions, responding more effectively when prompted with creative methods to share concrete personal stories in conversations [3,84]. This highlights the importance of nonabstract and visually supported methods in inclusive participatory research [21,37,85,86].

Second, because adolescents’ perspectives were collected using creative and visual activities that were not audio-recorded to facilitate their participation and comfort, their viewpoints were collected differently than those of experts and teachers, whose perspectives were retrieved via interviews and focus groups and were audio-recorded and transcribed verbatim. This difference in method and data format (postcards, collages, and field notes vs verbatim transcripts) may have introduced an analytical imbalance and could have increased the relative prominence of adult stakeholder perspectives.

Third, in the quantitative phase, the Likert-scale ratings may have reflected the perceived importance of PA determinants rather than the actual barriers and facilitators adolescents encounter in daily life. This suggests that Likert-scales may require additional clarification to be reliable for adolescents with mild intellectual disability [67]. In both phases, social desirability may have influenced responses [87]. Although activities were designed for individual, anonymous completion, the group setting let adolescents observe and discuss each other’s choices, which may have led to underreporting of personal barriers.

Finally, objective PA measures (eg, accelerometers and pedometers) were not included as another data source for triangulation. This limits the ability to validate perceived determinants against actual behavior, which could have helped contextualize the optimistic attitudes expressed by adolescents and enhance ecological validity.

Despite these limitations, the findings of this formative study offer meta-inferences for adapting digital health interventions promoting PA among adolescents with mild intellectual disability in the PrO context. Future research can build on these findings by using frameworks such as the Behavior Change Wheel to translate prioritized determinants into intervention functions, behavior change techniques, and digital intervention components tailored to this population and context [17].

An important finding warranting further investigation is the substantial individual variation in how the adolescents perceived PA determinants as barriers or facilitators. Such variability signals the need to recognize behavioral heterogeneity even within demographically similar populations and contexts [88,89]. Future research should explore how health behavior change research methods can better account for individual variability in what influences behavior and how such variability can inform personalized interventions. Embracing individual-level behavioral assessments and adaptive, scalable digital interventions may offer promising ways to operationalize this shift.

### Conclusions

This formative research identified and prioritized PA determinants among adolescents with mild intellectual disability in PrO schools in the Netherlands through the integration of theory-driven, inclusive, multistakeholder participatory lenses. The findings suggest that PA intervention adaptation should move beyond “one size fits all” approaches by recognizing both the facilitators that are more shared among adolescents, including enjoyment and social support, and those that are experienced more individually, including school-related barriers. Future research should build on these prioritized determinants to identify intervention components that can operationalize this dual approach. To do so, digital technologies are uniquely positioned to combine scalable support for shared facilitators (eg, components that promote enjoyment and social connectedness among peers) with personalized support to reduce individual barriers (eg, tailoring support to adolescents’ preferences, routines, and moments of low energy after school), thereby improving contextual fit for adolescents with mild intellectual disability in the PrO setting.

## Supplementary material

10.2196/89518 Multimedia Appendix 1 Posters for teacher focus groups and asynchronous activity.

10.2196/89518 Multimedia Appendix 2 Materials used in the sensitizing phase by adolescents.

10.2196/89518 Multimedia Appendix 3 Materials used in the generation phase by adolescents.

10.2196/89518 Multimedia Appendix 4 Materials used in the structuring phase by adolescents.

10.2196/89518 Multimedia Appendix 5 Quantitative results and analysis of ratings of physical activity determinants and responses to the validation quiz by adolescents.

10.2196/89518 Multimedia Appendix 6 Detailed maps of physical activity determinants derived from all qualitative sources, including literature, expert interviews, teacher input, and adolescent activities.

10.2196/89518 Checklist 1 Reporting on the COREQ checklist.

10.2196/89518 Checklist 2 GRAMMS checklist.

## Abbreviations

COM-B: Capability, Opportunity, Motivation-Behavior
COREQ: Consolidated Criteria for Reporting Qualitative Research
GRAMMS: Good Reporting of a Mixed Methods Study
LIFTS: Healthy Lifestyle for Low Literate Teenagers
PA: physical activity
PE: physical education
PrO: Praktijkonderwijs (Dutch practical education)
SES: socioeconomic status
TDF: Theoretical Domains Framework
