# Supplementary material for: Physical Activity Determinants Among Adolescents With Mild Intellectual Disability in Dutch Practical Education: Exploratory Sequential Mixed Methods Study
Source: JMIR Form Res. 2026 Sep 30;10:e89518. doi: 10.2196/89518 (PMC13626643; doi:10.2196/89518)
Supplement: Supplementary file 1 [file formative-v10-e89518-s001.pdf]

LIFTS

Het **LIFTS-project** promoot een duurzaam gezond leven onder PrO-leerlingen, met focus op voeding, mentale gezondheid en fysieke activiteit. We betrekken leerlingen en hun omgeving om hun behoeften en ervaringen te begrijpen. Wat helpt hen een gezonde levensstijl aan te nemen? Samen ontwikkelen we toegankelijke, boeiende technologie die dit ondersteunt.

Vertel ons wat het moeilijker of makkelijker maakt voor leerlingen om te sporten en te bewegen.

1. **Schrijf je naam op de lijst.**
   We gebruiken deze lijst om het aantal mensen te tellen dat aan ons onderzoek bijdraagt. We registreren je naam niet. Deze lijst wordt vernietigd.

2. **Loop door de ruimte en bekijk de 4 grote posters.**
   Elke poster gaat over één niveau van factoren die ervoor zorgen dat leerlingen meer of minder sporten en bewegen. Bij elke poster vind je dit soort vragen om je brainstorm een kickstart te geven.

3. **Geef ons jouw input.**
   Gebruik groene post-its voor factoren die het leerlingen gemakkelijk maken om actief te zijn, zoals een ondersteunende ouder. Gebruik roze post-its voor factoren die het moeilijk maken, zoals het ontbreken van de juiste sportuitrusting.
   Je kunt alleen trefwoorden met een korte beschrijving aanpassen, of kleine verhalen of situaties delen.

Jouw inzichten zijn heel erg waardevol :)

Heel erg bedankt!

SOCIAL CONTEXT

Factoren die het Fysieke Activiteit van Leerlingen Beïnvloeden

Wat maakt het **moeilijker** en **makkelijker** voor leerlingen om te sporten en bewegen?

Hoe maken vrienden het makkelijker of moeilijker voor leerlingen om te sporten en bewegen?

Hoe maakt familie het makkelijker of moeilijker voor leerlingen om te sporten en bewegen?

Hoe maakt school het makkelijker of moeilijker voor leerlingen om te sporten en bewegen?

Wie maakt het nog meer makkelijker of moeilijker voor leerlingen om te sporten en bewegen?

Wie is een rolmodel voor leerlingen om te sporten en bewegen? Wie is een slecht voorbeeld?

LIFTS

OMGEVING

Factoren die het Fysieke Activiteit van Leerlingen Beïnvloeden

Wat maakt het **moeilijker** en **makkelijker** voor leerlingen om te sporten en bewegen?

Waar sporten of bewegen leerlingen?

Welke voorwerpen gebruiken leerlingen bij het bewegen? Welke voorwerpen missen zij nog?

Hoeveel tijd besteden leerlingen ongeveer aan sport en bewegen?

Welke andere activiteiten moeten leerlingen doen of willen leerlingen doen dat ze belemmert om te gaan sporten en bewegen?

LIFTS

VAARDIGHEDEN

Factoren die het Fysieke Activiteit van Leerlingen Beïnvloeden

Wat maakt het **moeilijker** en **makkelijker** voor leerlingen om te sporten en bewegen?

Wat weten leerlingen wel of niet van het belang van sport en bewegen?

Wat weten leerlingen wel of niet over sporten en bewegen?

Welke vaardigheden hebben leerlingen om te sporten en bewegen? Welke vaardigheden missen ze nog?

Hoe passen leerlingen sporten en bewegen in hun dagelijks leven?

Hoe monitoren leerlingen hun vooruitgang in sport en bewegen?

MOTIVATIE

Factoren die het Fysieke Activiteit van Leerlingen Beïnvloeden

Wat maakt het **moeilijker** en **makkelijker** voor leerlingen om te sporten en bewegen?

Wat stimuleert leerlingen wel of niet om te sporten en bewegen?

Hoe voelen leerlingen zich tijdens het sporten en bewegen?

Hoe beïnvloeden gevoelens en emoties het sporten en bewegen van leerlingen?

Hoe past sporten en bewegen bij het zelfbeeld van leerlingen?

Wat denken leerlingen dat er gebeurt als zij sporten en bewegen?

Hoe graag sporten en bewegen leerlingen?

Plannen leerlingen sport- en beweegmomenten? Zijn er activiteiten die ze liever doen waardoor ze niet sporten of bewegen? Zijn er activiteiten die ze moeten doen waardoor ze niet sporten of bewegen?

LIFTS
