# Supplementary material for: Physical Activity Determinants Among Adolescents With Mild Intellectual Disability in Dutch Practical Education: Exploratory Sequential Mixed Methods Study
Source: JMIR Form Res. 2026 Sep 30;10:e89518. doi: 10.2196/89518 (PMC13626643; doi:10.2196/89518)
Supplement: Supplementary file 2 [file formative-v10-e89518-s002.pdf]

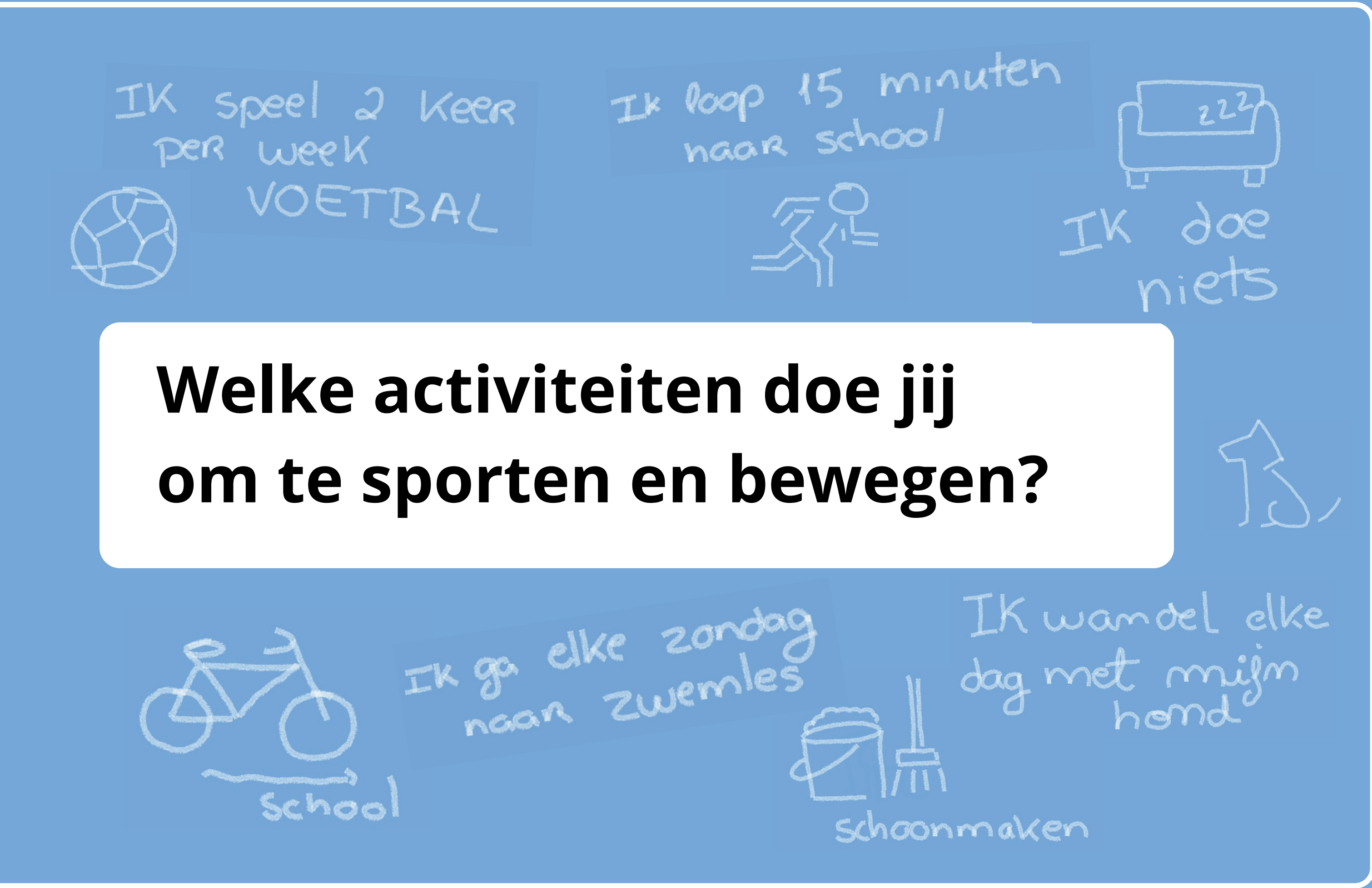

Welke activiteiten doe jij om te sporten en bewegen?

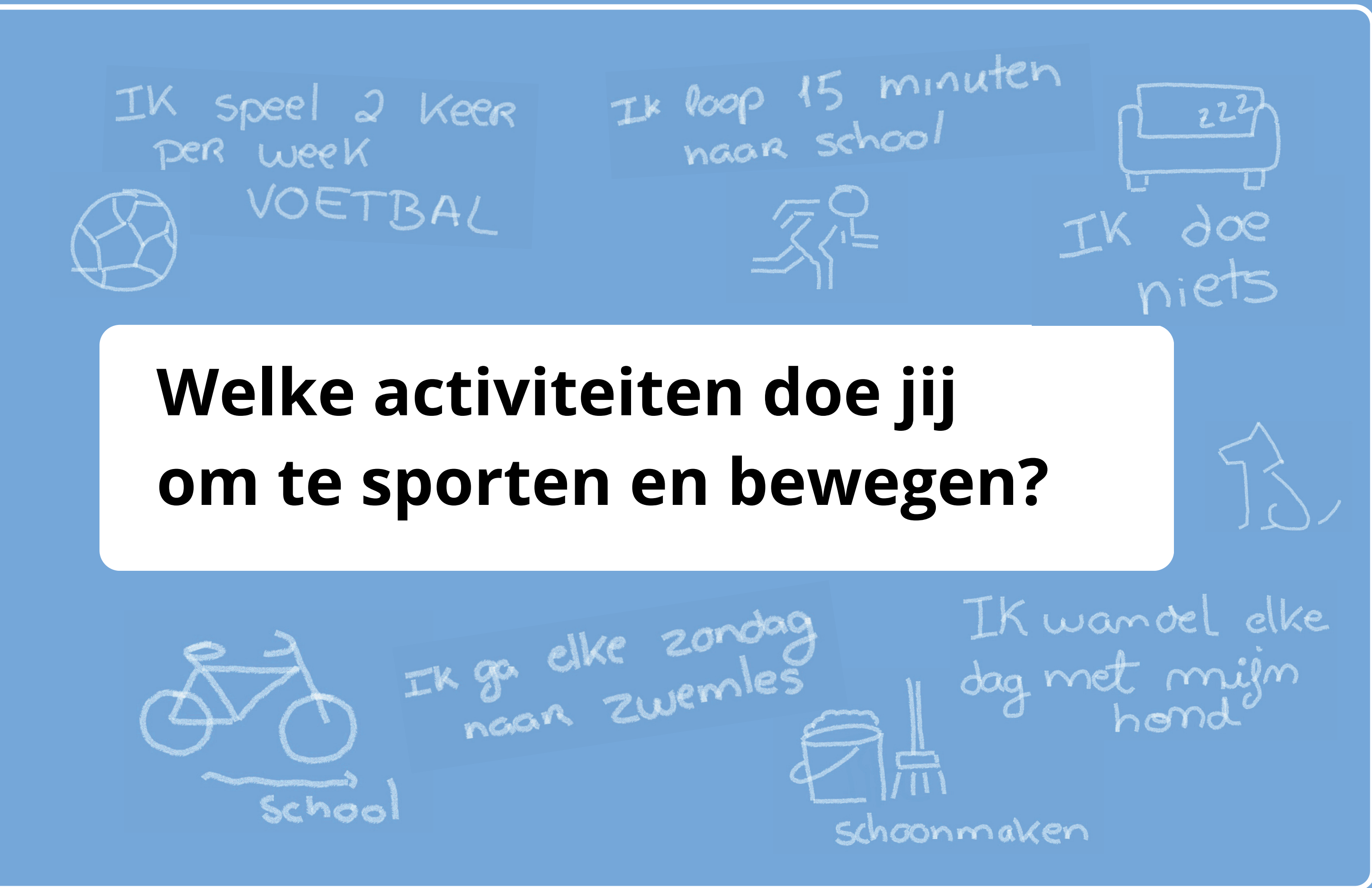

Welke activiteiten doe jij om te sporten en bewegen?

LIFTS

LIFTS

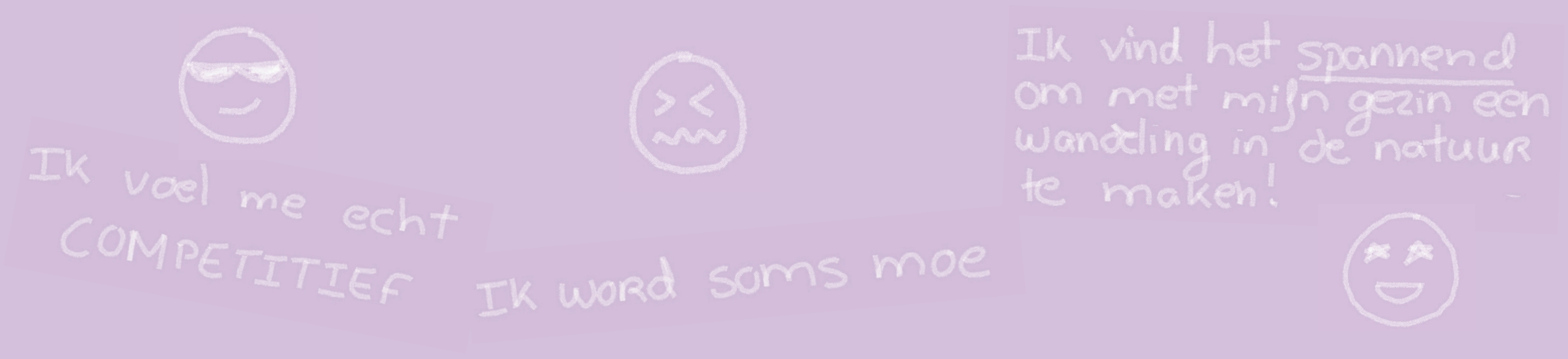

Hoe voel jij je tijdens en na sporten en bewegen?

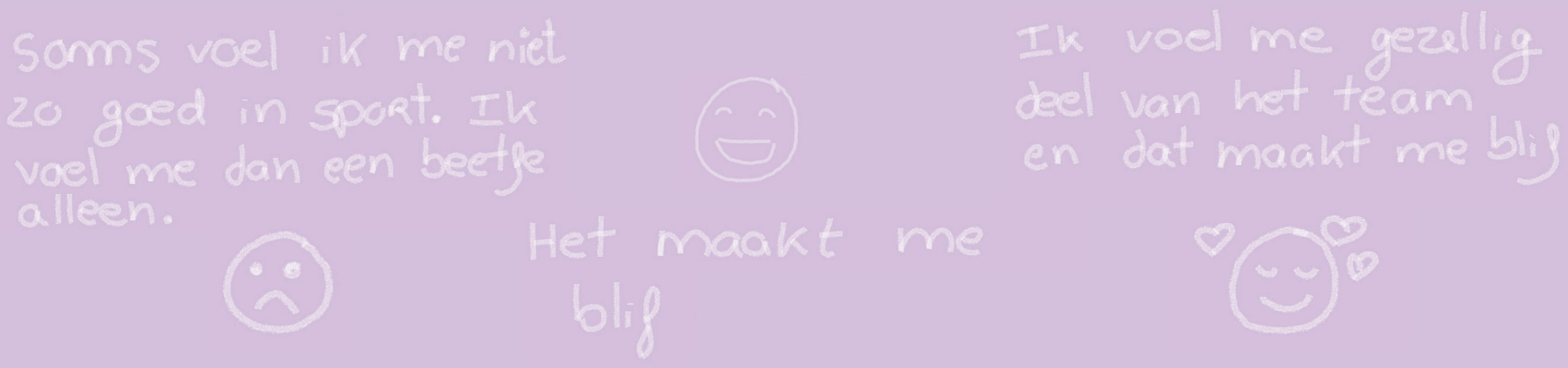

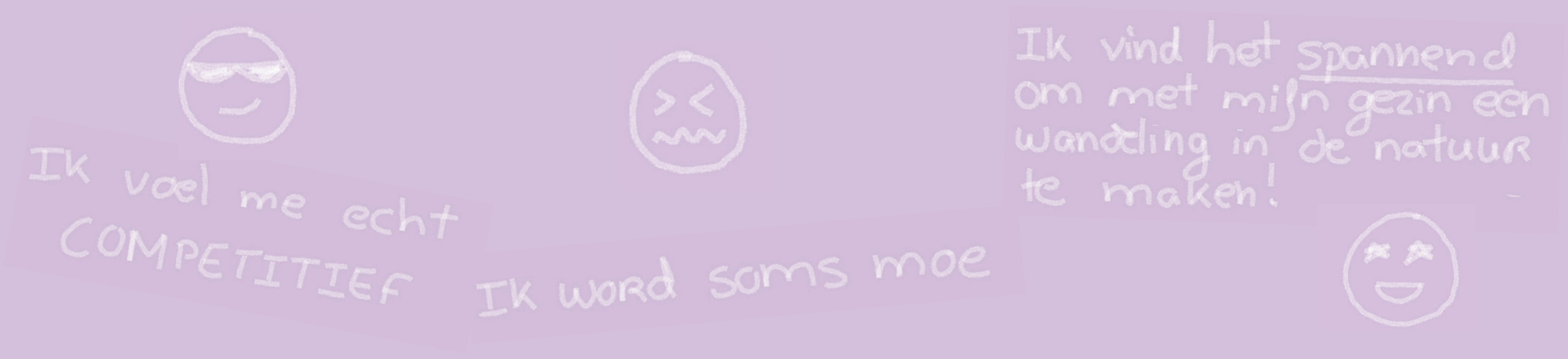

Hoe voel jij je tijdens en na sporten en bewegen?

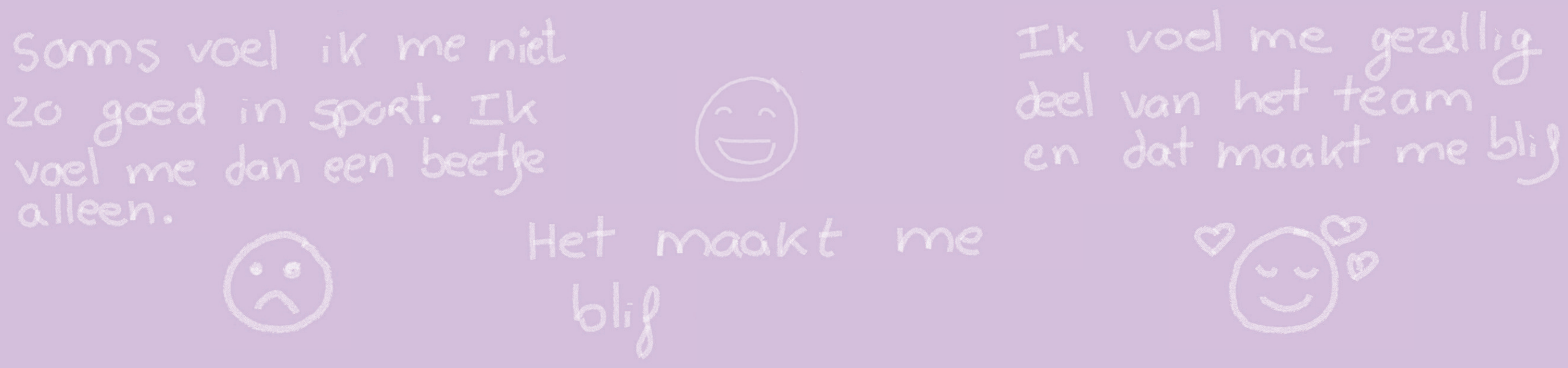

LIFTS

LIFTS

Bewegen helpt me om te ontspannen en mijn gedachten te verzetten.

Winnen → leuk
verliezen → frustrerend

Ik vind het geweldig als ik iets nieuws kan doen, yey!

Wat vind je leuk aan sporten en bewegen?

Het is leuk om samen met mijn vrienden te sporten en tijd door te brengen.

Ik houd van de uitdaging

Ik houd van de frisse lucht en de buitenlucht tijdens een wandeling

Bewegen helpt me om te ontspannen en mijn gedachten te verzetten.

Winnen → leuk
verliezen → frustrerend

Ik vind het geweldig als ik iets nieuws kan doen, yey!

Wat vind je leuk aan sporten en bewegen?

Het is leuk om samen met mijn vrienden te sporten en tijd door te brengen.

Ik houd van de uitdaging

Ik houd van de frisse lucht en de buitenlucht tijdens een wandeling

LIFTS

LIFTS

Mijn vrienden bij de sportclub
omdat we samen beter worden.

Lionel Messi
omdat zijn balcontrole bijna magisch is, en ik dat ook wil kunnen.

Wie maakt jou enthousiast om te sporten en bewegen?

Mijn ouders, omdat ze zelf altijd actief zijn!

Max Verstappen
Hij is zo snel en vastberanden. Ik wil net zo gefocust zijn in alles wat ik doe!

Mijn vrienden bij de sportclub
omdat we samen beter worden.

Lionel Messi
omdat zijn balcontrole bijna magisch is, en ik dat ook wil kunnen.

Wie maakt jou enthousiast om te sporten en bewegen?

Mijn ouders, omdat ze zelf altijd actief zijn!

Max Verstappen
Hij is zo snel en vastberanden. Ik wil net zo gefocust zijn in alles wat ik doe!

LIFTS

LIFTS

Meer vrije tijd na school

Nieuwe DOELEN

Betere schoenen

Een veilige plek

Iemand om mee te sporten

Meer leuke activiteiten op school

Waardoor ga jij meer sporten en bewegen?

De basis leren

Toegang tot sportplekjes

Een sport die je thuis kan doen

Activiteiten tijdens de schoolpauzes

Sportcoach

Iemand die me motiveert

Meer vrije tijd na school

Nieuwe DOELEN

Betere schoenen

Een veilige plek

Iemand om mee te sporten

Meer leuke activiteiten op school

Waardoor ga jij meer sporten en bewegen?

De basis leren

Toegang tot sportplekjes

Een sport die je thuis kan doen

Activiteiten tijdens de schoolpauzes

Sportcoach

Iemand die me motiveert

LIFTS

LIFTS
