# Supplementary material for: Physical Activity Determinants Among Adolescents With Mild Intellectual Disability in Dutch Practical Education: Exploratory Sequential Mixed Methods Study
Source: JMIR Form Res. 2026 Sep 30;10:e89518. doi: 10.2196/89518 (PMC13626643; doi:10.2196/89518)
Supplement: Supplementary file 3 [file formative-v10-e89518-s003.pdf]

# Een Schooldag in Mijn Leven

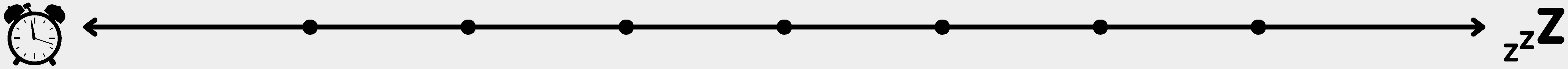

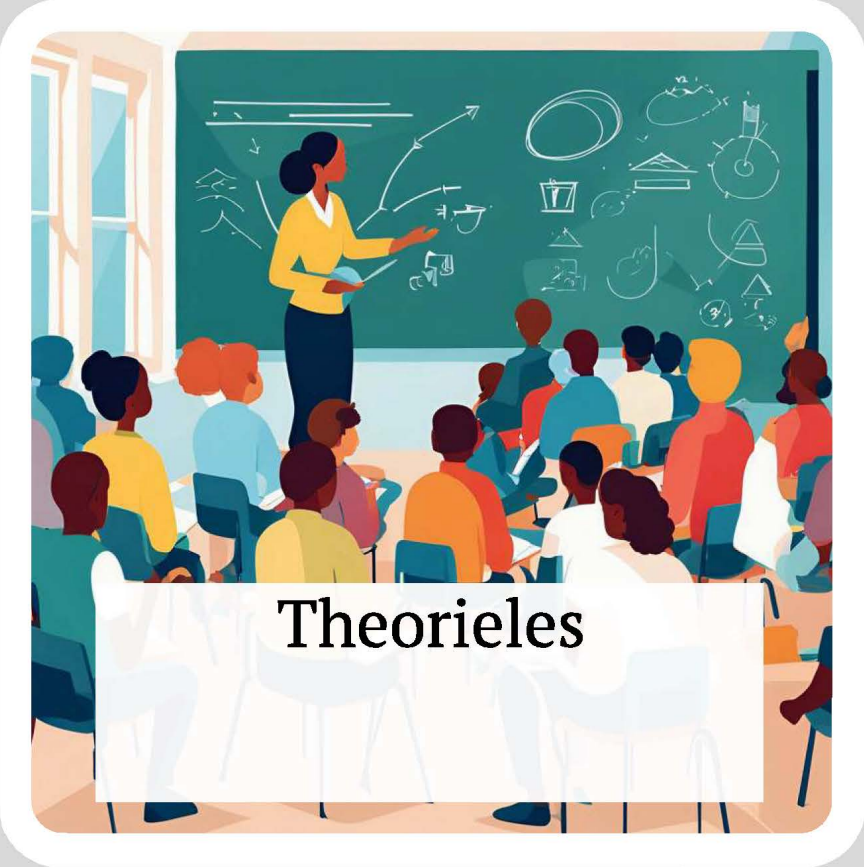
Theorieles

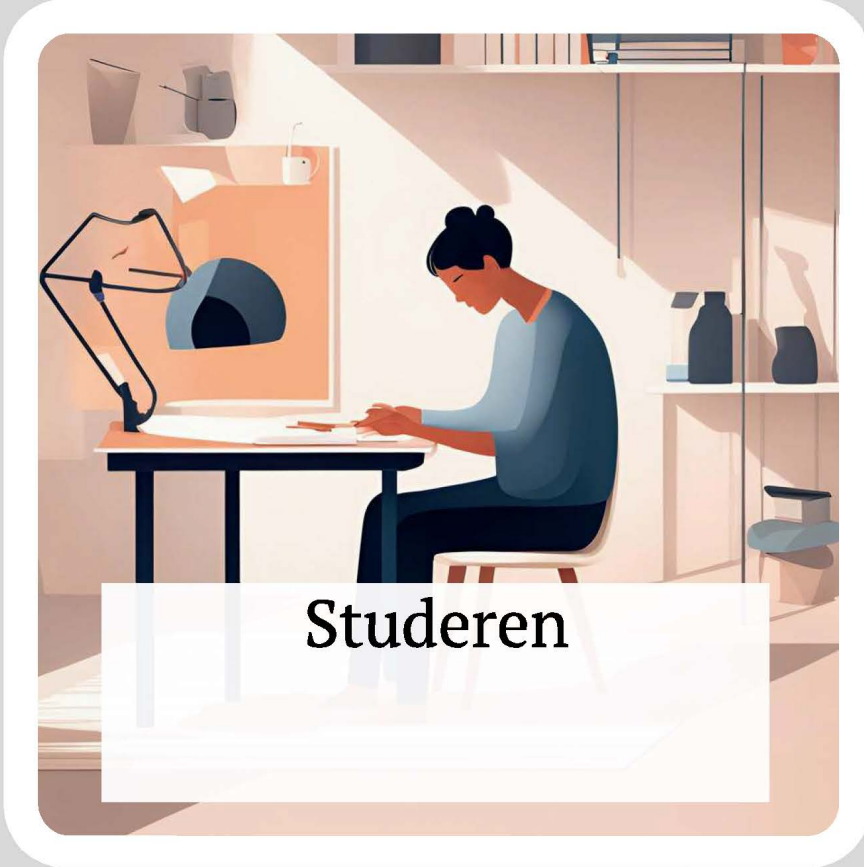
Studeren

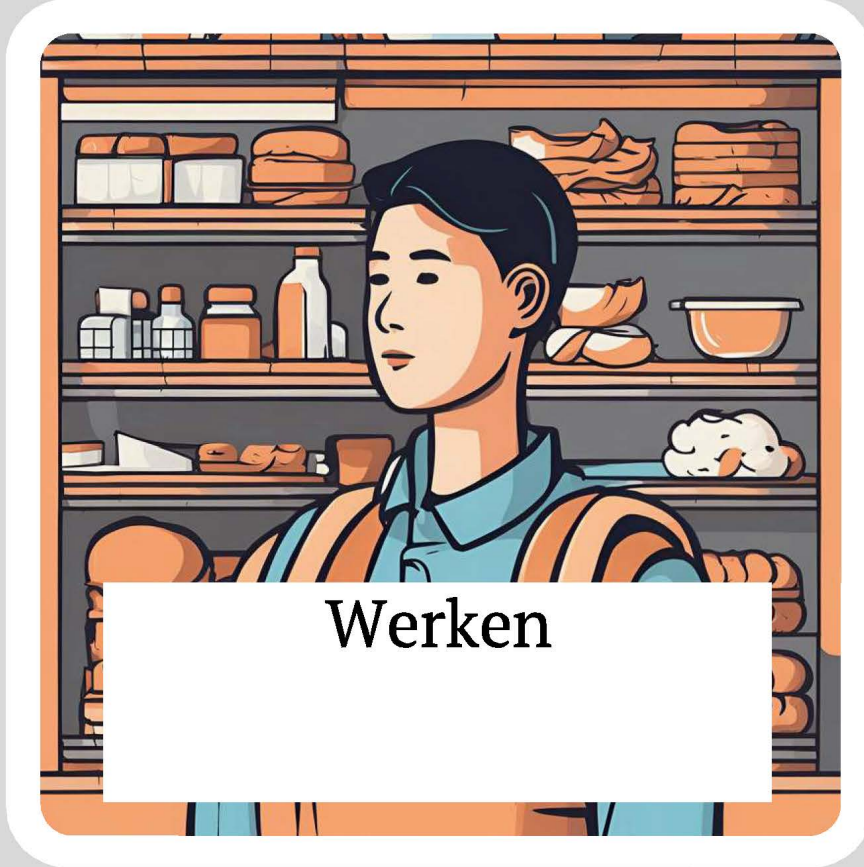
Werken

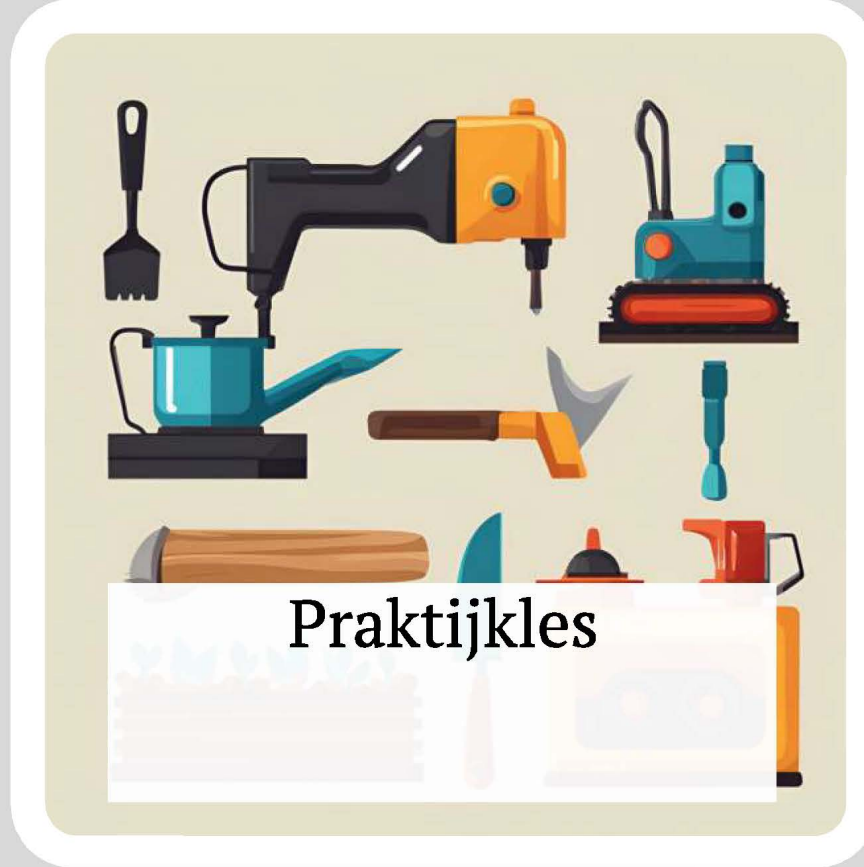
Praktijkles

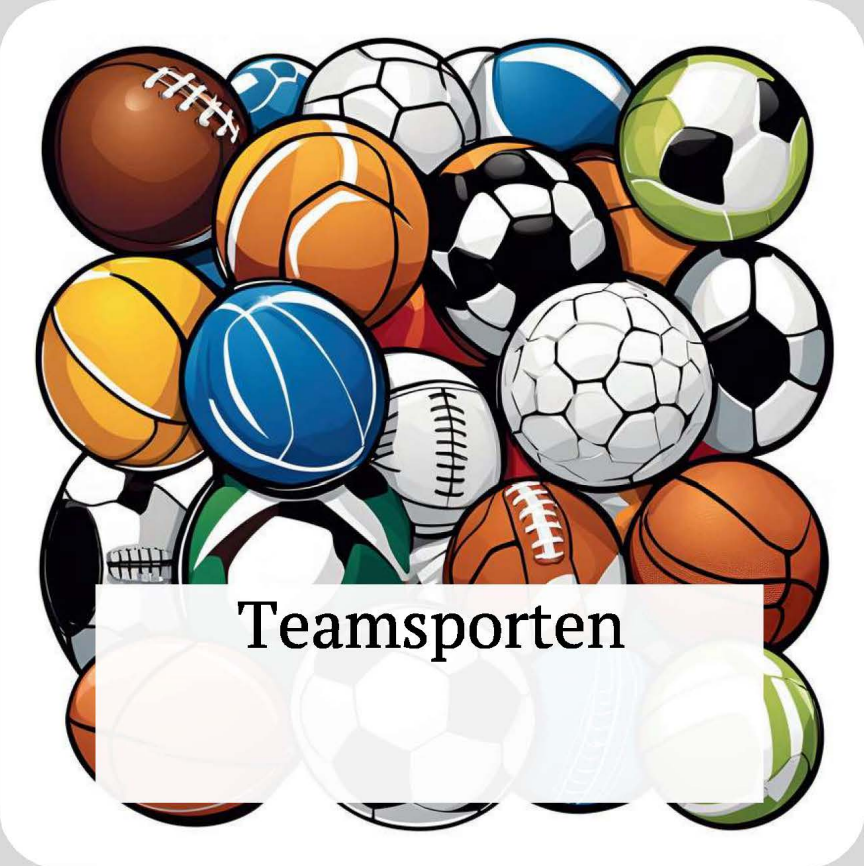
Teamsporten

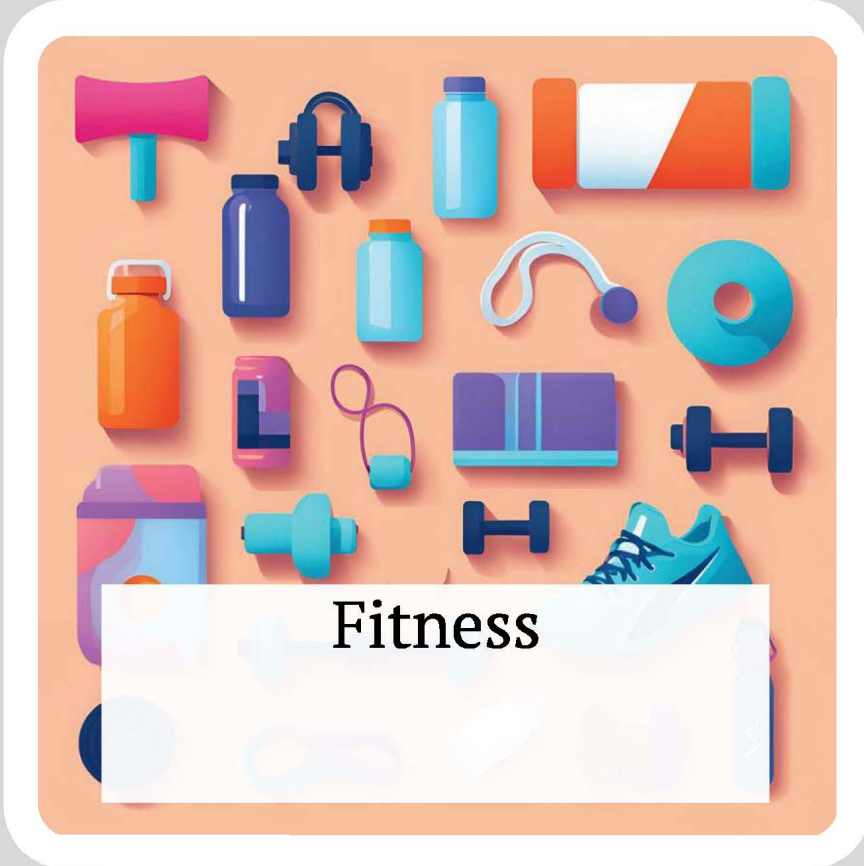
Fitness

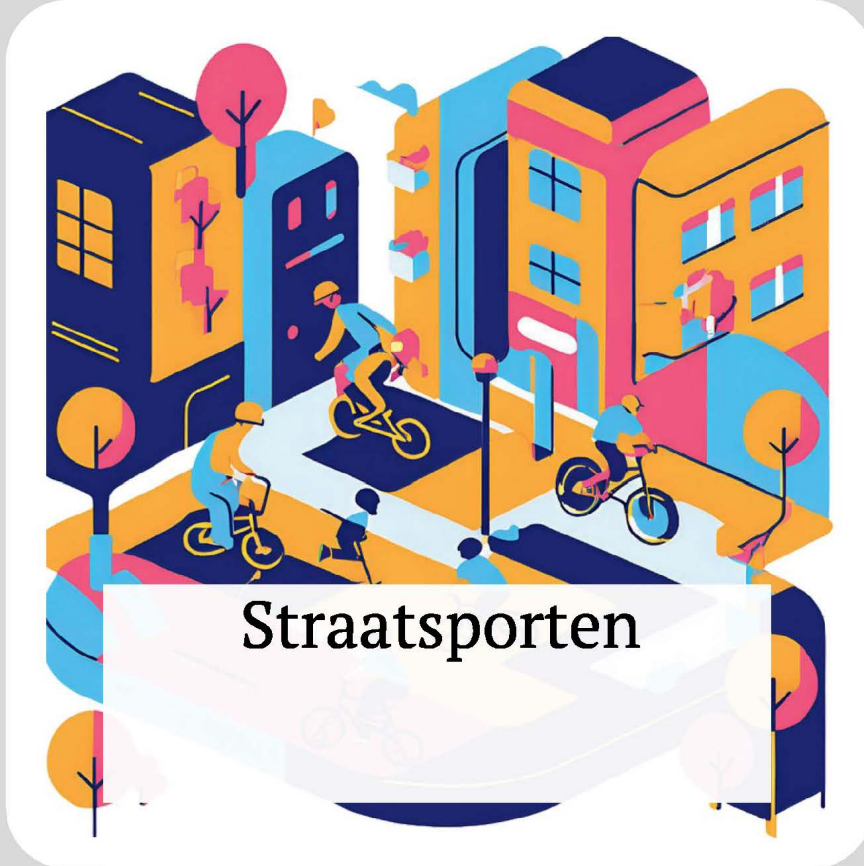
Straatsporten

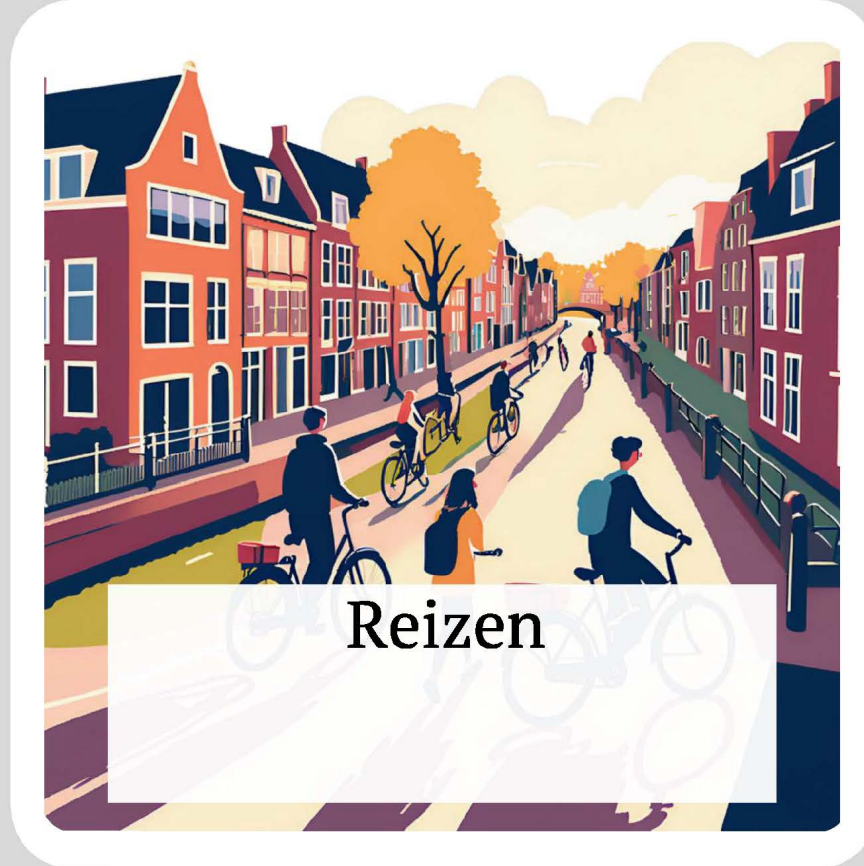
Reizen

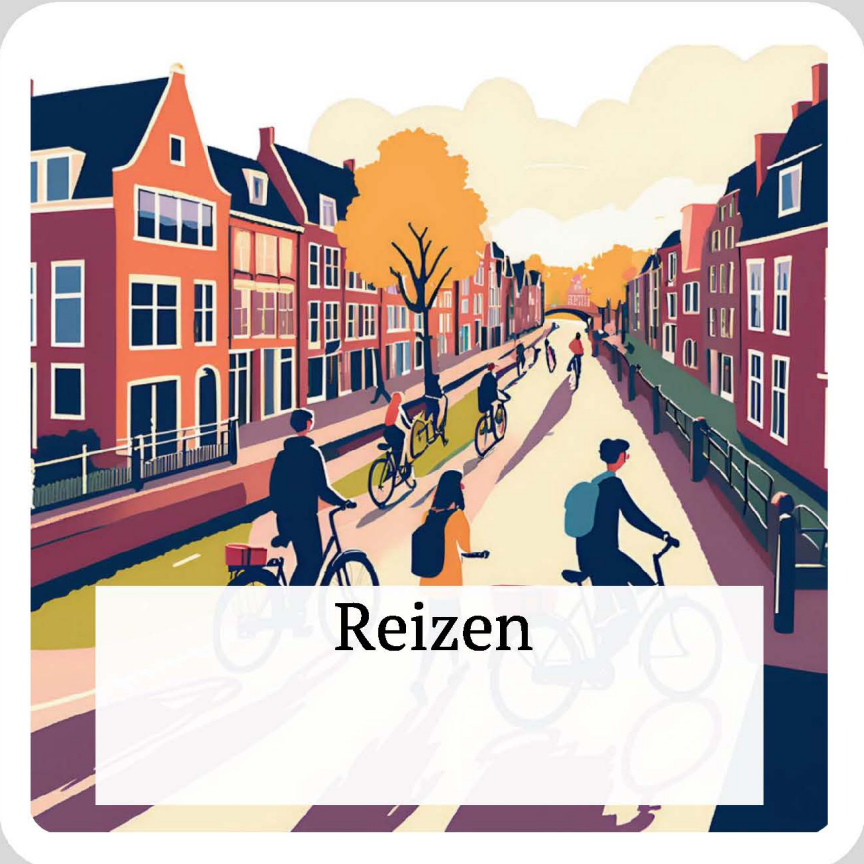
Reizen

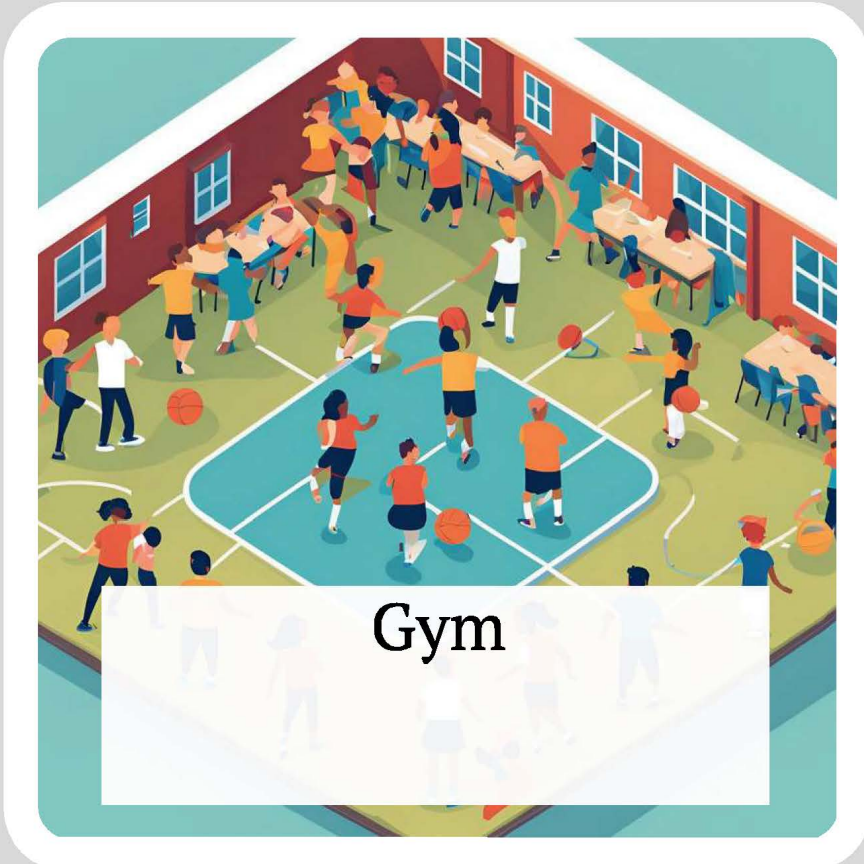
Gym

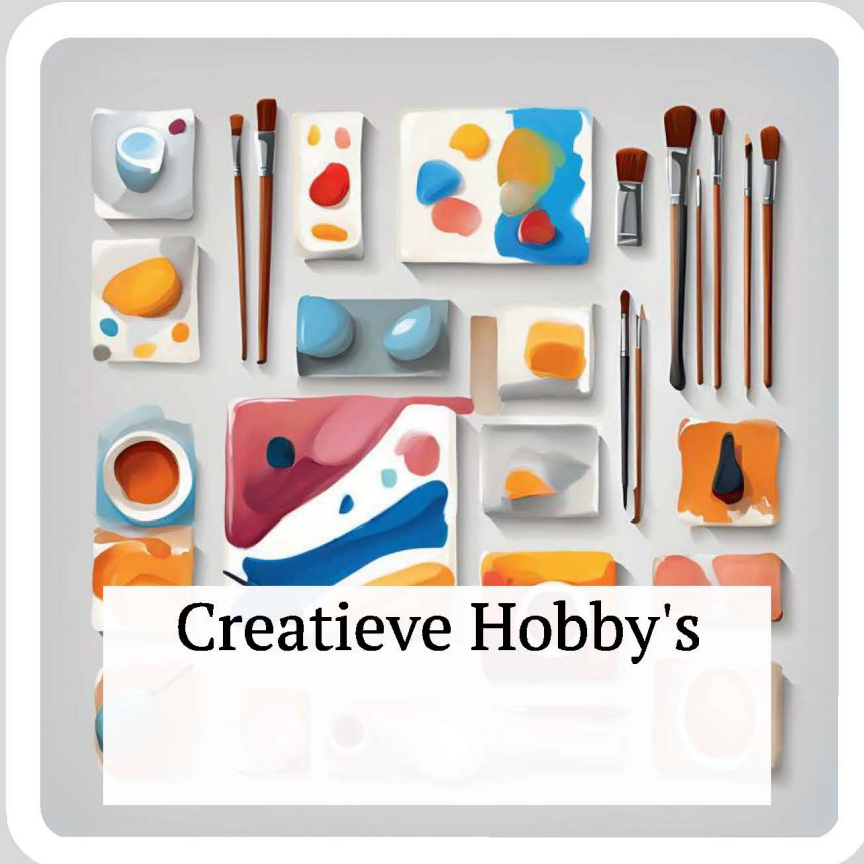
Creatieve Hobby's

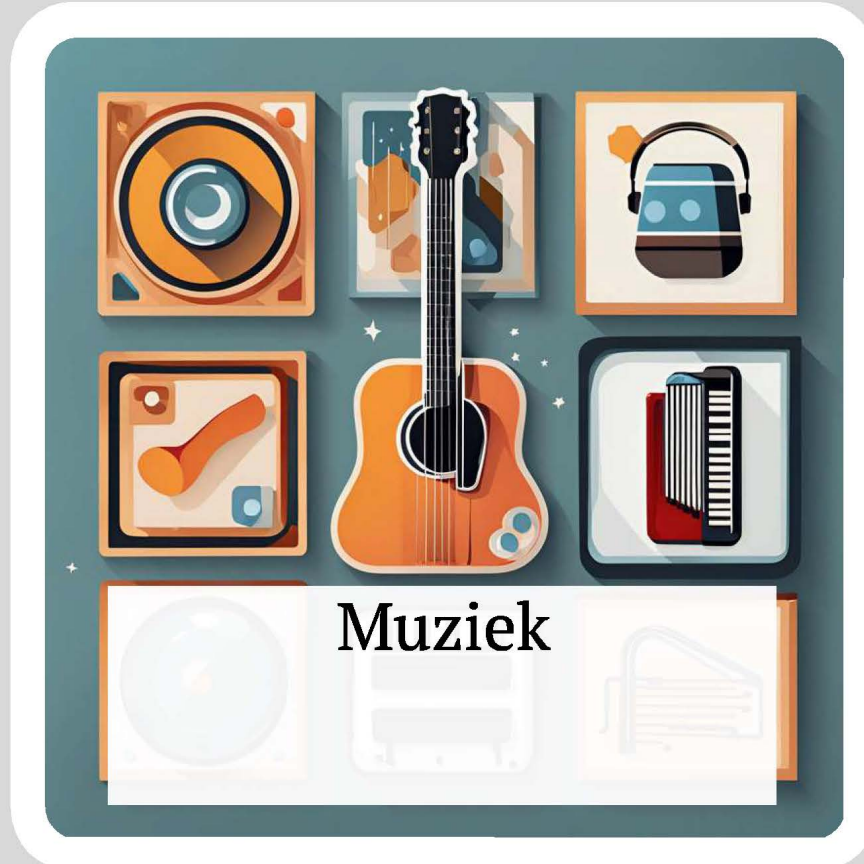
Muziek

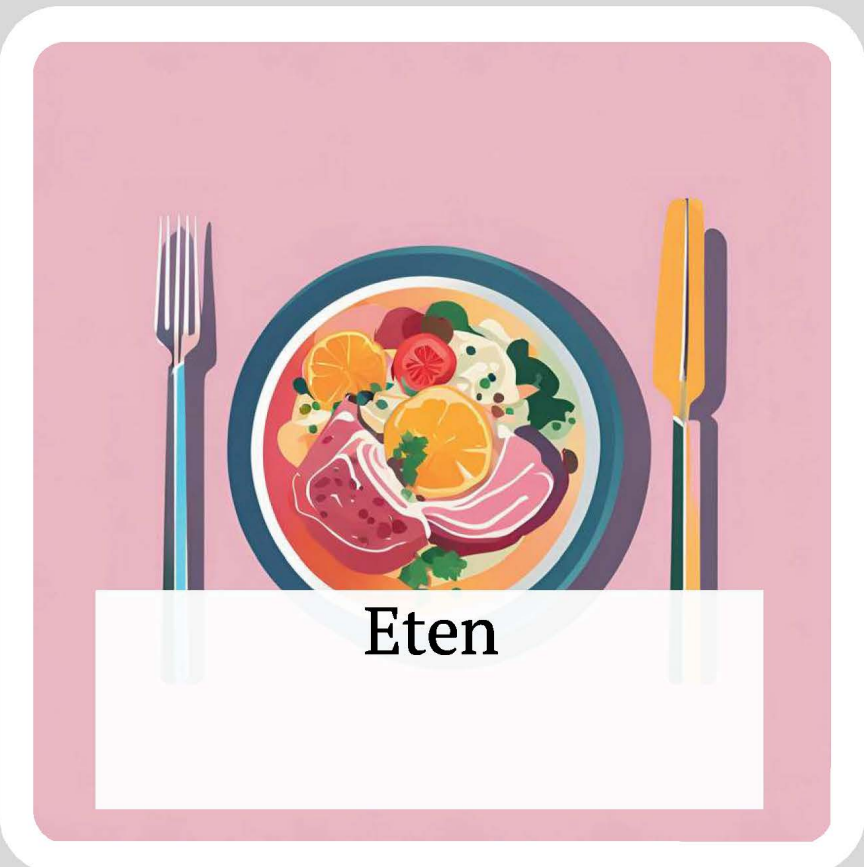
Eten

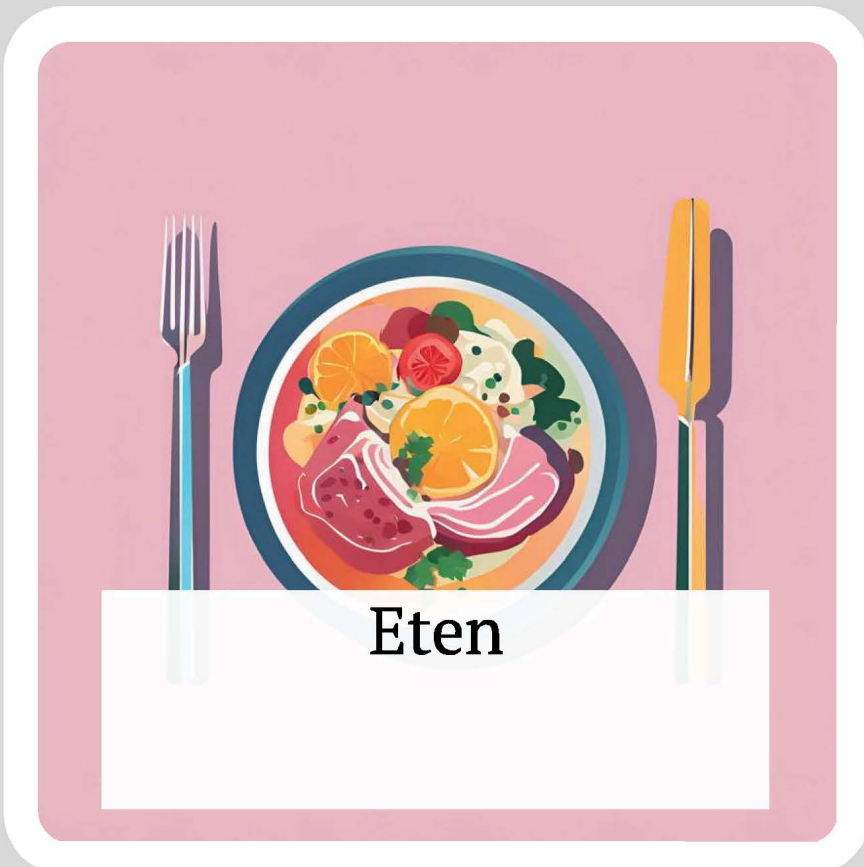
Eten

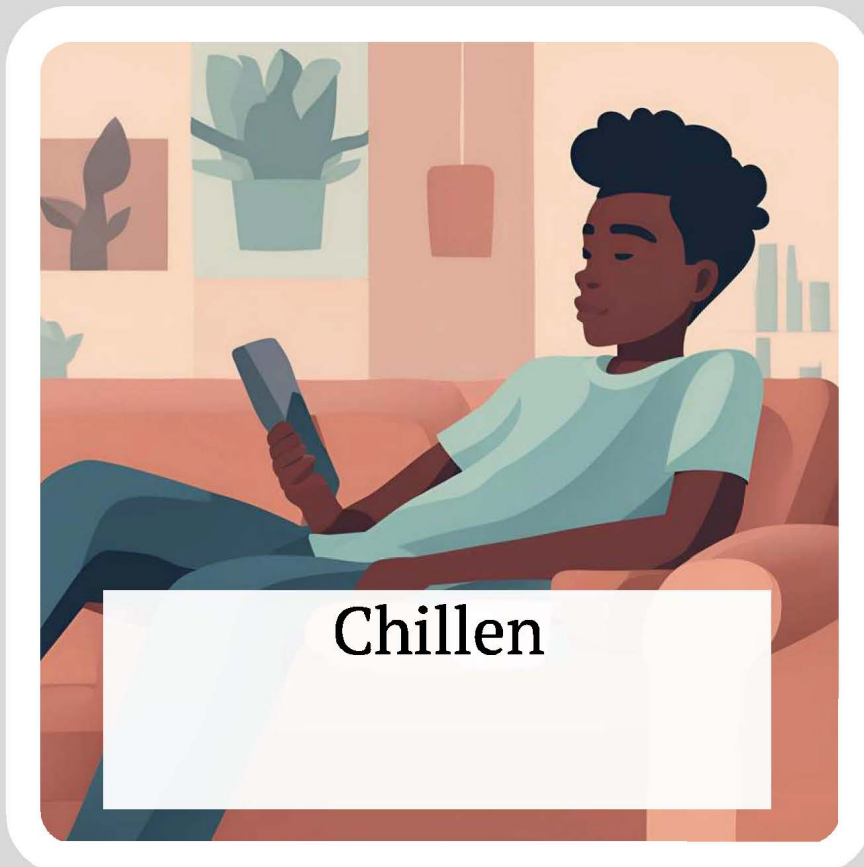
Chillen

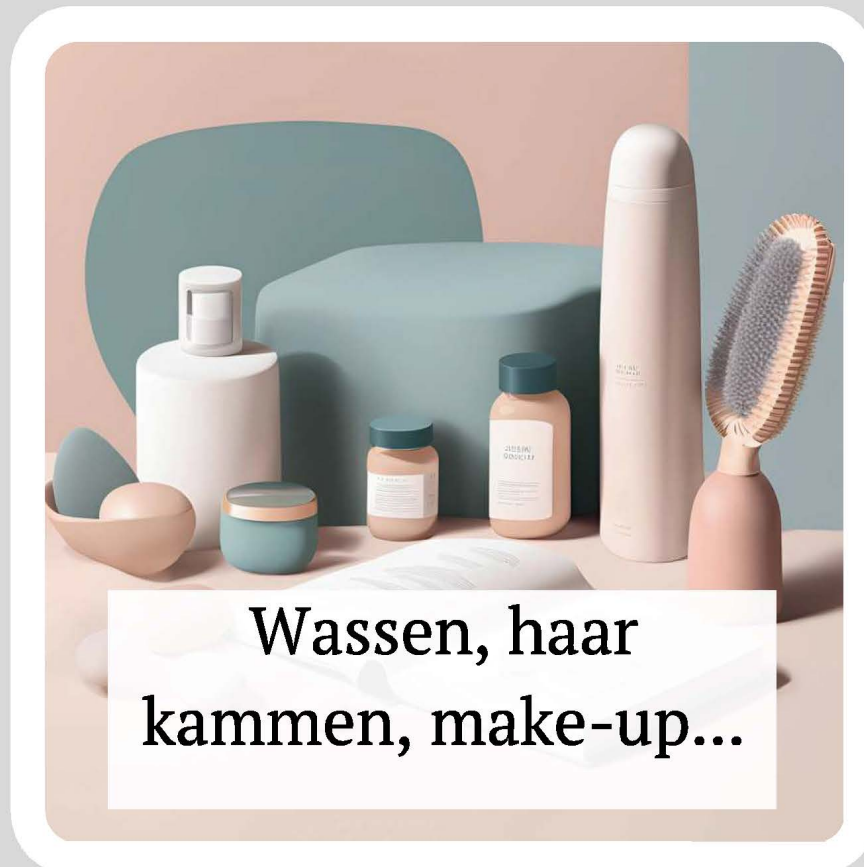
Wassen, haar
kammen, make-up...

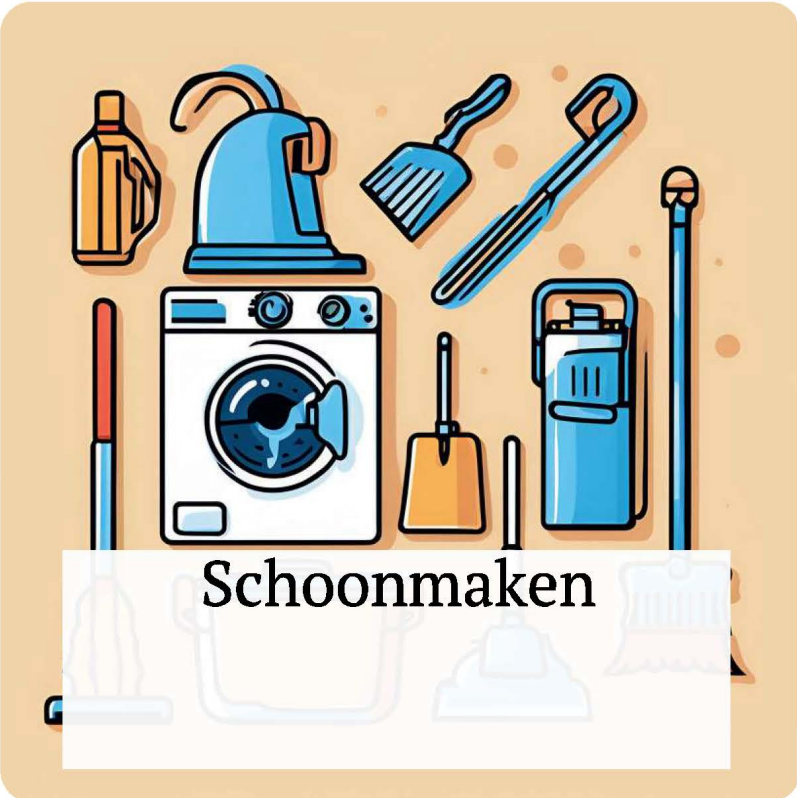

Schoonmaken

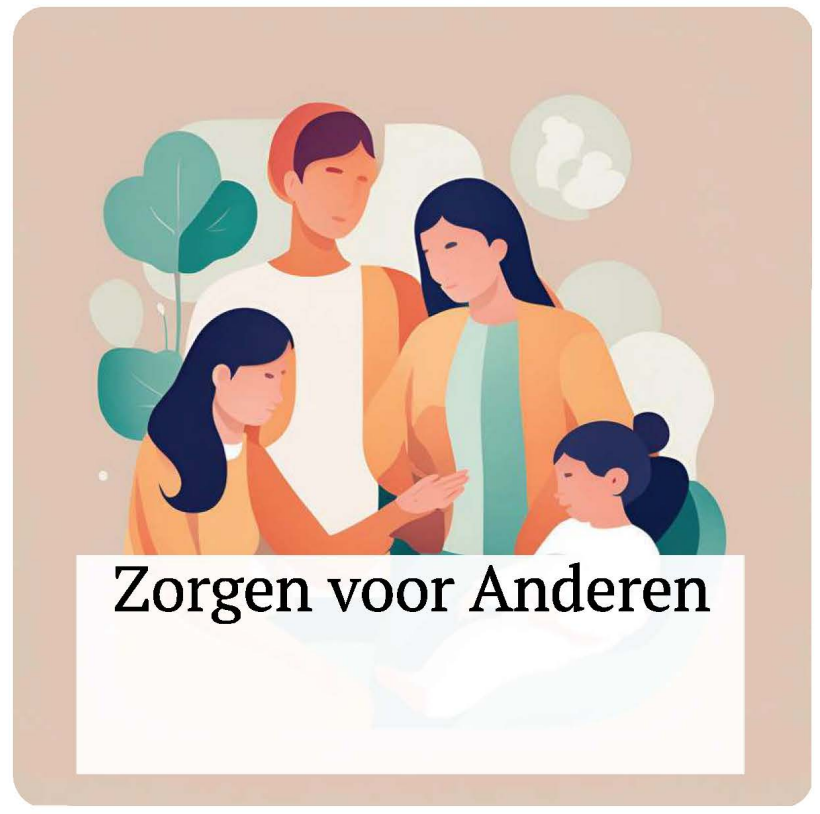

Zorgen voor Anderen

Familie

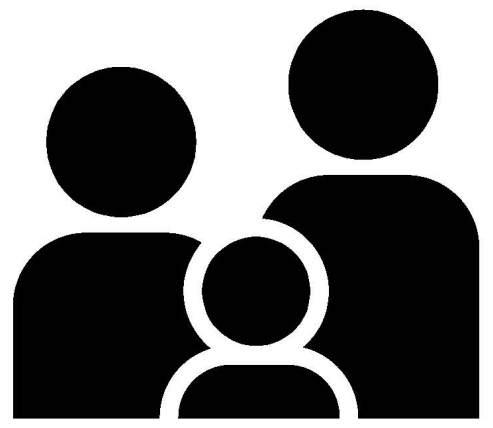

Vrienden

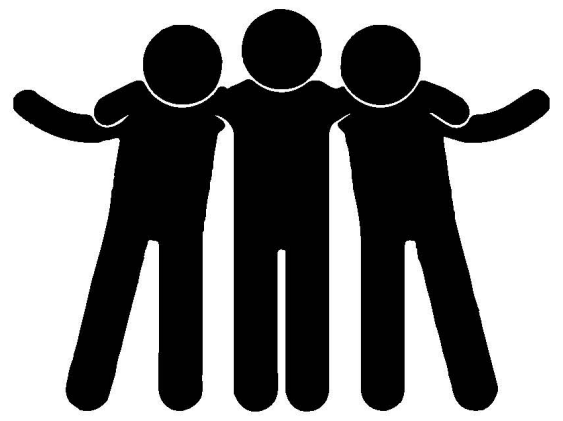

Leraar

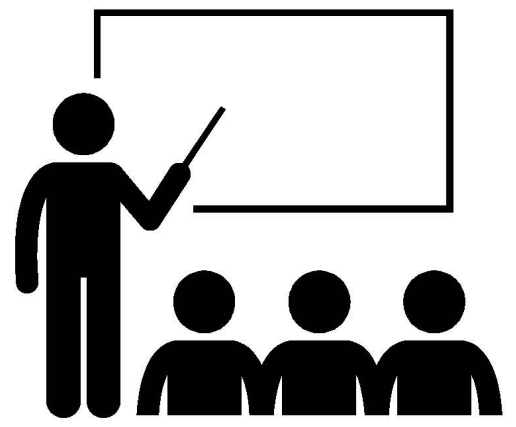

Coach

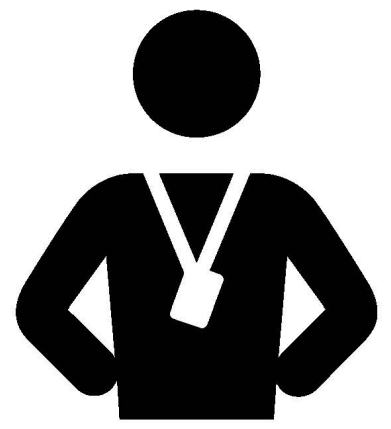

Huisdieren

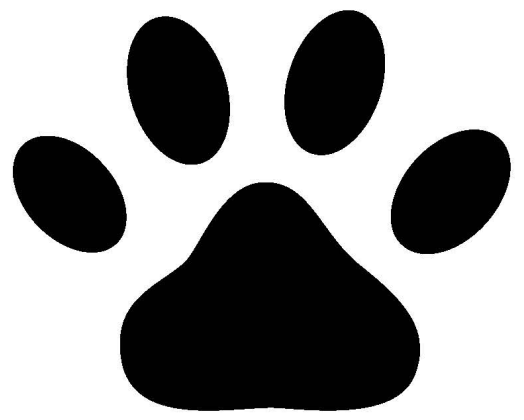

Buren

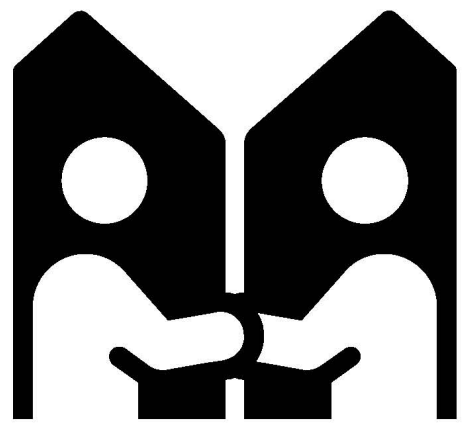

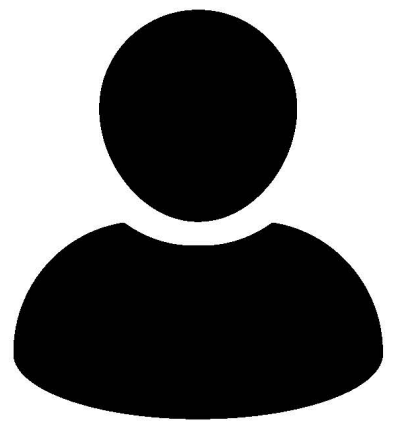

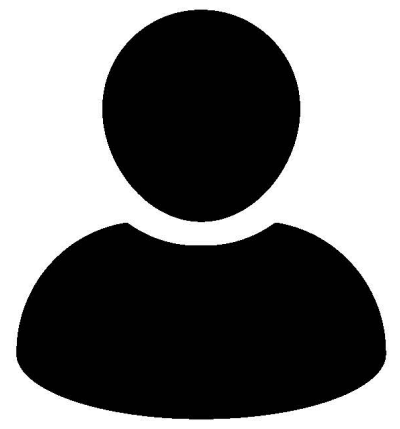

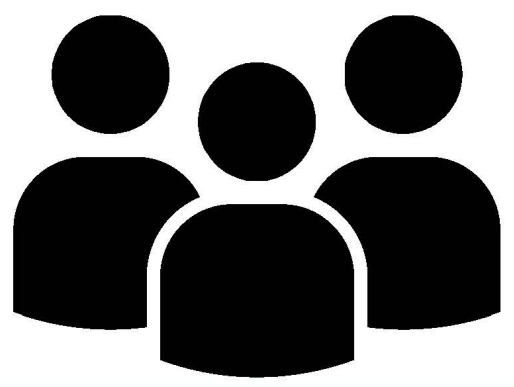

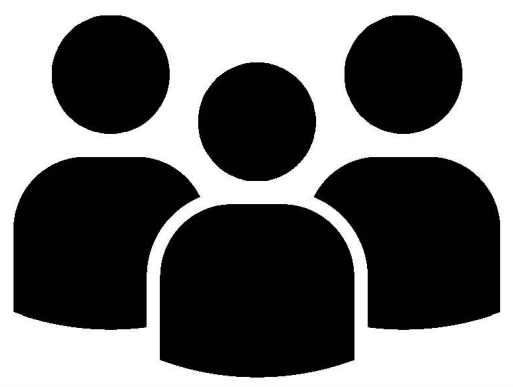

School

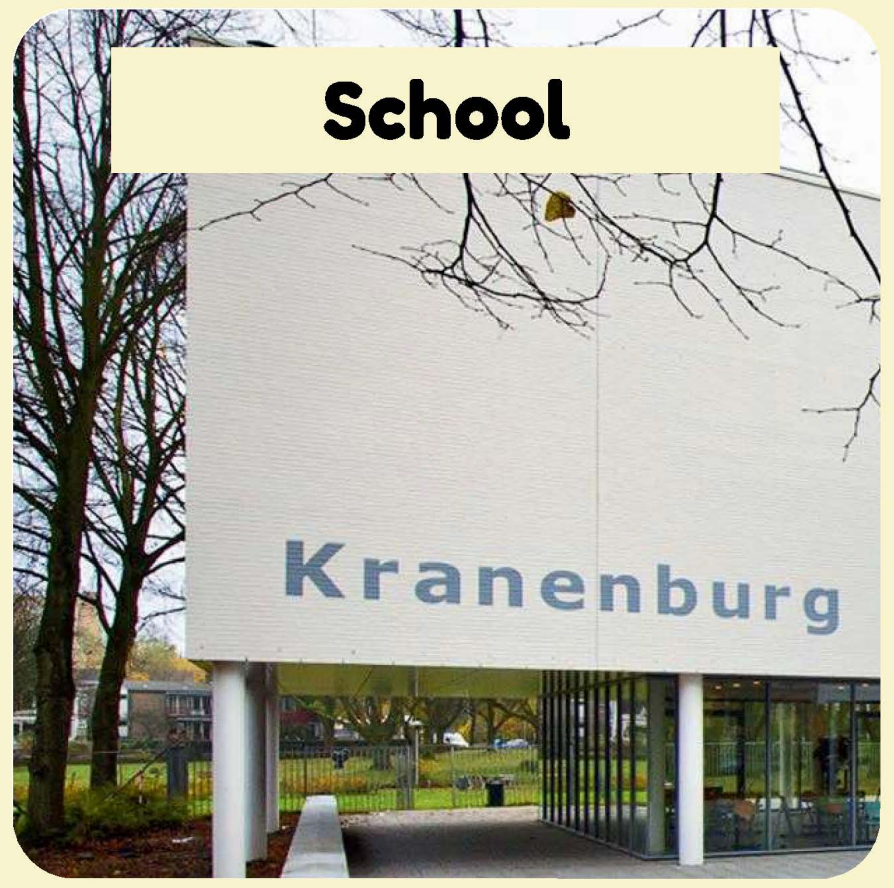


School

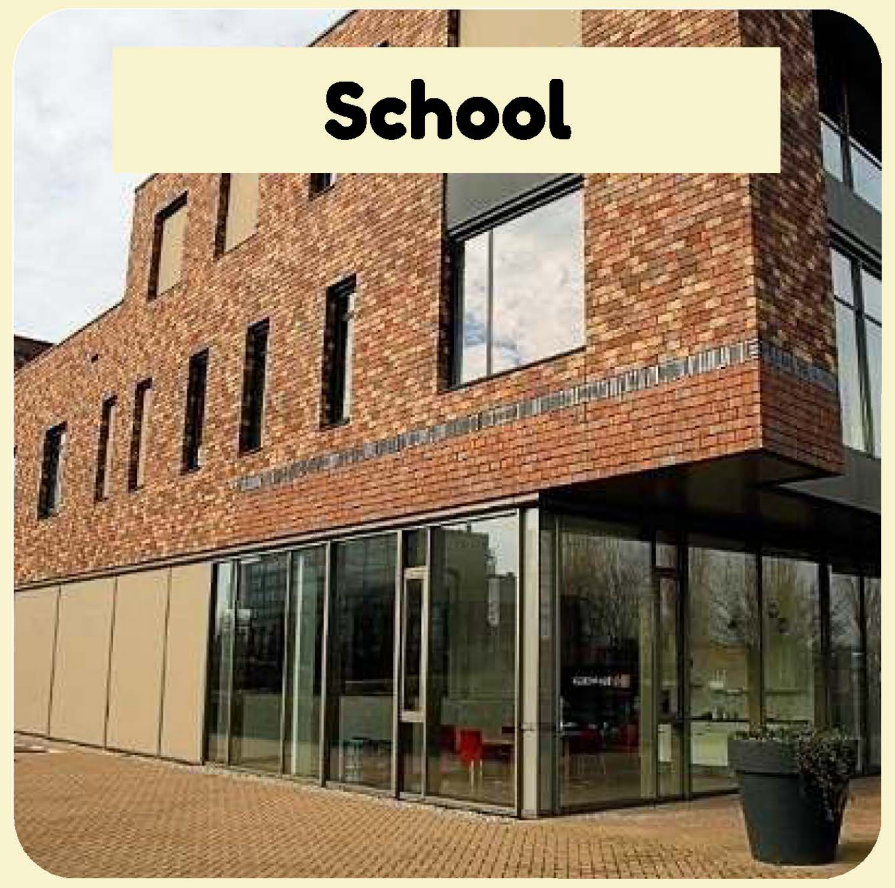

### Thuis

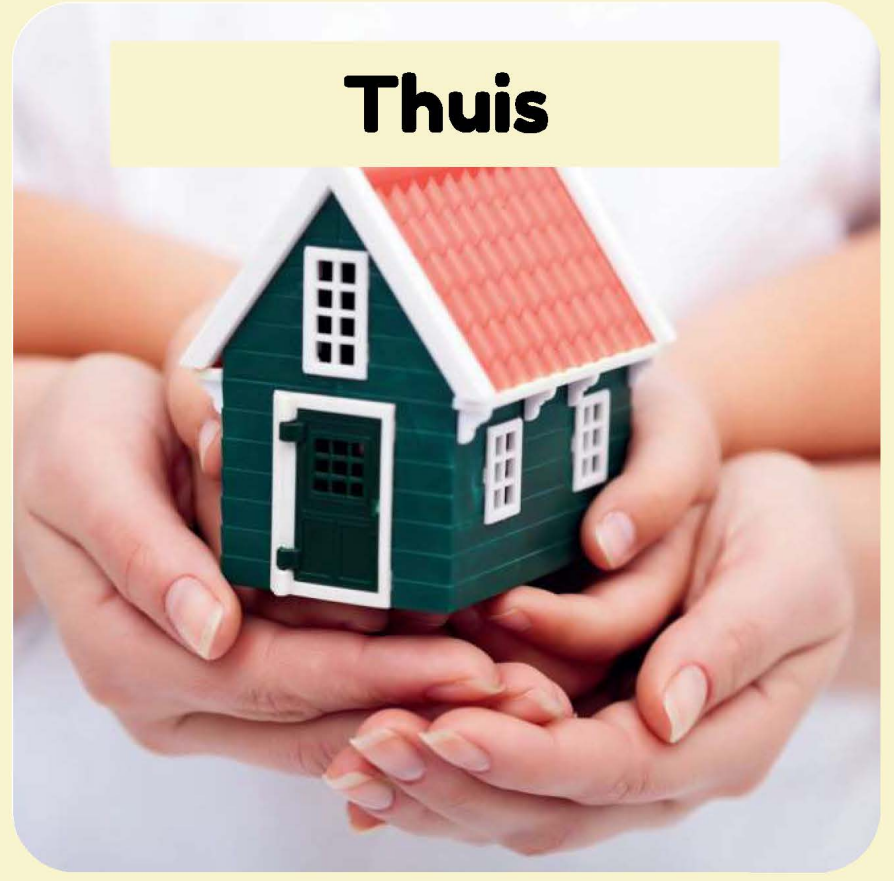

### Sportclub

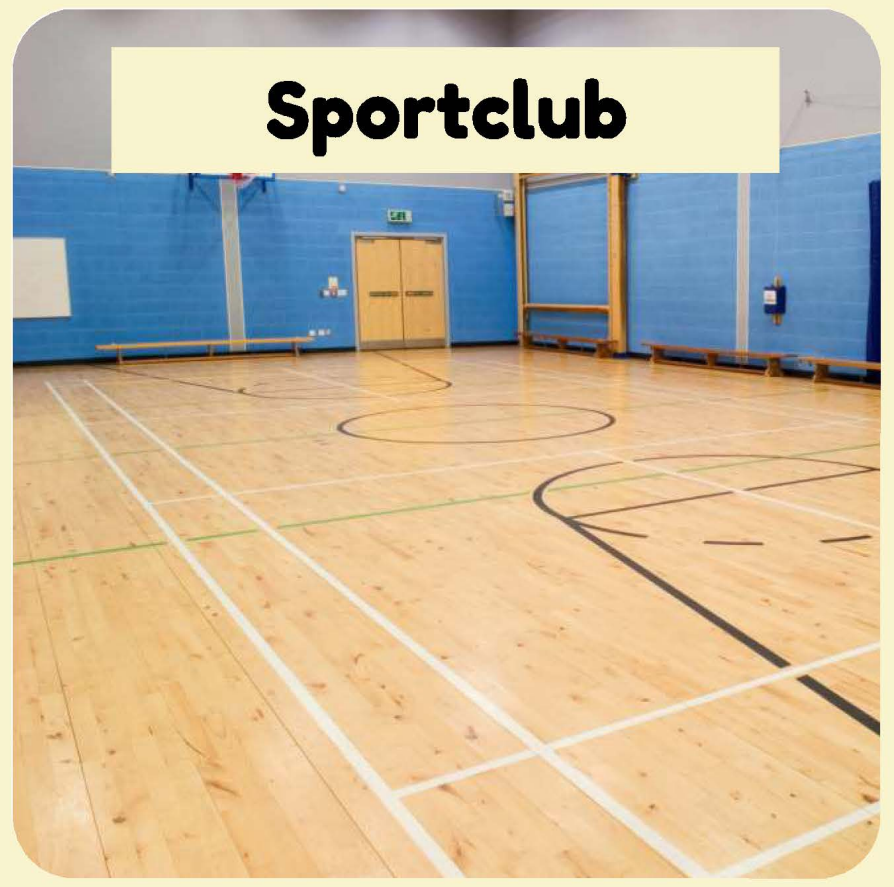

### Buiten

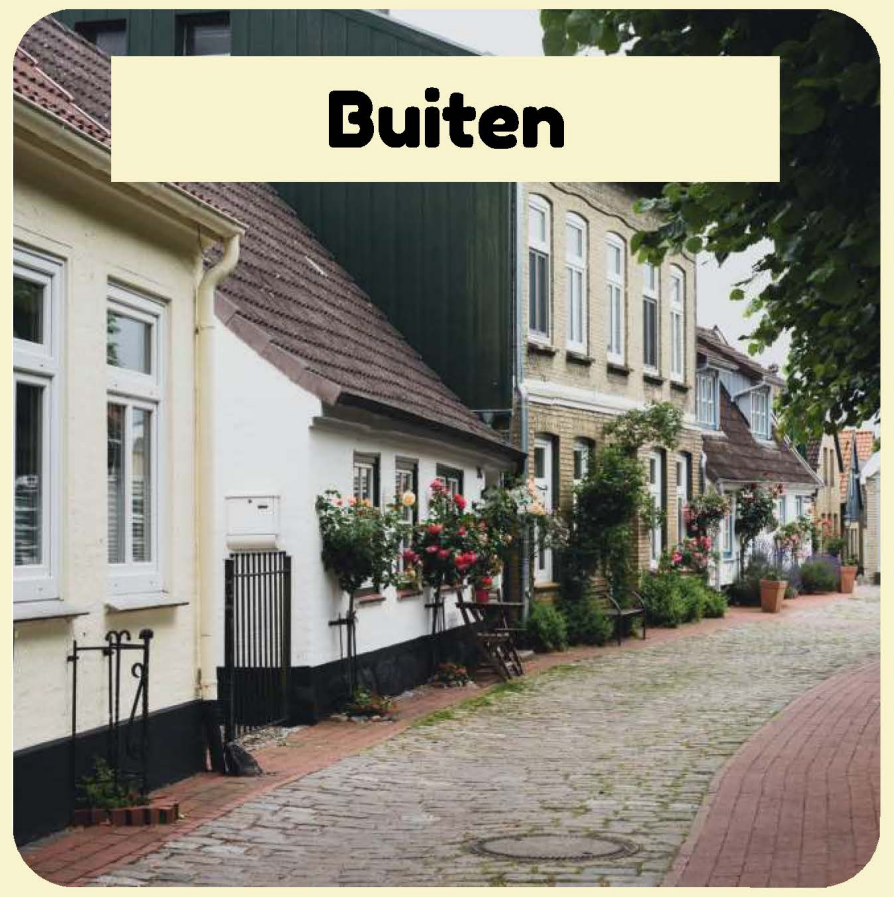

### Parken

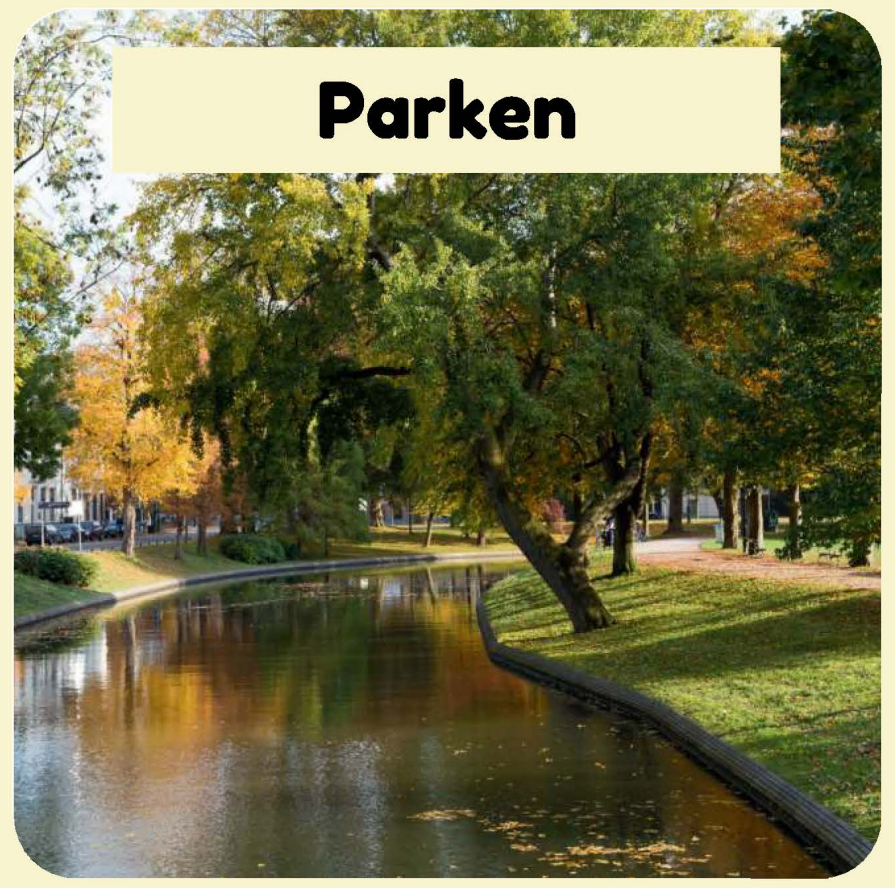

### Fitnesscentrum

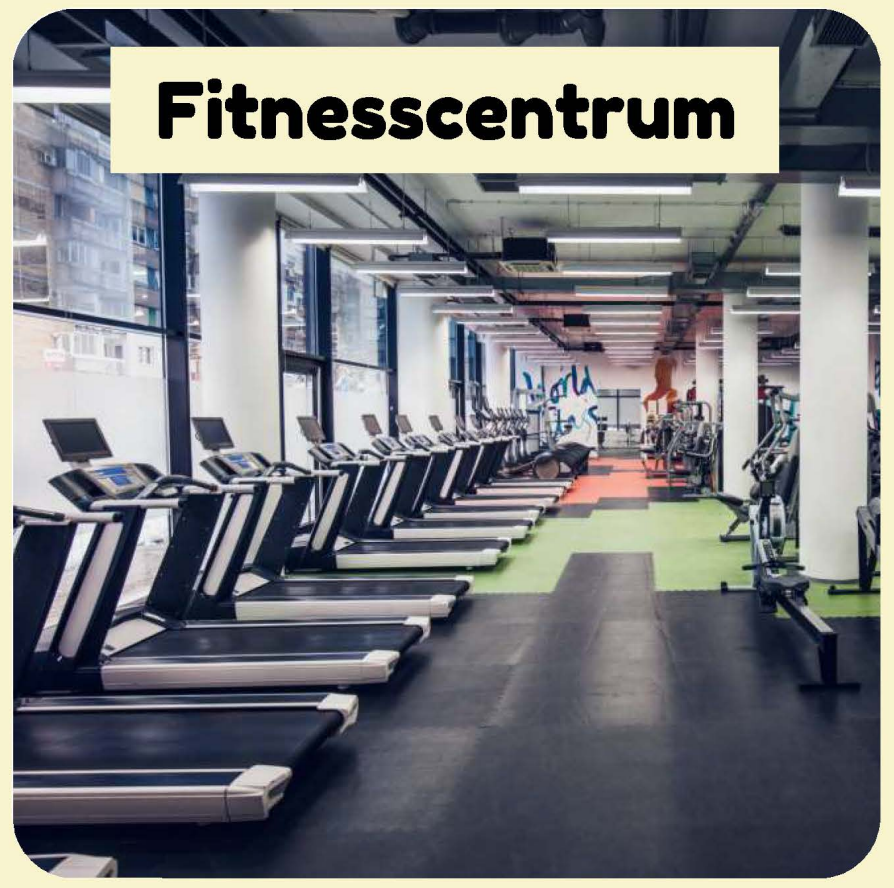

### Kleren

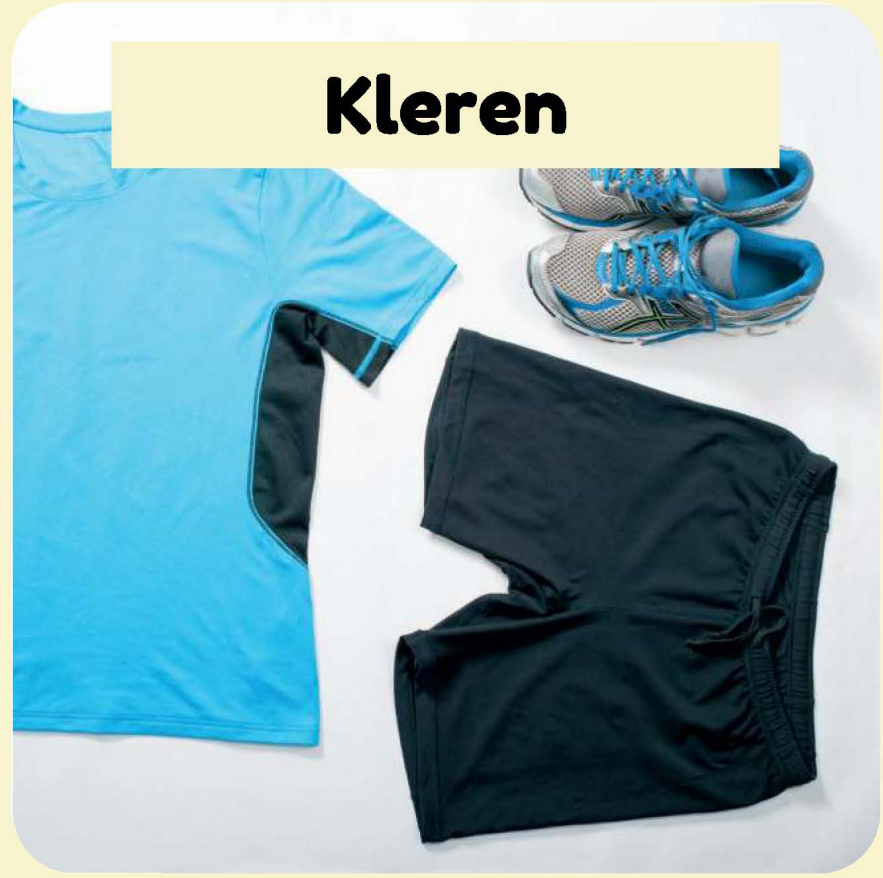

### Sportartikelen

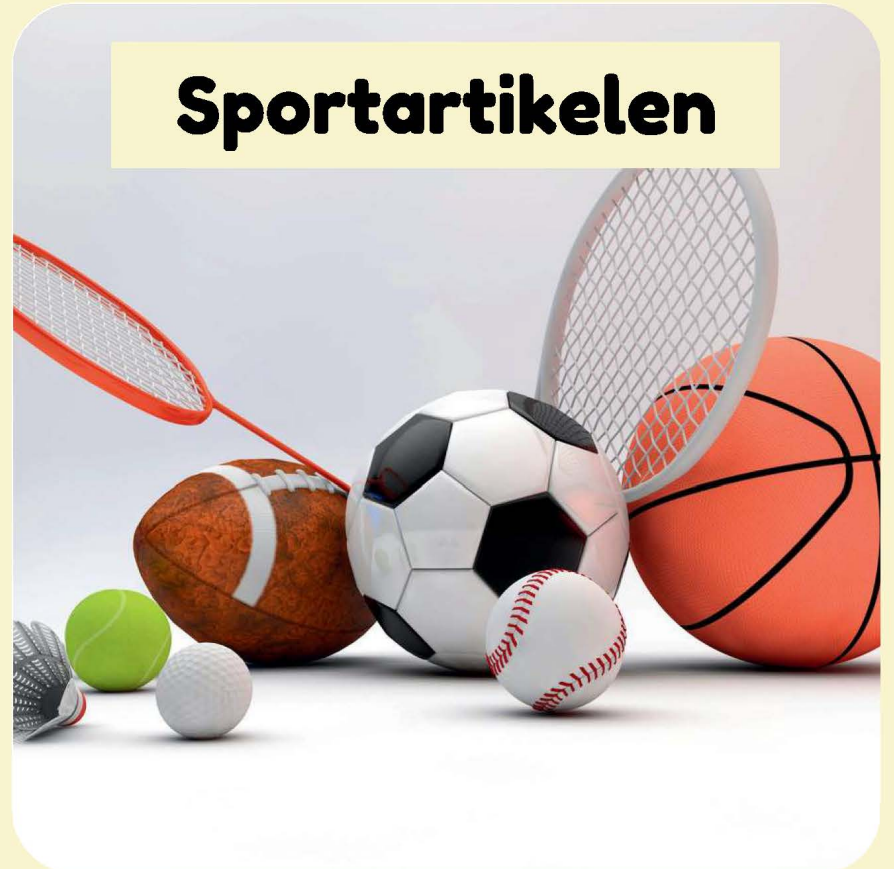

### Fitnessspullen

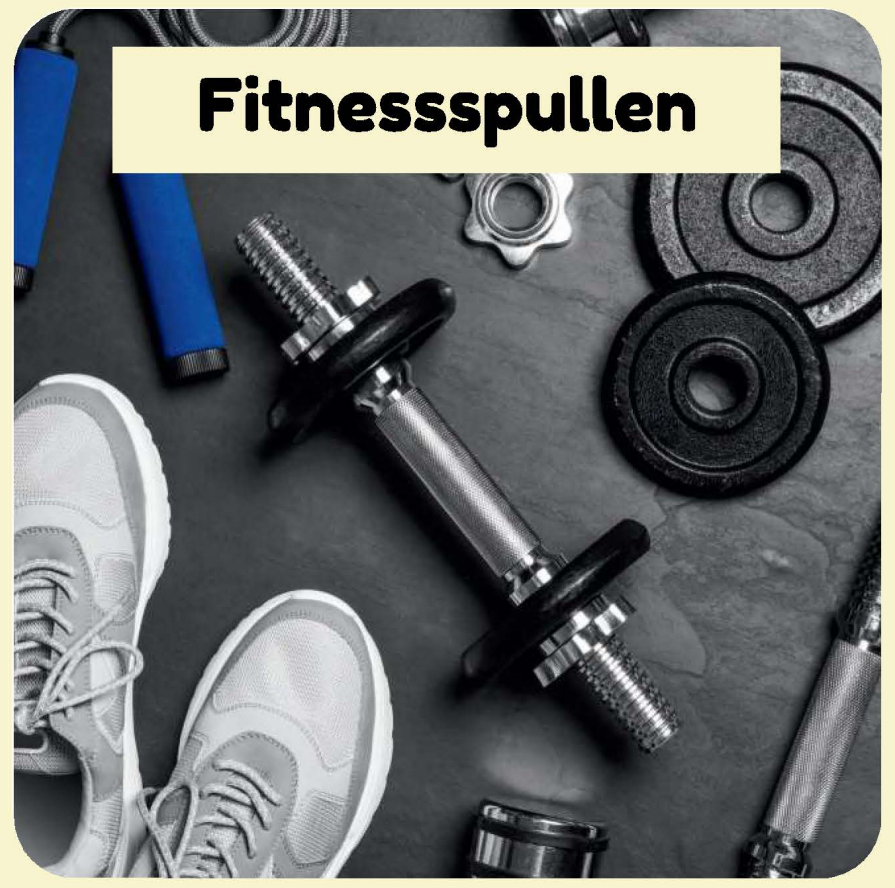

### Fiets

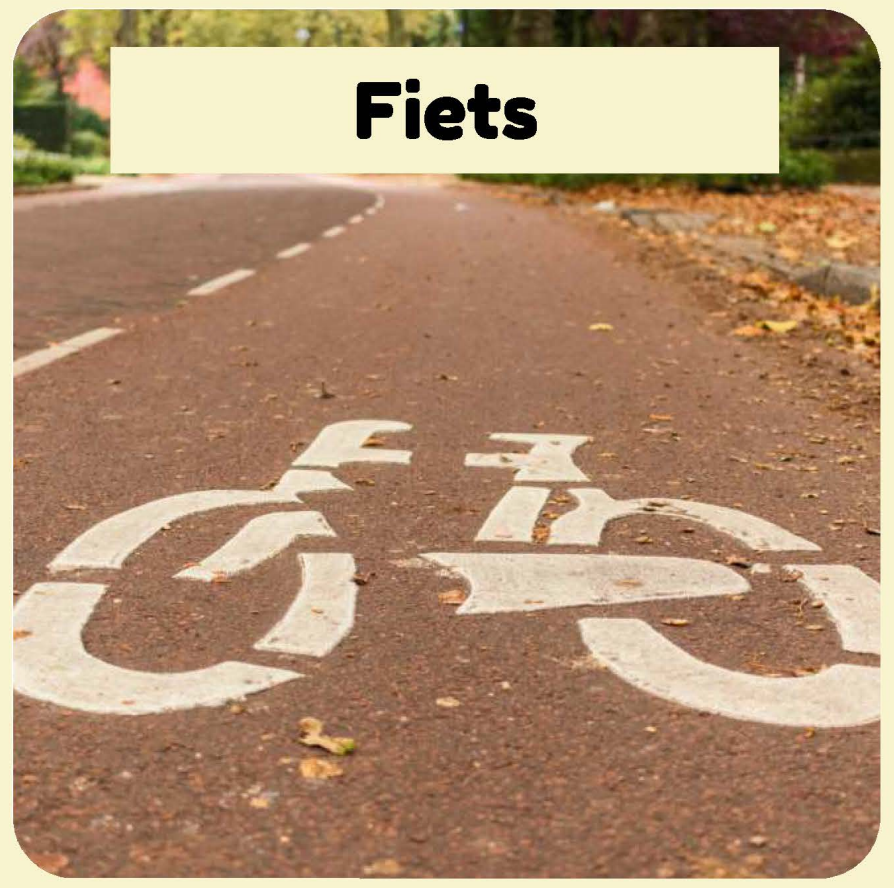

### Openbaar Vervoer

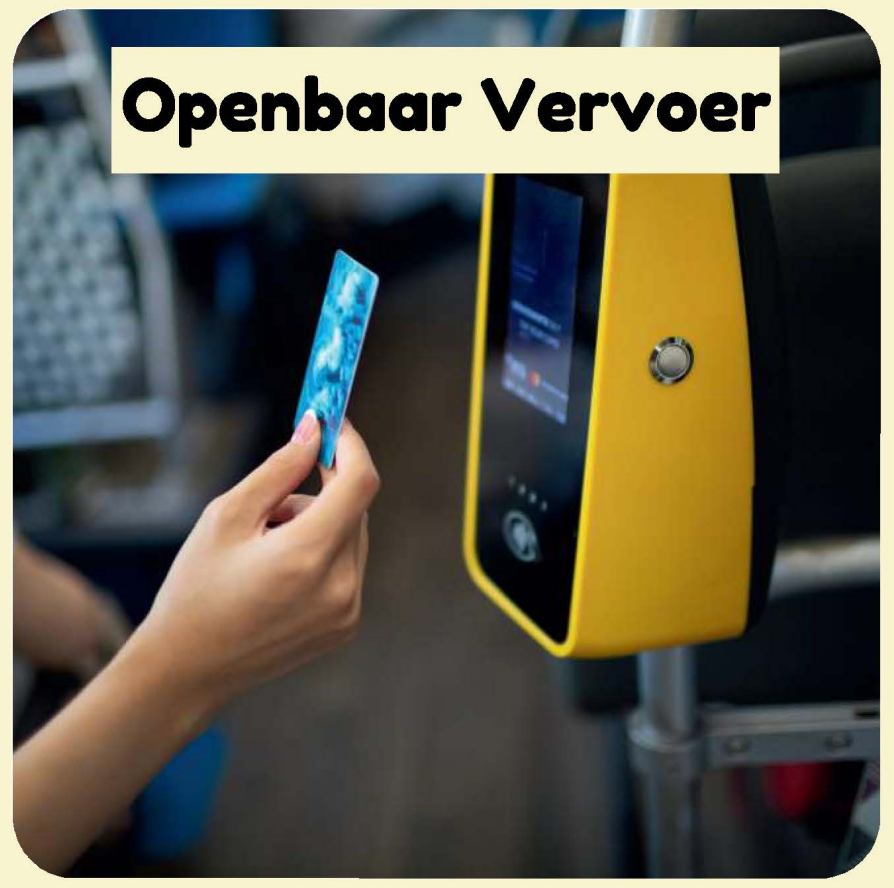

## Enthousiasme

Wat maakt je enthousiast over sport en bewegen, en wat niet?

## Plezier

Hoe graag sport en beweeg jij? Waarom?

## Gevoelens

Hoe voel jij je tijdens het sporten en bewegen? Waarom?

## Mijn lichaam
Voel jij je sterk en vol energie om te sporten en bewegen? Waarom wel of niet?

## Hoe je jezelf ziet
Hoe voel jij je over jezelf als je sport en beweegt? Waarom?

## Resultaten
Wat denk je dat er gebeurt als je sport en beweegt? Waarom?

## Plan
Hoe plan je momenten in om te sporten en te bewegen? Vergeet je het?

## Gezondheid
Waarom is sporten en bewegen belangrijk volgens jou?

## Manier van leven
Hoe pas je sport en beweging in je dagelijkse leven in?

## Beter worden
Hoe weet je of je beter wordt in sport en beweging?
