# Supplementary material for: Physical Activity Determinants Among Adolescents With Mild Intellectual Disability in Dutch Practical Education: Exploratory Sequential Mixed Methods Study
Source: JMIR Form Res. 2026 Sep 30;10:e89518. doi: 10.2196/89518 (PMC13626643; doi:10.2196/89518)
Supplement: Supplementary file 4 [file formative-v10-e89518-s004.pdf]

Wat maakt sporten en bewegen **makkelijk** of **moeilijk** voor jou?

Een beetje makkelijk

Makkelijk

Heel makkelijk

Mwah...

Een beetje moeilijk

Moeilijk

Heel moeilijk

Jouw conditie

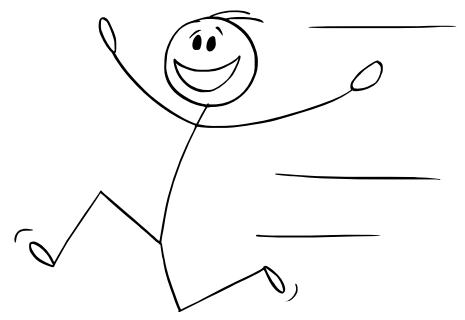

1

Waneer je begon met sporten

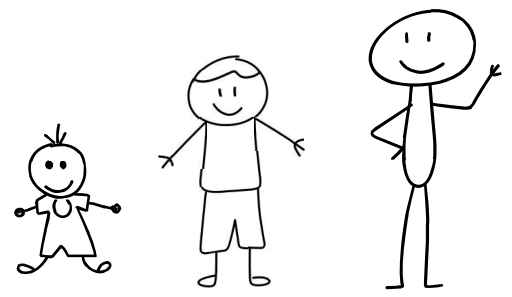

2

Wat je weet over sporten en bewegen

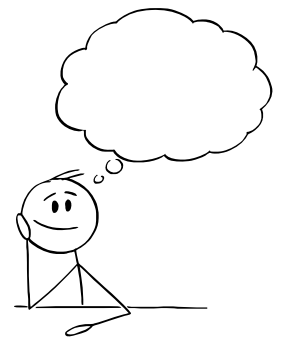

3

Sporten met anderen

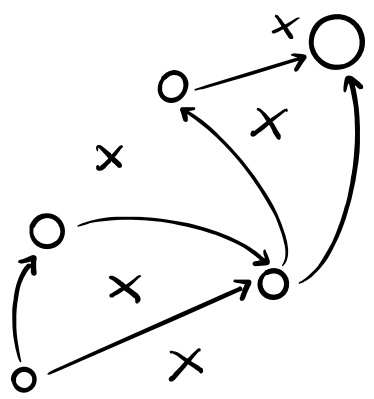

4

Hoe je voel over sporten en bewegen

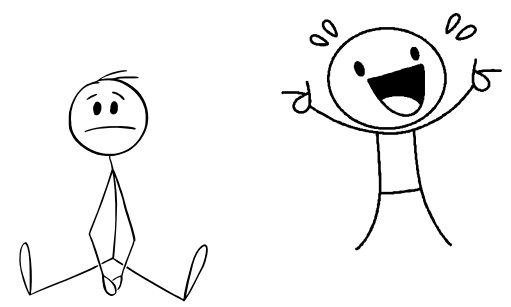

5

Jouw zelf vertrouwen

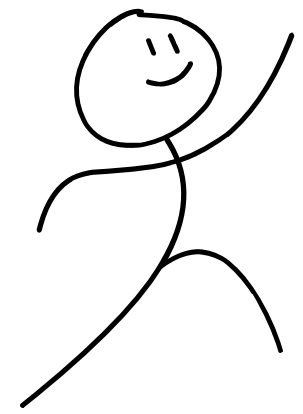

6

Hoe je je lichaam ziet

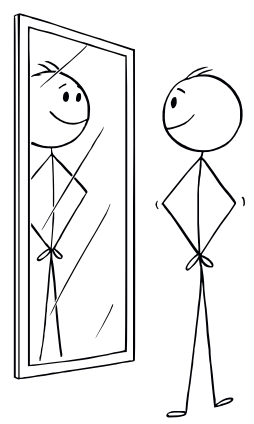

7

Jouw doelen

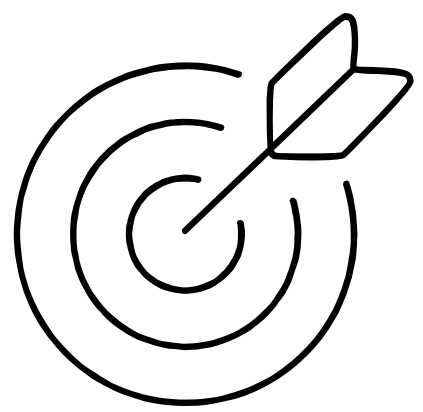

8

Dingen zelf doen en kiezen

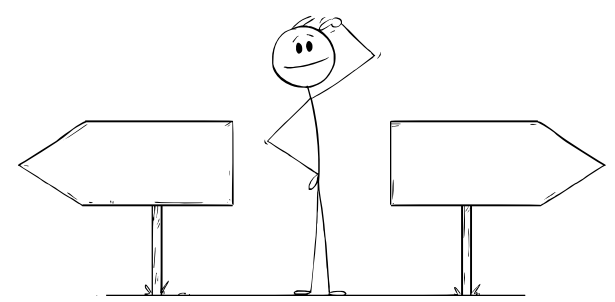

9

Jouw dagelijkse gewoontes

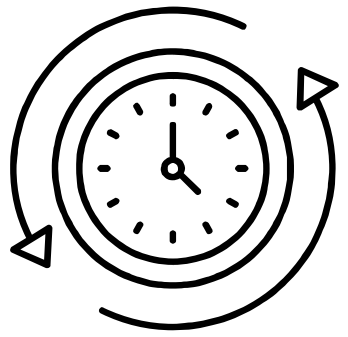

10

Jouw vrienden

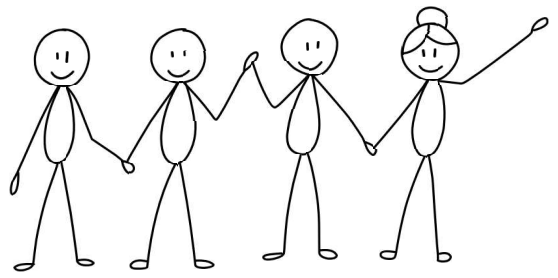

11

Steun van je ouders

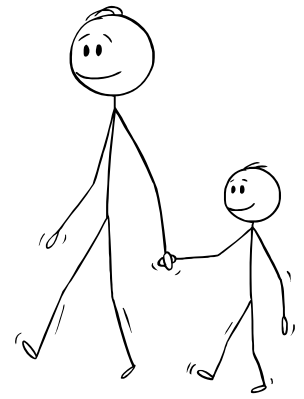

12

Wat je ouders weten over sporten en bewegen

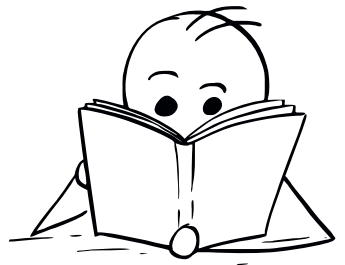

13

Jouw leraren

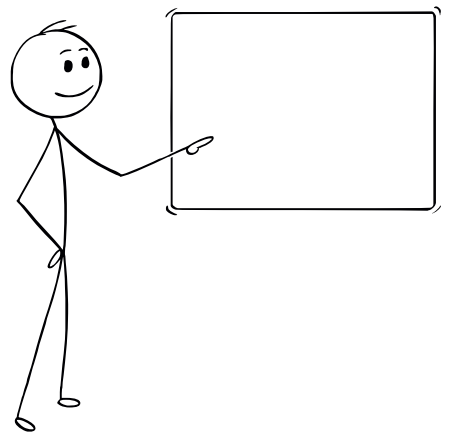

14

Jouw sportcoaches

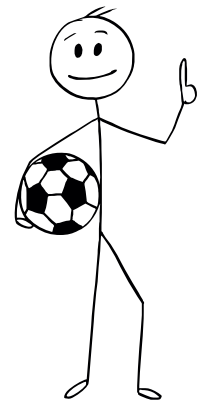

15

Mensen tegen wie je opkijk

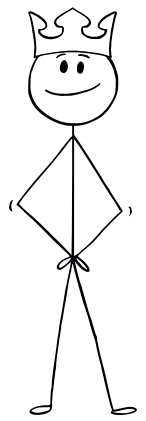

16

Sociale media

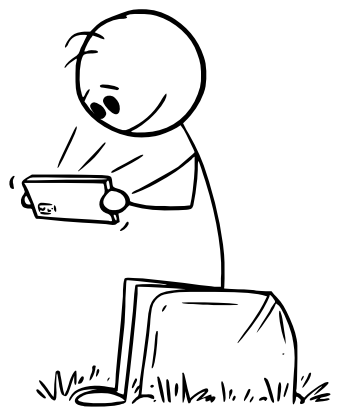

17

Wedijveren met anderen

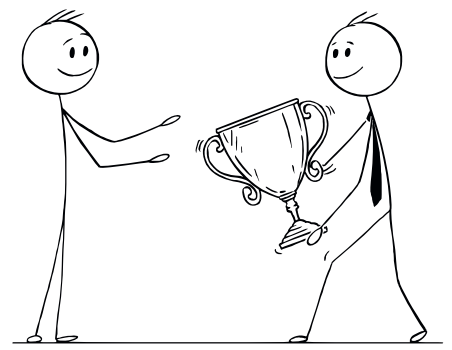

18

Populaire sporten en bewegen

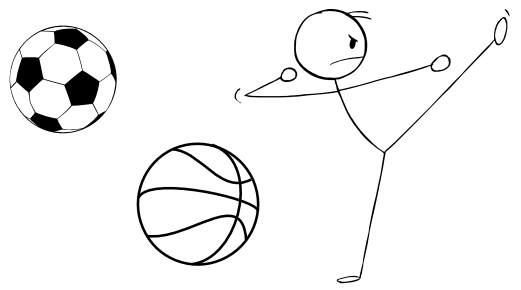

19

Leuke sporten

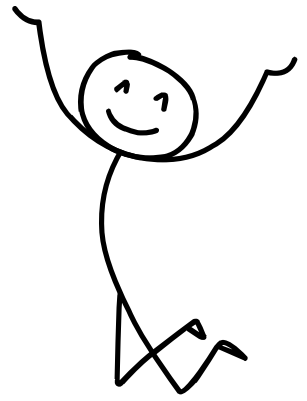

20

Jouw tijd na school

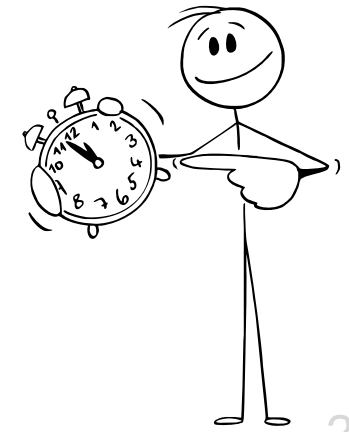

21

Jouw zin na school

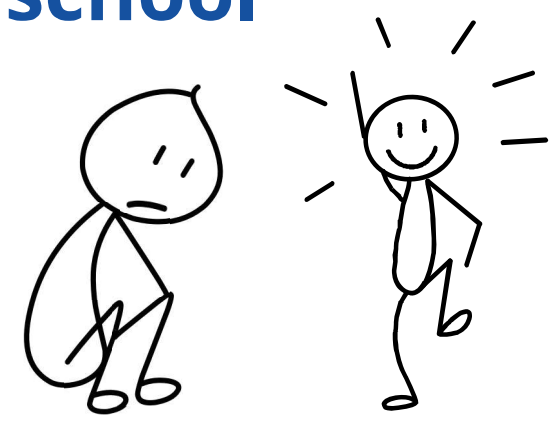

22

Sporten op school tijdens schooltijd

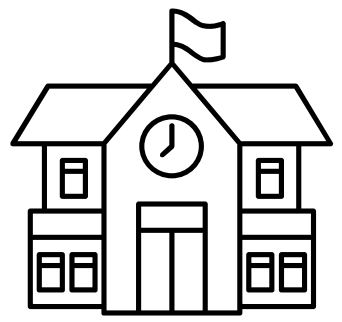

23

Sporten op school na schooltijd

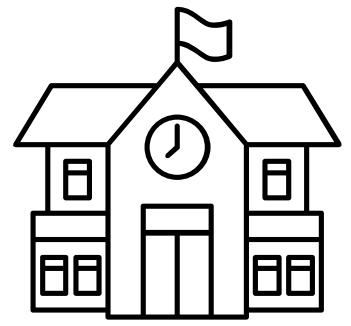

24

Sporten en bewegen buiten school

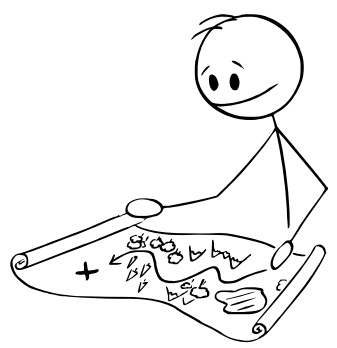

25

Hoe dichtbij sportplekken zijn

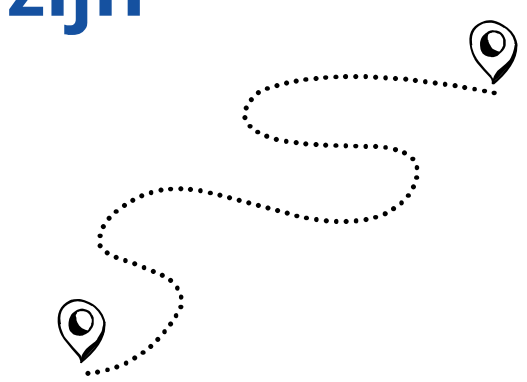

26

Naar school of werk gaan

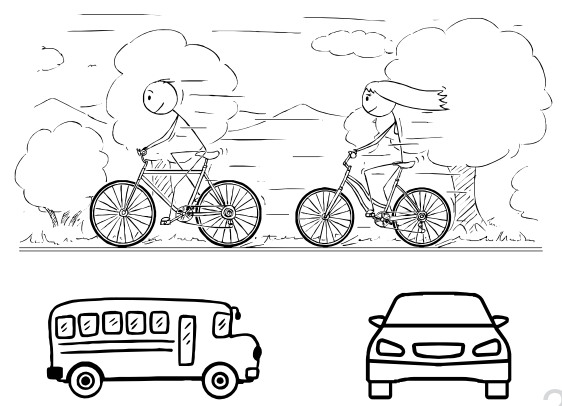

27

Geld voor sporten

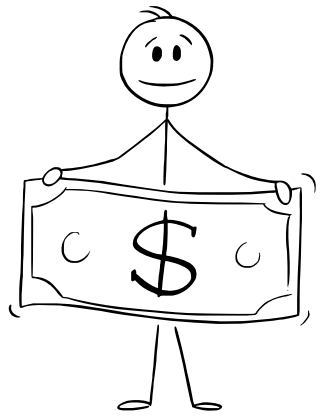

28

Sporten voor jongens en meisjes

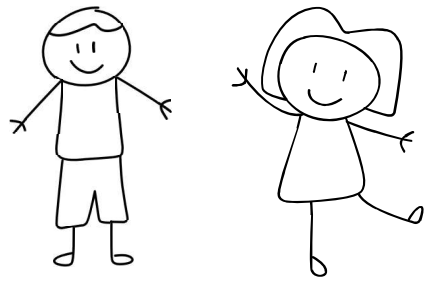

29

De cultuur van jouw familie

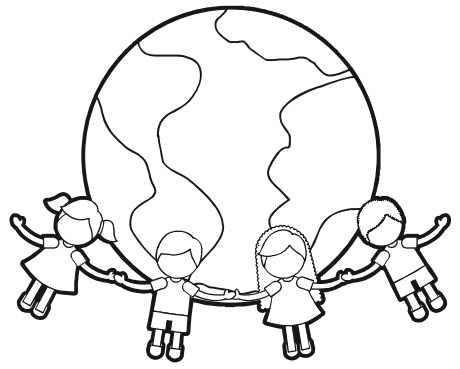

30
