# Supplementary material for: Physical Activity Determinants Among Adolescents With Mild Intellectual Disability in Dutch Practical Education: Exploratory Sequential Mixed Methods Study
Source: JMIR Form Res. 2026 Sep 30;10:e89518. doi: 10.2196/89518 (PMC13626643; doi:10.2196/89518)
Supplement: Supplementary file 5 [file formative-v10-e89518-s005.pdf]

Multimedia Appendix

Table S1 Ratings for each physical activity (PA) determinant and overall by the adolescents on 7-point Likert scale ranging from 'very hard' (1=strong barrier) to 'very easy' (7= strong facilitator). The central question was: "What makes it easy or hard to participate in sports and exercise for you?". Mean ratings, standard deviations (SD) and within group agreement (r_{wg}) are indicated for the overall sample.

Determinant (D): Description	Mean Rating (SD)	Overall r_{wg}
D1: Knowledge and awareness of health benefits	5.51 (1.47)	0.46
D2: Physical skills and fitness	5.25 (1.50)	0.44
D3: Habits and routines	4.99 (1.66)	0.31
D4: Early initiation	5,06 (1.50)	0.43
D5: Ability to navigate social dynamics	5.70 (1.44)	0.48
D6: Cognitive tiredness after school	4.28 (1.80)	0.19
D7: Financial resources of household	5.37 (1.87)	0.13
D8: Parental knowledge	5.34 (1.63)	0.34
D9: Commuting to school	4.63 (1.83)	0.16
D10: Inclusive activities at school during school	4.23 (1.92)	0.08
D11: Activities outside school after school	4.85 (1.83)	0.16
D12: Competition	5.00 (1.87)	0.13
D13: Transportation and distance to sport facilities	5.25 (1.45)	0.47
D14: Time after school hours	4.75 (1.91)	0.08
D15: Activities at school after school hours	3.74 (1.97)	0.03
D16: Social support from family	5.82 (1.32)	0.57
D17: Social support from teachers and school staff	4.41 (1.73)	0.25
D18: Social support from sport professionals	5.21 (1.87)	0.13
D19: Social support from friends and relatedness	5.34 (1.72)	0.26
D20: Role models	5.22 (1.54)	0.41
D21: Social media	4.74 (1.82)	0.17
D22: Emotions	5.38 (1.61)	0.36
D23: Fun activities	5.57 (1.42)	0.5
D24: Sport preferences vs popular sports	5.52 (1.59)	0.37
D25: Gender-specific activities	4.69 (1.72)	0.26
D26: Self-efficacy	5.68 (1.45)	0.47
D27: Self-esteem	5.54 (1.19)	0.65
D28: Goals	5.20 (1.50)	0.44
D29: Voluntary participation	5.20 (1.62)	0.34
Overall	5.09 (1.72)	0.26

Table S2 Quiz questions and responses by adolescents used to validate the researchers' interpretation of the perceived physical activity determinants among adolescents themselves.

Question		Categorical answer option		Responses (n)	Responses (%)
1	What do you do at school during breaks?	A	I prefer to rest, hang out with my friends or eat. Breaks are quite short.	62	76%
		B	I like to play sports with the ball or I walk outside. It keeps me active.	15	18%
		C	I want to play sports but the available sports are not very nice...	5	6%
2	What do you do at school after school?	A	I want to go home. I'd rather do other things, or be outside with friends.	66	78%
		B	I have to be somewhere else, such as working, internship or helping at home.	11	13%
		C	I like to stay if there are fun free sports. It gives me something to do.	8	9%
3	What do you want to do after school?	A	I prefer to rest or do other things	53	62%
		B	I often have to work or help out at my internship or at home	12	14%
		C	I have time and desire to exercise and exercise	20	24%
4	What do your teachers do to motivate you for sports?	A	They explain why sport is healthy and important. They motivate us.	27	33%
		B	Sometimes they talk about sports and health, but not very often.	31	38%
		C	They don't talk about it in class and don't know what I do for sports	23	28%
5	How do you go to school?	A	By bike or walking, which is already good exercise for me	22	27%
		B	By car or bus. It's more convenient...	36	43%
		C	I use a fat bike or an E-bike, so I don't move around much...	25	30%
6	What do you think about popular sports?	A	I think... mwah... I don't really like any sport	20	23%
		B	I like the same sports as many other people	51	59%
		C	I like sports, but the popular sports are not fun	16	18%
7	How far are the sports spots from you?	A	Close by, which makes it easy to start exercising	41	48%
		B	Far, so it's hard to get there	12	14%
		C	Far, so it's hard to get there	32	38%
8	What do your parents do to support you in sports? My parents...	A	... say things like "Sport is healthy" or "Well done!" or "You can do it!"	59	68%
		B	... sports or exercise with me. This makes sports more fun.	19	22%
		C	... almost never talk about sports. This makes me insecure.	9	10%
9	How important are friends to you in sports?	A	I like to play with my friends.	49	56%
		B	My friends prefer to do other things like chill or just hang out	22	25%
		C	I'm a bit in between.	16	18%
10	How much do you like sports?	A	I like sports! I enjoy playing and always have a good time!	42	48%
		B	It depends on the sport. Some are fun while others a bit boring	32	37%
		C	Sometimes sport can be boring for me. I don't always find it exciting...	13	15%
11	How confident are you about sports?	A	I don't really believe I'm good at sports	12	14%
		B	I believe I can exercise well	57	67%
		C	A little, but sometimes I doubt myself and my skills	16	19%
12	How do you feel about your body and exercise?	A	Good. I want to exercise to get fit or look good	68	81%
		B	Okay, but sometimes I worry about how I look while exercising	8	10%
		C	A little uncomfortable...	8	10%
