# Supplementary material for: Physical Activity Determinants Among Adolescents With Mild Intellectual Disability in Dutch Practical Education: Exploratory Sequential Mixed Methods Study
Source: JMIR Form Res. 2026 Sep 30;10:e89518. doi: 10.2196/89518 (PMC13626643; doi:10.2196/89518)
Supplement: Supplementary file 6 [file formative-v10-e89518-s006.pdf]

Multimedia Appendix

Table S1 Determinants of physical activity (PA) identified via qualitative analyses of data derived from expert interviews (E), teacher consultations (T), and adolescents' activities (A). Each PA determinant (first column) is mapped to relevant Capability-Opportunity-Motivation Behavior (COM-B) model components and specific Theoretical Domains Framework (TDF) domains (second column). The data source column indicates which stakeholder groups or literature identified each determinant.

PA Determinant		COM-B Component: TDF Domain	Data Source
D1	Knowledge and awareness of health benefits	Capability: knowledge	E, T, A
D2	Physical skills and fitness	Capability: skills	E, T
D3	Habits and routines	Capability: behavior regulation	E, T, A
D4	Early initiation	Capability: skills<br>Capability: behavior regulation	E, T
D5	Ability to navigate social dynamics	Capability: skills	E, T
D6	Cognitive tiredness (after school)	Capability: memory, attention, and decision process<br>[Opportunity: environmental context and resources]	E, T, A
D7	Financial resources of household	Opportunity: environmental context and resources	E, T
D8	Parental knowledge	Opportunity: social influences	E, T
D9	Commuting to school	Opportunity: environmental context and resources	E, T, A
D10	Inclusive activities at school during school	Opportunity: environmental context and resources	E, T, A
D11	Activities outside school after school	Opportunity: environmental context and resources	E, T, A
D12	Competition	Capability: skills<br>[Motivation: emotions]<br>[Motivation: goals]<br>[Opportunity: social influences]<br>[Opportunity: environmental context and resources]	E, T, A
D13	Transportation and distance to sport facilities	Opportunity: environmental context and resources	E, T
D14	Time after school hours	Opportunity: environmental context and resources	E, T, A
D15	Activities at school after school hours	Opportunity: environmental context and resources	E, T, A
D16	Social support from family	Opportunity: social influences	E, T, A
D17	Social support from teachers and school staff	Opportunity: social influences	A
D18	Social support from sport professionals	Opportunity: social influences	E, T
D19	Social support from friends and relatedness	Opportunity: social influences	E, T, A
D20	Role models	Opportunity: social influences	E, T, A
D21	Social media	Opportunity: social influences	E, T, A
D22	Emotions	Motivation: emotions	E, T, A
D23	Fun activities	Motivation: emotions<br>[Opportunity: environmental context and resources]	E, T, A
D24	Sport preferences vs popular sports	Motivation: emotions<br>[Motivation: social role and identity]<br>[Opportunity: environmental context and resources]	A
D25	Gender-specific activities	Motivation: emotions<br>[Opportunity: social influences]<br>[Opportunity: environmental context and resources]	E, T
D26	Self-efficacy	Motivation: beliefs about capabilities	E, T
D27	Self-esteem	Motivation: beliefs about capabilities<br>[Motivation: beliefs about consequences]	E, T, A
D28	Goals	Motivation: goals<br>[Motivation: intentions]	E, T, A
D29	Voluntary participation	Motivation: intentions<br>[Motivation: emotions]<br>[Motivation: reinforcement]	E, T, A

Table S2 Detailed maps of physical activity (PA) determinants in adolescents with mild intellectual disability (ID) derived from all qualitative sources, including literature, expert interviews, teacher input, and adolescent activities. Includes examples of participant quotes, descriptions of adolescents boards, and extracts of researcher's notes. Mapping is structured by theoretical concepts: by Capability-Opportunity-Motivation Behavior (COM-B) component and Theoretical Domains Framework (TDF) domains. We describe determinants as barriers and facilitators to PA.

COM-B Component	TDF Domain	Construct	Experts & Teachers	Adolescents
Capability	Knowledge	Awareness of the PA benefits to health	*Barriers:*<br>Low health literacy and lack of understanding of health benefits of PA due to the absence or delayed effects. "When they drink a liter of Coca-Cola, they don't understand what that means for their body." Expert-1<br>*Facilitators:*<br>Tailored, accessible, digital health education tools and methods (e.g., storytelling methods) improve understanding. "Make it into a story so that they will want to absorb, yes, the information. (...) so they would remember more than former traditional lesson." – Expert 12	*Barriers:*<br>Knowledge is superficial and sports focused; daily PA is not recognized. "[Adolescents] were surprised when their teacher indicated that daily activities (e.g., cleaning, walking) are also forms of PA. Only some indicated these activities as active in their timelines, even after the explanation from their teacher and researcher. Additionally, they struggle to elaborate on "why" questions related to the health benefits of PA, mostly mentioning concrete and proximal effects of PA (e.g., stronger muscles, weight loss)" – researcher notes<br>*Facilitators:*<br>Association of PA and health. "It is healthy for you and I want to stay healthy" Sensitizing input. Many (54) mentioned in brainstorming that PA helps them stay healthy and fit.
		Procedural knowledge	*Barriers:*<br>Lack of procedural knowledge of sport system (e.g., sign up, transport, funding). "Our system is very difficult for our adolescents. (...) They don't have a clue about policies (..) They don't know how to sign up and all that." – Expert 1<br>Limited exposure to diverse and enjoyable sports, where clubs discourage exploration. "They just don't really know which sports are there. So they have to get acquainted with many different sports to also find out what they like and what really fits with them..." – Expert 2<br>*Facilitators:*<br>School and programs that connect adolescents to sport opportunities and support exploration. "School does bring you into contact with sports." – Teacher-8 "(...) take them by the hand and show them how nice it is" – Expert-10	-
	Skills	Cognitive skills	*Barriers:*<br>Difficulties to understand complex sports and instructions. "they don't quite understand the game tactically" – Teacher-7<br>*Facilitators:*<br>Using simple language, small steps and repetition helps in understanding and developing skills. "Short sentences and do it and everything is alright." – Expert-7; "A lot of repetition" – Expert-5	*Barriers:*<br>Struggle to grasp abstract concepts, like the health benefits of PA. "Their health and PA knowledge is at the surface level and it seems that they do not grasp the reasons for the positive health outcomes of PA when asked about it." – RN
		Interpersonal skills	*Barriers:*<br>Some lack the necessary social skills to engage in group activities. "They are just not skilled enough to be social" – Teacher-8<br>*Facilitators:*<br>Developing social skills within physical education, in a safe environment, can help	-

		the adolescents better navigate group dynamics and simultaneously increase PA participation. "If you want to integrate social learning within physical education, then you need a safe spot climate." – Expert-8<br>Individual sports may be an alternative for adolescents who struggle with team dynamics and social interactions. "Boxing is more individual... you don't have to take the others into account." – Teacher-7	
	Practical skills	-	-
	Physical skills	Barriers:<br>Undeveloped skills due to late initiation. "They are not used to engaging in sports already from a young age. Then it continues to be harder for them because they are not used to throwing and catching the ball." – Expert-5<br>Facilitators:<br>Practice and repetition. "And a lot of repetition." – Expert-5	*Facilitators:*<br>Practice and repetition helps adolescents develop physical skills. "Adolescents indicated that they notice improvements in their skills when they do PA more often, especially related to specific sports like football." – RN
Memory, attention and decision process	Attention and cognitive tiredness	*Barriers:*<br>Shorter attention span, with struggles to focus on PA (or on acquiring knowledge or listening to instructions) for extended periods of time and in the long term, especially later in the day when feeling tired. "They get tired. (...) rather tired or dropped out ." – Teacher-7<br>Vulnerability to distractions by sedentary activities, incl. mobile phones, social media and TV. "There's a lot of distraction (…) from the social media." – Expert-2<br>*Facilitators:*<br>Restricting sedentary distractions. "Since last year we forbid having the mobile phones during school time... Instead of using their mobile phones during the breaks, they're now screaming and running around" -Expert 1	*Barriers:*<br>Difficulty to focus later in the day. "This was evident from the adolescents' behavior during the sessions, when comparing their ability to focus early in the morning and closer to the lunch break." - Researcher notes<br>Tiredness after school hours. "Many mention that they are tired after school and want to go home as soon as possible. In the timeline activity, many (62) used the "chilling" card after school and in the evening. It is evident in their timelines they they spend large amounts of time at home, not being active, after school hours. In one class, 9/13 adolescents mentioned that they often nap after school for 30 min to 2 hours."- RN
	Memory	*Barriers:*<br>Lower capability to remember sports rules and tactics. "The average sport is also just a matter of remembering things. a certain tactic." Teacher-6	-
Behavioral regulation	Habit formation and breaking; Action planning	Lack of early adoption of PA habits. "If you've never exercised, then you're probably not really going to do that now."<br>*Facilitators:*<br>Participation in structured and regular activities helps build a routine and decrease effort in action planning. "Because then indeed it's normal to go to the soccer club twice a week... and then it's just a routine and you're used to it." "We have a new program on school from the first years 12 years old, that every day they are going outside for 30 minutes. (…) They have to be active." – Expert-7<br>Difficulty in initiating and planning PA in leisure time on their own.	*Barriers:*<br>"Lack of early start on PA and sports" – Researcher notes<br>*Facilitators:*<br>Participation in structured activities. "In sensitizing, many (20) mentioned that they participate in sports x days per week, indicating that a structured schedule might decrease the threshold to participation and integrate PA in their weekly routines."- RN
	Self-monitoring	-	-

Opportunity	Social Influences	Social Support - General		*Barriers:*<br>Lack of support and social connections. "In the brainstorming phase, many adolescents (29) requested for a card expressing that they are alone. (…) they spend a lot of time alone, mostly commuting or at home in the afternoon/ evening. (…) during the anonymous notes from adolescents, one adolescent mentioned the lack of belief of others in him regarding PA." - RN
		Social Support - Family	*Barriers:*<br>Lack of support and participation in PA. "If the parents say, yeah, it's not important and I don't support it, then it's really hard for the kids sort of to go on a weekly basis." – Expert-10<br>Financial stress. "It's financial, it's social, it's psychological... there are often a lot of more important, more urgent issues, rather than getting your kid to the Sports Club." – Expert-10<br>Lack of parental knowledge on PA and health. "you have also educate the parents why it's necessary for their children to participate in sport because they don't know and they don't know what's best for their children, why it's best for their children." – Expert-8<br>Parental overprotection and fears of safety. "they can have also their own fears about. Letting their Childs going into sports because that can be happening. Bad things." – Expert-8<br>Inability to support in practical aspects, e.g. transportation, due to financial stress and time constraints. "If you don't have a car, then for the kids and for the parent, that already feels... mentally, I think, also hard." – Expert-5<br>Parental social segregation in Sport Clubs due to their kids disability.<br>*Facilitators:*<br>Educating parents about the benefits of PA and involving them in programs may facilitate their informed support to the adolescents. "An adaptation program for parents would be a very good idea (...) for stimulating sports participation." – Expert-8<br>Parents of these adolescents can help overcome barriers and improve inclusivity in Sport Clubs. "There are always parents with special needs kids... they organize all kinds of things and mobilize energy within the club." – Expert-8	*Barriers:*<br>Lack of support. "In the brainstorming activity, 3 out of 61 adolescents identified that family makes it harder to participate in PA. (…) One adolescent mentioned that his family failed to sign him up for swimming classes, having missed the deadline."<br>*Facilitators:*<br>Presence of support. "Family is often (22/ 59) mentioned in the sensitizing phase as people who make the adolescents enthusiastic about PA. Family is mostly and often (41/61) mentioned as a facilitator as well in their timeline boards, but not mentioned as often spontaneously in conversations or as their motivation to participate (3)."
		Social Support – Professionals (e.g., teachers, coaches)	*Barriers:*<br>Lack of training of coaches to work with adolescents with special needs, particularly at Sport Clubs. "They are not trained within our education system. (…) They don't understand that they should watch the group dynamics and intervene when something goes wrong." – Expert-8<br>*Facilitators:*<br>Coaches who establish a social connection and create a safe sports environment. "You need to understand them and create this caring and safe climate before he can do anything with the physical education part." – Expert-8<br>Child-centered coaching that focus on special needs rather than performance.	*Barriers:*<br>Teachers perceived as unsupportive. "Teachers do not follow the positive trend, being the only group that adolescents think mostly make it harder for them to participate in PA (22 out of 46). Similarly, only 2 adolescents mentioned their coaches or PE teacher has people who make them enthusiastic about sports in the sensitizing phase."- RN<br>Absence of a stable teacher relationship (e.g., drop-out or job, sickness). "One class with a particularly challenging behavior, did not use- at all - the teacher card. I noticed that this class did not have a

		"Child centered Coach is how (…) you help them through sports" – Expert-8<br>Specialized training programs for coaches and teachers. "Create a module or training for the knowledge part of these neighborhood coaches so they can be in their role." – Expert-8	fixed coordinator: multiple teachers were rotating the responsibility for the class. This might lead to a higher feeling of lack of support or even the feeling of absence of a teacher with an important role in their daily routines." - RN
	Social Support – Friends; Relatedness and group norms	*Barriers:*<br>Difficulties in forming social connections and networks, especially in environments not tailored to their special needs (e.g., sport clubs). "One barrier into going to the Sports Club is that they don't connect with the people there" (Expert-8).<br>Friends who lack interest, discourage participation, or prefer sedentary activities. "If they are supportive, then you are going to do some sport. If they aren't, then... it doesn't matter if they have friends in or outside of school" (Expert-7). "The norm in general is we don't like sports. And then it's very hard. And if you do have an interest for sports, then it's quite hard to make that known." – Expert-5<br>*Facilitators:*<br>Participating with friends and relatedness. "They do it because it's fun and because the friends are participating. That's a drive" (Expert-3).<br>General positive attitude towards PA. "If the norm in the friend group is we like sports, then everyone is like sports and there cannot be exceptions." – Expert-5	*Facilitators:*<br>Participating with friends and relatedness.<br>"Friends were often mentioned in the sensitizing phase as people who make the adolescents enthusiastic about PA (17) and as their motivation to participate (12). Friends are mentioned mostly and often as a facilitator to PA in the adolescents' boards (51/76). Adolescents want to do sports with their friends. This idea was expressed spontaneously by many during the sessions and in additional TDF questions. Moreover, when asked broadly about why they think sports are fun, they quickly mentioned their friends: "Het is gezellig met vrienden" (Dutch saying expressing a feeling of relatedness." - RN
	Modeling	*Barriers:*<br>Lack of active role models. "And if you don't get the stimulus of your surroundings or it's not common to go for a walk or sport on your own, then they don't have like a good practice. They see someone doing it." – Expert-3<br>*Facilitators:*<br>Active role models. "I think seeing movement is moving" – Teacher-7<br>Social media influencers as active role models. "He is follow[ing] a certain influencer, and then it's like a really supportive influencer. That can sometimes be a motivation to start." – Expert-5<br>Female role models may be relevant for girls. "Especially for girls, also have female coaches that are a model for the girls to stay in and come in because they are related to girls." – Expert-8	*Barriers:*<br>Absence of active role models. "In the sensitizing phase, 8 adolescents mentioned that nobody makes them enthusiastic about sports."- RN<br>*Facilitators:*<br>Sport idols and social media influencers. "Some (8) adolescents mentioned sport idols (e.g., professional football players) and social media influencers as motivators in sensitizing)." - RN
	Social norms (gender)	*Barriers:*<br>Sports opportunities are oriented towards male preferences<br>"One of the main things within our Dutch sport area is that it is boy-oriented. It's men-dominated." – Expert-8<br>*Facilitators:*<br>Design opportunities tailored to girls' preferences. "The youth worker I know, they sometimes do workshops on dancing with the girls." – Expert-3	-
	Alienation	*Barriers:*<br>Social exclusion from peers due to their perceived lower cognitive abilities. "They sometimes drop out because they (…) get a little teased that they're less smart."-Teacher-1	-

	Environment context and resources	Organizational – School Environment	*Barriers:*<br>Insufficient staff availability. “Teachers need a break too” – Expert-1<br>Lack of facilities and equipment. “For example, we also had a fitness room upstairs.(...) But now that it has become a classroom, it is no longer allowed. We also make it more difficult.” – Teacher-7<br>Fatigue after practical school lessons. “Adolescents who do an internship in the hospitality industry three days a week have been walking a lot all day. They really don’t feel like doing anything in the evening.” – Teacher-7<br>Sedentary commuting styles. Increase use of e-bikes. Higher difficulties for those living farther from the school in rural areas. “I see a lot of since last year I see very much and a lot of fat bikes parked downstairs and a... and a lot of E bikes.” – Expert-1 “Not all adolescents could go to school by bike (...) they have to use the bus or car.” – Expert-3<br>*Facilitators:*<br>Organic PA in practical lessons and internships. "Practical teaching is already very physical in terms of internships and stuff. They really have to work all day." – Teacher-1<br>PA opportunities during the school day. “the adolescents during the break time, they really like to go outside really like to get loose of their energy.” - Expert-3<br>School support in connecting adolescents with neighborhood coaches and sport opportunities outside the school environment. "40% of the schools say they have a connection with the coaches." – Expert-6	Barriers:<br>School focus on sedentary educational activities and insufficient opportunities to PA. “The school period is seen as sedentary, only a few (5) adolescents reported being active during the breaks. Organized activities by the school were not mentioned.”- RN<br>No PE lessons in curriculum for older adolescents. “Older adolescents no longer have PE classes at school, which can reduce their PA levels. Only some also mention spontaneous PA during the breaks, e.g. playing football.” – RN<br>*Facilitators:*<br>PE lessons. “Many (34) identified PE classes as an active environment in their boards”<br>Practical lessons and field internships with organic movement. “While most adolescents did not acknowledge practical lessons as a source of PA, others said they already move enough during the day without participating in sports”<br>Daily active commute. “Many (51) mentioned active commutes to school (e.g., by bike) in their timeline board” - RN
		Organization – Outside School Environment and Resources	*Barriers:*<br>Difficulties with transportation, especially in rural areas. "They experience quite a big barrier to start with sports because of the distance, because they have to arrange getting to a Sports Club."<br>Difficulties in adaption to sport clubs. The inflexible schedule of organized sports and the strict coaching style oriented to sport performance can cause drop outs. "Everything that's in the strictness of organized sport is also driving them away. But they still like activities." "A lot of children drop out in sports... because the coach doesn't coach in a way that fits the development of the child cognitively or socially."<br>*Facilitators:*<br>Inclusive local sports services and programs offering structured opportunities for PA, including and beyond sport clubs(e.g., youth workers, neighborhood coach). "You have these local sports services... committed to getting people more active in the neighborhood." – Expert-10	*Facilitators:*<br>Popular and structured sports and activities. “Many adolescents mentioned in the sensitizing phase participating in sports like football (27) and kickboxing (17). In their boards, adolescents (25) mention participating in sport clubs (structured PA).<br>Flexible and individual activities. “Some (18) mentioned going to the gym and others (15) mentioned engaging in PA spontaneously by going outside with friends and family after school hours. Activities range from just walking around and hanging out, to actually playing sports like football.” - RN
		Household environment and resources	*Barriers:*<br>Financial difficulties. “Their primary focus is on getting up in the morning, getting food on the table, work per week, so getting an income and then sports and physical activity is, yeah, not a priority for most.” – Expert-5<br>Need to help the family with a part-time job. "If you have a part-time job and sports, that's too much, because then they can't chill anymore." – Teacher-1	Barriers:<br>PA at home is very rare. “Only 3 adolescents mentioned home as an active environment in their boards” - RN<br>Lack of financial resources. “Lack of money to participate in sports is mentioned by a few adolescents in the sensitizing (3) and in brainstorming (2) phases, mostly in anonymous opportunities.” - RN

	Rare participation in PA at home, in leisure time and alone. “Never heard of these adolescents doing it”-Expert-5	
Time	Barriers:<br>After school hours, preference for relaxation and sedentary activities. "When the school is out, they want to go. They want to chill in the neighborhood." – Expert-7<br>"It should not take their leisure time." – Expert-9<br>Time-rigid organized sports and competing responsibilities (e.g., part time jobs, internships). “If you have a part-time job and sports, that’s too much, because then they can’t chill anymore.” – Teacher-1<br>Facilitators:<br>Integration of PA in daily school schedule, or immediately after. "Every day from 10:00 to 10:30 we're going to walk around the school and everyone has to join." – Expert-5<br>“That's much easier for them because then they are already at school, so once they go home, this threshold gets bigger.” – Expert-2<br>Time-flexible activities (e.g., gym). "You see that also the other organized sports like the more the commercial ones or the fitness one that creates opportunities for the older children because they can come in when it fits them." Expert-8	Barriers:<br>After school hours, preference for relaxation and sedentary activities. “Many adolescents mention that they are tired after school or simply want to go home as soon as possible. Many (62) used the “chilling” card after school and in the evening, most not recognizing it as an active period associating it with rest or sedentary activities (e.g., gaming, being on the phone, watching series)” - RN<br>Time-rigid organized sports and competing responsibilities (e.g., part time jobs, internships). “Older adolescents (14) mentioned work, as part time jobs that take their time away from sports.” - RN<br>*Facilitators:*<br>Time-flexible activities. “Flexible activities, like being active outside with friends, going to the gym to do fitness or martial arts seem to be popular.”- RN
Policy, programs and other resources	*Barriers:*<br>Lack of school PA programs and policies. "Without policy, you see it's really depending on a specific person and when the person leaves, it falls down again." – Expert-9<br>Lack of system-thinking for behavior change programs and policies. "It's a complex multi-layer approach and only targeting kids or only targeting policy... I would strongly recommend against just that." – Expert-10<br>Centralization of government funds to sport clubs, neglecting school-based or alternative PA options. "For a lot of kids, the Sports Club is not the place for them. So I think what we should do is really look at other ways of being physically active that suits them more." – Expert-5<br>Need to provide financial documentation to access public subsidies for PA. “It was a big barrier for parents that they needed to show their finances.” – Expert-5<br>*Facilitators:*<br>Subsidies for sport participation for people with a low financial resources.<br>Programs that facilitate transportation to sport facilities. “Sometimes clubs also organized the transportation” – Expert-8<br>Commitment and financial support from local governments.<br>"You need local networks in order to make it work. (...)the local government is involved because of the finances, the funding." – Expert-10<br>Integrating PA into the school health policy, involving teachers and sustaining programs with government funding	-
Environmental stressors	-	-

Motivation	Reinforcement	Contingencies and Sanctions	*Barriers:*<br>Top-down mandatory activities and pressure to participate without adolescents' input can be counterproductive. "Forcing them to is the big not to do." – Expert-5<br>Strictness of organized sport and sport clubs. "A lot of children drop out in sports... because the coach doesn't coach in a way that fits the development of the child cognitively or socially."<br>*Facilitators:*<br>Voluntary participation and autonomy. "If they can choose and it's voluntarily? Then it's not something I'm telling them you have to do this. You have to do that." – Expert-1	*Barriers:*<br>*Facilitators:*<br>Autonomy in choosing activities. "Adolescents seem to have a stronger desire for deciding what to do in their own time (related to autonomy and self-determination). When asked what they do after school, they expressed a desire to do what they want of their leisure time."- RN
		Rewards and Incentives	*Barriers:*<br>*Facilitators:*<br>Rewards, incentives and encouragement for participation. "Sometimes a compliment or the fact that they achieved a challenge is the reward in itself. And sometimes it's, they can choose." – Expert-1<br>Gamification delivering positive rewards. "It's a health application, so you count your steps and you get coins for that. And with the coins you can buy nice healthy stuff again." – Expert-9	-
		Reinforcement and consequents	*Facilitators:*<br>Enabling small successes and recognition. "It's important to have smaller steps. So they get more success experiences even though it was a small step." – Expert-5<br>"They need positive feedback or positive input" – Expert-1	
	Emotion		*Barriers:*<br>Pressure and anxiety in competitive environments. "Sports includes that sport also excludes...sports is healthy, that's sports is also unhealthy." – Expert-8<br>Fear of joining sports clubs or PA activities due to perceived lack of skills, being judged by others, and navigating unknown experiences and environments. "most of them are afraid to go everywhere"- Expert-7<br>Shame related to not being as skilled as others and of being seen exercising. "(...) they are ashamed (...) You're so aware of of the people around you and what they might think of you (...) all kids have some doubt on how good they are and how how does it look when I run, how does it look when I throw?"- Expert-5<br>Disgust with sweating in public and the inability to shower after PA. "Adolescents didn't want to. No, ma'am, because then I'm going to sweat." Teacher-7<br>*Facilitators:*<br>Fun and teambuilding activities rather than competitive or performance oriented. "They do it because it's fun and because the friends are participating." – Expert-1<br>Innovative and creative activities, such as augmented reality games (e.g., magic trails project) or mixed sports (e.g., Shuffle sports project), can make physical activity more appealing. "Pokémon Go, it's a very nice example." – Expert-9	*Barriers:*<br>Tiredness. "Many adolescents (38) mentioned feeling tired during and after PA, but it is unclear if they think of it with a positive/ negative sentiment.<br>Negative emotions. "Only 2 and 5 adolescents expressed clearly negative sentiments related to PA in the sensitizing and brainstorming phases."- Researcher notes<br>Loosing and disappointment in competition. "In the brainstorming phase, losing is mentioned 2 times as something that makes it hard for them to participate in sports." - RN<br>*Facilitators:*<br>Enjoyment and Fun. "In the sensitizing phase, when asked what makes them do sport and exercise, many (21) spontaneously report that they do it because it is fun.<br>In the brainstorming phase, the word "fun" is used 19 times to describe sports. Similarly, the word was repeated several teams in conversations during the session as the major reason why they participate in PA."- RN<br>Feeling energetic. "Many adolescents mention feeling good, happy and energetic in sensitizing (35) and brainstorming (30)." - RN<br>Feeling occupied and calm. "Some adolescent (5) mention PA as a positive distraction, with good mental health outcomes."- RN

			Competition and challenge. “Competition and winning were mentioned only once as a positive motivator for PA, particularly sports. In the brainstorming phase, it is mentioned 3 times as something that helps them to do more sports.” - RN
Social Role and Identity	Social/ Group Identity	*Barriers:*<br>Negative attitude of social circle towards PA with low participations and desire for a sense of belonging. "The norm in general is we don't like sports. And then it's very hard. And if you do have an interest for sports, then it's quite hard to make that known. (...) So if the norm in the friend group is we like sports, then everyone is like sports and there cannot be exceptions.” – Expert-5<br>Association of owns identity with sedentary activities and not ‘sportive’.<br>*Facilitators:*<br>Positive attitude of social circle towards PA with high participation and desire for a sense of belonging.	*Barriers:*<br>Association of own identity with sedentary activities and not “sportive”. “adolescents who do not see themselves as sporty are less likely to be motivated to start. This was also shown by their interest in participating in this research’ sessions.” - RN<br>*Facilitators:*<br>Association of own identity with sports, commonly a particular sport (e.g., football, kickbox). “Many adolescents just replied with the word “football” to every question: what do they do? Football. What makes them enthusiastic? Football. Who make them enthusiastic? Football players.” - RN
	Leadership	-	-
Beliefs about capability	Self-efficacy	*Barriers:*<br>Low self-efficacy related to PA and in general. "Adolescents who don't feel they fit in a team or aren't good at sports often avoid participating because they feel weaker or less capable."– Teacher-6<br>Past negative experiences and failures related to sports and education. “A lot of our adolescents had a lot of disappointments during their school career...” – Expert-1<br><br>*Facilitators:*<br>Beliefs about PA skills. "If you're good at sports, you remain fanatical and active.”– Teacher-8<br>Enabling small successes through scaffolding and recognition. "It's important to have smaller steps. So they get more success experiences even though it was a small step." – Expert-5 “They need positive feedback or positive input” – Expert-1 "It's important to have smaller steps so they get more success experiences even though it was a small step."	-
	Self-esteem	*Barriers:*<br>Low self-esteem related to body image. “All kids have some doubt on how good they are and how it look when I run, how does it look when I throw?” – Expert-5<br>*Facilitators:*<br>A positive and safe PA environment. “they think “ohh, but my body ... I can do do this” so that the positive attitude that you can all do all kind of things.” – Expert-8	*Barriers:*<br>Low self-esteem related to body image. “Some (4) adolescents mentioned losing weight as an end to PA and it seems that it has a negative sentiment”- RN<br>*Facilitators:*<br>Achievement. “Some adolescents (3) say they feel proud of themselves in participating in PA.” - RN
Intentions	Intention	*Barriers:*<br>Top-down mandatory activities and pressure to participate without adolescents’ input can be counterproductive. "Forcing them to is the big not to do." – Expert-5	-

		Difficulty in sustained intention to participate in PA. "They have the focus now on something but... Ah. Two weeks, 3 weeks and then OK, it's not so funny anymore." – Expert-7<br>*Facilitators:*<br>Voluntary participation and autonomy. "If they can choose and it's voluntarily? Then it's not something I'm telling them you have to do this. You have to do that." – Expert-1	
Goals	Goal-setting	*Barriers:*<br>Difficulty in setting distal goals. "The biggest challenge is to uh, incorporate it in their lives and give it a meaning for them for the longer term."- Expert-1 "They yeah, indeed, they they do know the health benefits, but it's but it's not a priority. And and I think and that's even more for the praktijkonderwijs than for the VMBO that looking in the future is is very difficult. So they they are not interested in sports today." – Expert-5<br>*Facilitators:*<br>Emphasizing short-term benefits like feeling stronger and more energetic. "Finding goals or targets that people have short-term benefit from, I feel stronger. I feel more energetic." – Expert-11<br>Setting short-term and achievable goals. "A challenge that ends within a week or in a couple of days to begin with." – Expert-1<br>Personalizing goals to individual needs and preferences. "There are different ways to personalize content... in terms of goals, so if I need to be more active, how much more do I need to move?" – Expert-11	*Barriers:*<br>Lack of long-term goals related to PA. "In the sensitizing phase, when asked what makes them do sport and exercise, only 3 adolescents mentioned their goals and dreams. This indicates that adolescents lack goals related to PA, in the long term."- RN
Optimism	Optimism	-	-
Beliefs about consequences	Outcome expectancies	*Barriers:*<br>Failing to grasp the long term health outcomes of a sedentary lifestyle. "They don't experience health problems. So that's not their motivation, I guess, to be physically active." – Expert-1<br>Beliefs that their appearance might be judged by others while engaging in PA. "Kids don't like to exercise outside on the playground because then they can be seen by a lot of others." – Expert-6	*Facilitators:*<br>Expecting improvements in health and fitness. "In the sensitizing phase, many adolescents (21) also reported that getting fit and healthy is something they like about sports (21)." - RN<br>Expecting improvements in body image. "In their brainstorming boards, some adolescents report believing that PA contributes to improving their body image (15), mentioning losing weight and having stronger muscles." -RN
