# Supplementary material for: Physical Activity Determinants Among Adolescents With Mild Intellectual Disability in Dutch Practical Education: Exploratory Sequential Mixed Methods Study
Source: JMIR Form Res. 2026 Sep 30;10:e89518. doi: 10.2196/89518 (PMC13626643; doi:10.2196/89518)
Supplement: Supplementary file 8 [file formative-v10-e89518-s008.pdf]

Good Reporting of A Mixed Methods Study (GRAMMS)

Guideline	Page
Describe the justification for using a mixed methods approach to the research question	4-5
Describe the design in terms of the purpose, priority and sequence of methods	4-5
Describe each method in terms of sampling, data collection and analysis	Sampling: 3-4; 8; 10<br>Qual. Data collection and analysis: 5-8<br>Quant. Data collection and analysis: 8-10
Describe where integration has occurred, how it has occurred and who has participated in it	4
Describe any limitation of one method associated with the present of the other method	23-24
Describe any insights gained from mixing or integrating methods	4; 21

O'Cathain A, Murphy E, Nicholl J. The quality of mixed methods studies in health services research. J Health Serv Res Policy. 2008;13(2):92-98.
